# A randomised controlled trial of losartan as an anti-fibrotic agent in non-alcoholic steatohepatitis

**DOI:** 10.1371/journal.pone.0175717

**Published:** 2017-04-18

**Authors:** Stuart McPherson, Nina Wilkinson, Dina Tiniakos, Jennifer Wilkinson, Alastair D. Burt, Elaine McColl, Deborah D. Stocken, Nick Steen, Jane Barnes, Nicola Goudie, Stephen Stewart, Yvonne Bury, Derek Mann, Quentin M. Anstee, Christopher P. Day

**Affiliations:** 1 Institute of Cellular Medicine, Faculty of Medical Sciences, Newcastle University, Newcastle upon Tyne, United Kingdom; 2 Liver Unit, Newcastle Upon Tyne Hospitals NHS Trust, Freeman Hospital, Newcastle upon Tyne, United Kingdom; 3 Institute of Health and Society, Faculty of Medical Sciences, Newcastle University, Newcastle-upon-Tyne, United Kingdom; 4 Department of Pathology, Newcastle Upon Tyne Hospitals NHS Trust, Royal Victoria Infirmary, Newcastle upon Tyne, United Kingdom; 5 Medical School, National & Kapodistrian University of Athens, Athens, Greece; 6 Newcastle Clinical Trials Unit, Faculty of Medicine, Newcastle University, Newcastle upon Tyne, United Kingdom; 7 Faculty of Health and Medical Sciences, University of Adelaide, Adelaide, Australia; 8 Department of Gastroenterology, Mater Misericordiae University Hospital, Dublin, Ireland; The Chinese University of Hong Kong, HONG KONG

## Abstract

**Introduction:**

Non-alcoholic fatty liver disease (NAFLD) is a common liver disease worldwide. Experimental and small clinical trials have demonstrated that angiotensin II blockers (ARB) may be anti-fibrotic in the liver. The aim of this randomised controlled trial was to assess whether treatment with Losartan for 96 weeks slowed, halted or reversed the progression of fibrosis in patients with non-alcoholic steatohepatitis (NASH).

**Methods:**

Double-blind randomised-controlled trial of Losartan 50 mg once a day versus placebo for 96 weeks in patients with histological evidence of NASH. The primary outcome for the study was change in histological fibrosis stage from pre-treatment to end-of-treatment.

**Results:**

The study planned to recruit 214 patients. However, recruitment was slower than expected, and after 45 patients were randomised (median age 55; 56% male; 60% diabetic; median fibrosis stage 2), enrolment was suspended. Thirty-two patients (15 losartan and 17 placebo) completed follow up period: one patient (6.7%) treated with losartan and 4 patients (23.5%) in the placebo group were “responders” (lower fibrosis stage at follow up compared with baseline). The major reason for slow recruitment was that 39% of potentially eligible patients were already taking an ARB or angiotensin converting enzyme inhibitor (ACEI), and 15% were taking other prohibited medications.

**Conclusions:**

Due to the widespread use of ACEI and ARB in patients with NASH this trial failed to recruit sufficient patients to determine whether losartan has anti-fibrotic effects in the liver.

**Trial registration:**

ISRCTN 57849521

## Introduction

Non-alcoholic fatty liver disease (NAFLD) is a common liver disease worldwide, which has dramatically increased in prevalence due to the obesity epidemic [1]. In some countries, up to one third of the population have NAFLD, although many remain undiagnosed [2, 3]. NAFLD is defined histologically when >5% of hepatocytes are steatotic in the absence of a secondary cause, such as excessive alcohol consumption or the use of steatogenic drugs [4]. There is a spectrum of pathology in NAFLD, ranging from steatosis (fat without hepatocellular injury) through to non-alcoholic steatohepatitis (NASH; steatosis with hepatocellular injury and lobular inflammation) and ultimately to cirrhosis [5]. Overall, approximately 40% of patients with NAFLD develop progressive fibrosis, which results in cirrhosis in 20%, putting patients at risk of complications, such as hepatocellular carcinoma and liver failure [6–10]. The most important prognostic factor in patients with NAFLD is histological stage of liver fibrosis, and subjects with advanced fibrosis have increased risk of liver-related and all-cause mortality [11–13]. Therefore, a therapy that can reverse liver fibrosis has the potential to reduce risk of liver-related complications.

Currently, the mainstay of treatment for NAFLD is lifestyle modification, aimed at weight loss and increased activity, as there is no licenced liver-specific therapy [4, 14]. Weight loss of greater than 10% of body weight has clearly been shown to be beneficial, with 90% who achieve this having resolution of steatohepatitis and 45% having regression of fibrosis [15]. Moreover, all types of exercise can reduce hepatic steatosis and improve metabolic profile [16]. Unfortunately, despite the known beneficial effects of lifestyle modification, many patients fail these interventions and remain at risk of fibrosis progression.

Liver fibrosis is a complex dynamic process that leads to deposition of matrix proteins, such as collagens, elastin and proteoglycans, in the liver [17]. During fibrogenesis hepatic stellate cells become activated in response to pro-inflammatory cytokines, and transform into hepatic myofibroblasts (HMS), which are the major matrix protein producers in the liver. It is known that HMS have a local renin-angiotensin system that, when activated, continuously produces angiotensin II, which acts in an autocrine manner and stimulates fibrogenesis [18, 19]. Importantly, it has been shown in animal models of NASH and other liver diseases that treatment with angiotensin converting enzyme inhibitors (ACEI) and angiotensin II receptor blockers (ARB) can switch off this profibrogenic state and lead to regression of fibrosis [20–23].

Despite this compelling experimental evidence and the wide availability of well-tolerated, inexpensive ACEI or ARBs, few human studies have been conducted assessing the potential anti-fibrotic effects of these drugs in patients with NAFLD [24–26]. The initial aim of this randomised controlled trial was to assess whether treatment with losartan for 96 weeks slowed, halted or reversed the progression of fibrosis in an adequately powered study in patients with NASH. However, enrolment to this study was slower than expected and recruitment was halted early, when 45 patients were randomised. The revised aims of this study were aligned with the aim of pilot trials to: (1) to assess the proportion of patients who were eligible for the study and reasons for non-eligibility to inform the conduct of future clinical trials in this field; (2) to assess the baseline demographics of the study population and; (3) to describe histological and biochemical outcomes in the study cohort.

## Methods

### Study design

This was a double-blind randomised-controlled trial of Losartan 50 mg once a day versus placebo for 96 weeks in patients with histological evidence of NASH. The study recruited patients from 11 centres across England between the 6^th^ July 2011 to 19^th^ October 2012. The Sunderland Ethics Committee approved the study (REC Reference: 10/H0904/8), and all patients provided written informed consent.

### Inclusion/Exclusion criteria

Adults (aged 18+), with steatohepatitis and fibrosis (NASH Clinical Research Network CRN [NASH CRN] criteria stage 1–3 [27]), resulting from NAFLD were included in the study. The main exclusion criteria were: excessive consumption of alcohol (>21 units per week for males or >14 units per week for females), use of ACEI or ARBs in the previous year, poorly controlled diabetes (Haemoglobin A1C >15%), change in diabetes regimen in the last 3 months, use of steatogenic medications or drugs that might alter the natural history of NASH (eg pioglitazone, vitamin E, GLP-1 analogues etc) or weight loss of >5% body weight within the last 6 months. (Full inclusion/exclusion criteria are shown in S1 File).

### Randomisation and outcome measures

Patients were allocated in a 1:1 ratio to Losartan 50 mg once a day or matched placebo for 96 weeks. Randomisation was via a centralised blocked allocation system, and patients, treating clinicians and those involved in outcome assessment were blinded to treatment group. Since diabetes is a known major risk factor for progression of fibrosis, patients were stratified by presence of diabetes (defined according to the 2004 ADA criteria [28] or if they were taking an oral hypoglycaemic drug or insulin) to avoid imbalances between groups with respect to this potential confounder. All study participants (both arms) were given standard advice with regard to diet, exercise and weight maintenance, which was the recommendation to undertake 150 minutes of exercise/per week, combined with a reduction in intake of 500 kcal per day [29].

The original primary outcome measure for the study was change in histological fibrosis stage (NASH CRN) from pre-treatment (within 6 months of randomisation) to end-of-treatment (96 weeks). The 'Responder rate' for placebo and losartan was defined as end-of-treatment liver fibrosis stage less baseline liver fibrosis stage. The secondary outcomes for the study were to determine:

1. whether Losartan can prevent clinical deterioration in NASH.
2. whether treatment with Losartan affects quality of life in patients with NASH.
3. the association between serum and histological markers of fibrosis in patients with NASH, over the trial period using the ELF test [30] and other simple non-invasive fibrosis markers [31].

Patients were reviewed at baseline, weeks 1, 4, 24, 48, 72, 96 (end of treatment) and 108 (end of study). Investigations conducted at each visit included: weight, Body Mass Index (BMI), waist circumference, routine biochemical blood tests, Enhanced Liver Fibrosis (ELF) test, fasting glucose, insulin and lipids and quality of life questionnaires (Chronic Liver Disease Questionnaire (CLDQ) and SF-36).

### Histological assessment

Percutaneous liver biopsies were performed as per unit protocol at the sites. Baseline and end-of-treatment liver biopsies were centrally assessed by two independent expert hepatopathologists (ADB and DT) using the NIDDK-sponsored NASH Clinical Research Network (NASH CRN) criteria [27]. The NAFLD activity score (NAS) was graded from 0 to 8 including scores for steatosis (0–3), lobular inflammation (0–3) and hepatocellular ballooning (0–2). ‘NASH’ was defined as steatosis with hepatocyte ballooning and lobular inflammation +/- fibrosis. Fibrosis was staged from F0 to F4, according to NASH CRN [27], on sirius red fast green-stained sections. Discrepancies between observers’ histological scores were solved by reviewing the slides and reaching consensus. The trial pathologists were blinded to the baseline or end of treatment status and treatment assignment.

### Sample size calculation and recruitment strategy

Natural history studies suggested that, over a two year period, fibrosis progresses in around 25% of patients with NAFLD [32][33]. There was also regression in approximately 25% of patients so the mean rate of progression throughout the population is very close to zero. Pilot data, from a small trial of Losartan in patients with chronic hepatitis C suggested that the proportion that progress could be brought to nearly 0%, with regression of fibrosis in up to 50% of patients [34]. A further study giving Losartan for 48 weeks in seven patients with NASH showed a one point regression in fibrosis stage in four of seven patients with no progression in the other three [24]. The original sample size calculation is presented in the trial protocol (see S1 File) and is based on observing a clinically relevant difference in mean fibrosis score between Losartan and placebo of 0.42 with assumed standard deviation (SD) of 0.84, 90% power and significance of 0.05 (two tailed test). The original recruitment target was 170 patients with 96 weeks follow-up, inflated to 214 patients for anticipated attrition. We estimated that 450 individuals would need to be screened to recruit this number of patients. Initially, seven units were approached and suggested that they could screen at least four patients per week, and therefore recruitment was scheduled to take a maximum of six months between the seven centres. To allow time for centre initiation, 12 months was factored in for recruitment. Since the start of recruitment, the observed rates of eligibility and consent were well below expected so the number of sites was expanded to 11 and the recruitment period extended.

### Revised objectives

Unfortunately, due to inadequate recruitment to the study funding was withdrawn in October 2012 (at that stage 45 patients had been recruited). It was agreed that patients who were already recruited could complete the study to help inform the design of future studies. Patients were given written information about the change in trial objectives and were given the option of withdrawing at any time. The main revised aims of the study were to assess the:

- Proportion of patients who were eligible for the study and reasons for non-eligibility
- Proportion of eligible patients who agreed to be randomised
- Baseline demographics
- Level of data completeness
- Measures of central tendency and spread for all study outcomes
- Histological and biochemical outcomes in the study cohort

### Analysis of the reasons for slow recruitment

In order to investigate reasons for slow recruitment a detailed retrospective analysis of all patients who had a liver biopsy for suspected NAFLD from 6 months prior to recruitment commencing to the end of study (Jan 2011-Sept 2012; 21 months) was performed in Newcastle. Histology results and medical notes were reviewed for all these patients to determine potential eligibility and reasons for non-eligibility.

As use of an ACEI or ARB in the last year was an exclusion criterion for entry to the trial we also analysed prescribing rates for the drugs in Newcastle, before and during the trial. This analysis was conducted using a Primary care research database, which contained comprehensive prescribing information for 149,097 patients over the study period.

### Statistical analysis

The analysis was conducted on an intention-to-treat (ITT) basis. The primary analysis of efficacy was based on change in fibrosis score, from baseline to end-of-treatment (96 weeks). The planned analysis was to assess differences between placebo and intervention groups using analysis of covariance, with baseline fibrosis score as a covariate along with age, body mass index and presence of diabetes (factors previously associated with increased risk of progression). However, in view of inadequate recruitment and the lack of statistical power the analysis plan was changed to have a descriptive analysis, without conducting formal comparative statistical tests.

## Results

### Recruitment

**Fig 1:**
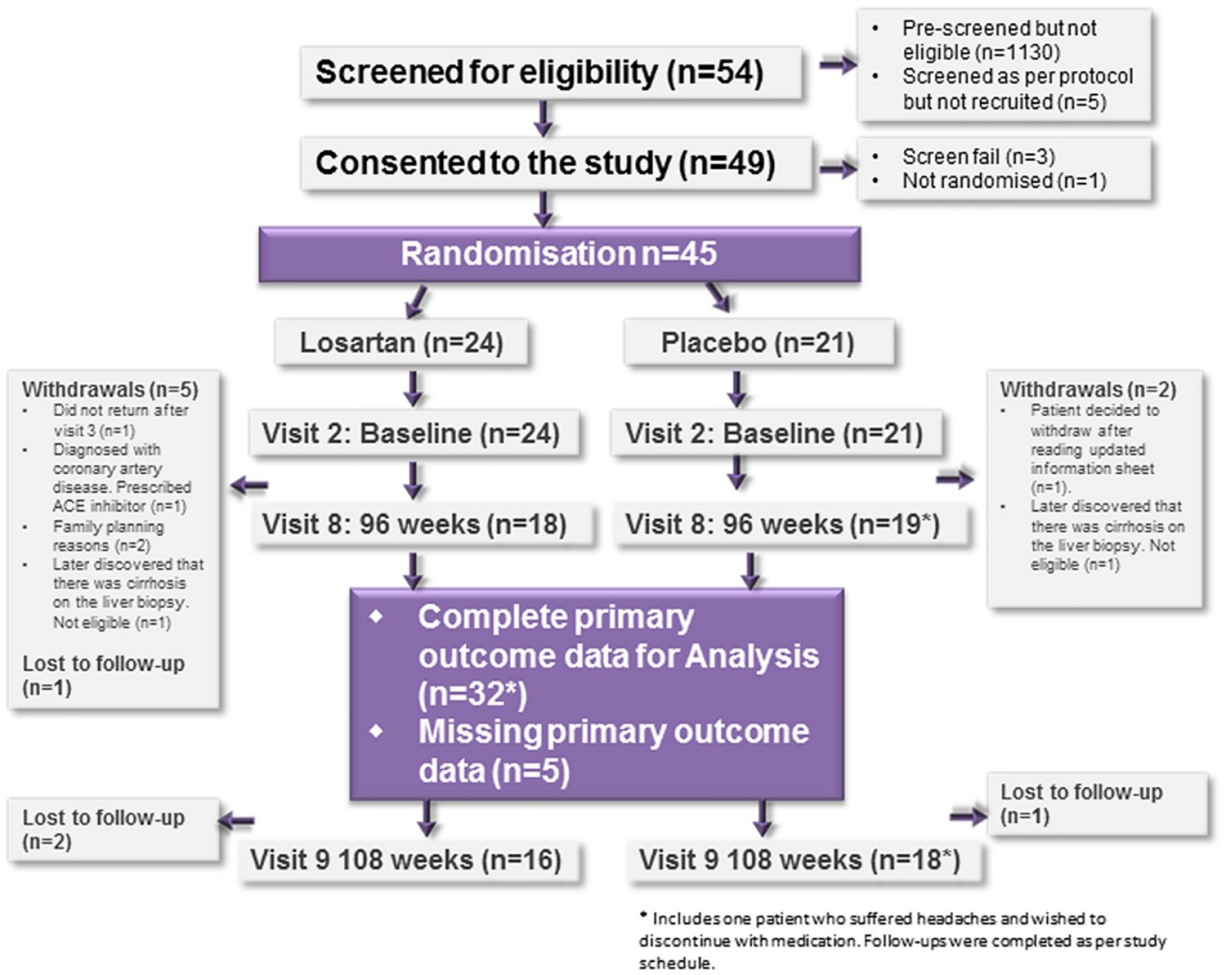
Consort diagram of patient recruitment and retention in the study.

**Fig 2:**
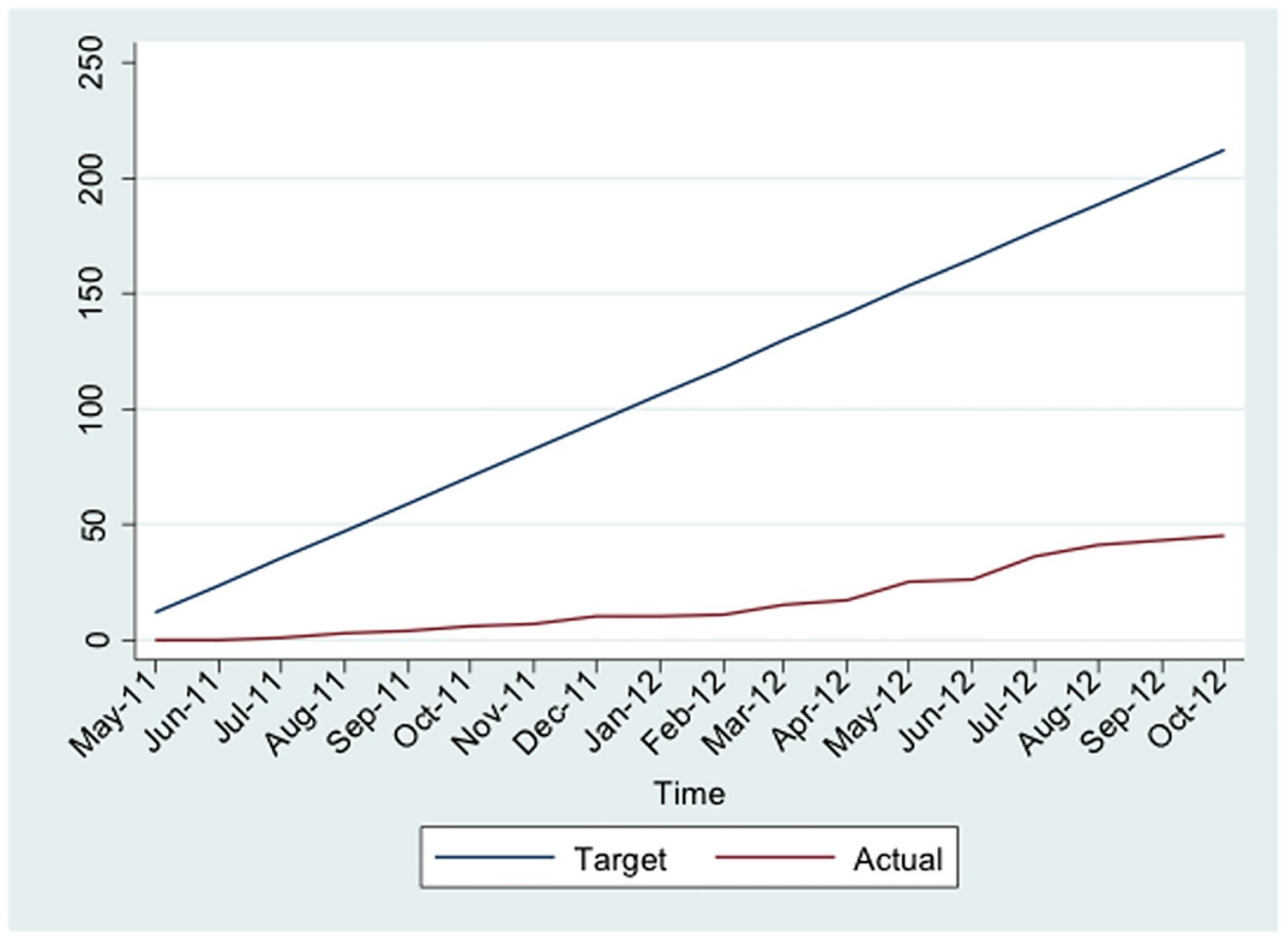
Cumulative recruitment to the study by month.

The CONSORT diagram shown in Fig 1 summarises recruitment to the trial and retention of patients the over the course of the study. Recruitment to the study began in July 2011, with all seven of the original sites open by November 2011. However, recruitment was slower than expected (Fig 2) so an additional 4 sites were added between March 2012 and June 2012. Despite this, recruitment targets were not met nationally and so enrolment was curtailed, finishing in October 2012. Patients who were already randomised were permitted to complete the study. Overall, a total of 54 patients were screened (although 1130 had been ‘pre-screened’ and found to be ineligible) for inclusion to the study and 45 were subsequently randomised (24 Losartan and 21 placebo). A total of 32 (71%) patients (15 Losartan and 17 placebo) had the follow up liver biopsy at 96 weeks including one patient who withdrew from trial treatment but agreed to follow up. Five patients attended the end of treatment visit but did not provide a liver biopsy, 7 patients withdrew completely from the study (1 patient did not return after visit 3, 2 patients later found to be cirrhotic and was therefore ineligible, 2 had family planning reasons, 1 diagnosed with coronary artery disease and commenced on an ACEI, 1 withdrew after reading updated patient information sheet) and 1 patient was lost to follow up.

### Baseline demographics of the study population

A summary of the baseline demographic and clinical characteristics for the 45 randomised patients is shown in Table 1. Overall the median age of the cohort was 55 years (range 21–76), 56% were male and 60% were diabetic. All patients in the study were overweight or obese and the median BMI was 32.9Kg/m^2^ (range 26.1–45.2). The median stage of liver fibrosis was 2 (IQR 2–3). The median NAS score was 5.5 (IQR 4–6) and 29 (91%) of enrolled patients had histological evidence of NASH when the slides were reviewed by the study pathologists.

**Table 1:** Baseline demographic and clinical characteristics, by randomised treatment arm.

Variable	Losartan (n = 24)		Placebo (n = 21)		Total (n = 45)	
	n	Summary	n	Summary	n	Summary
Gender n = male (%)	24	13 (54%)	21	12 (57%)	45	25 (56%)
Age (years)	24	58 (25–75)	21	45 (21–76)	45	55 (21–76)
Caucasian ethnicity n (%)	24	21 (88%)	21	19 (90%)	45	40 (89%)
Diabetes n (%)	24	15 (63%)	21	12 (57%)	45	27 (60%)
Weight (kg)	24	85.1 (74.2–121.0)	21	96.7 (61.6–132.5)	45	93.1 (61.6–132.5)
BMI (kg/m^2^)	23	32.8 (26.1–43.4)	21	34.1 (26.5–45.2)	44	32.9 (26.1–45.2)
Glucose (mmol/l)	24	6.0 (4.4–17.1)	20	6.2 (3.6–15.9)	44	6.0 (3.6–17.1)
ALT (U/L)	24	52.5 (21–136)	21	65 (33–135)	45	59 (21–136)
AST (U/L)	21	35 (14–102)	18	46 (30–70)	39	43 (14–102)
Gamma GT (U/L)	24	70 (18–355)	21	62 (23–256)	45	64 (18–355)
ALP (U/L)	24	89.5 (44–173)	21	72 (49–116)	45	84 (44–173)
Creatinine (μmol/l)	24	75.5 (48–105)	21	72 (5–97)	45	74 (5–105)
Triglyceride (mmol/l)	23	1.7 (0.9–7.9)	19	2.0 (0.4–4.4)	42	1.9 (0.4–7.9)
HDL Cholesterol (mmol/l)	22	1.1 (0.7–2.8)	20	1.1 (0.8–3.5)	42	1.1 (0.7–3.5)
Total Cholesterol (mmol/l)	23	4.3 (2.1–7.5)	20	4.6 (1.0–6.5)	43	4.3 (1–7.5)
LDL Cholesterol (mmol/l)	19	2.5 (0.8–3.8)	18	3.2 (1.2–4.4)	37	3 (0.8–4.4)
Platelets (x10^9^/L)	23	224 (137–360)	21	224 (158–404)	44	224 (137–404)
ELF test	23	8.8 (6.5–11.8)	19	8.0 (6.4–10.3)	42	8.3 (6.4–11.8)

Median and (Range) AST, aspartame aminotransferase; ALT, alanine aminotransferase; ALP, alkaline phosphatase; Gamma GT, gamma glutamyl transferase; ELF, Enhanced liver fibrosis test; HDL, high density lipoprotein; LDL, low density lipoprotein; BP, blood pressure.

### Primary outcome—Change in fibrosis stage between baseline and week 96

A total of 32 (15 Losartan and 17 placebo) out of the 45 (71%) patients who were randomised had an end of study liver biopsy to assess fibrosis. Overall, one patient (6.7%) treated with Losartan was a treatment “responder” (lower fibrosis stage at follow up compared with baseline) and 4 patients (23.5%) in the placebo group were treatment “responders”. However, the small study size means that no firm conclusion on drug efficacy can be drawn from these results. Tables 2 and 3 summarises the change in fibrosis stage over the study period in the Losartan and placebo groups.

**Table 2:** Summary statistics for liver fibrosis assessed by the NASH CRN criteria [27] at baseline and 96 weeks by trial arm.

Variable	Losartan		Placebo	
Fibrosis stage	Baseline n = 15	96 weeks n = 15	Baseline n = 17	96 weeks n = 17
**0**	1 (6.7%)	1 (6.7%)	1 (5.9%)	1 (5.9%)
**1**	3 (20%)	2 (13.3%)	0 (1%)	2 (11.8%)
**2**	6 (40%)	7 (46.7%)	8 (47.1%)	6 (35.3%)
**3**	4 (26.7%)	4 (26.7%)	7 (41.2%)	6 (35.3%)
**4**	1 (6.7%)	1 (6.7%)	1 (5.9%)	2 (11.8%)
**Mean (sd)**	2.07 (1.03)	2.13 (0.99)	2.41 (0.87)	2.35 (1.06)
**Median (IQR)**	2 (1–3)	2 (2–3)	2 (2–3)	2 (2–3)

**Table 3:** Change in fibrosis stage from baseline to 96 weeks.

Variable	Losartan n = 15	Placebo n = 17
**Change in fibrosis stage from baseline to 96 weeks**	-1 n = 1 (6.7%) 0 n = 12 (80%) 1 n = 2 (13.3%)	-1 n = 4 (23.5%) 0 n = 10 (58.8%) 1 n = 3 (17.6%)
**Median change in fibrosis stage from baseline to 96 weeks (IQR)**	0 (0–0)	0 (0–0)

### Secondary outcomes

**Fig 3:**
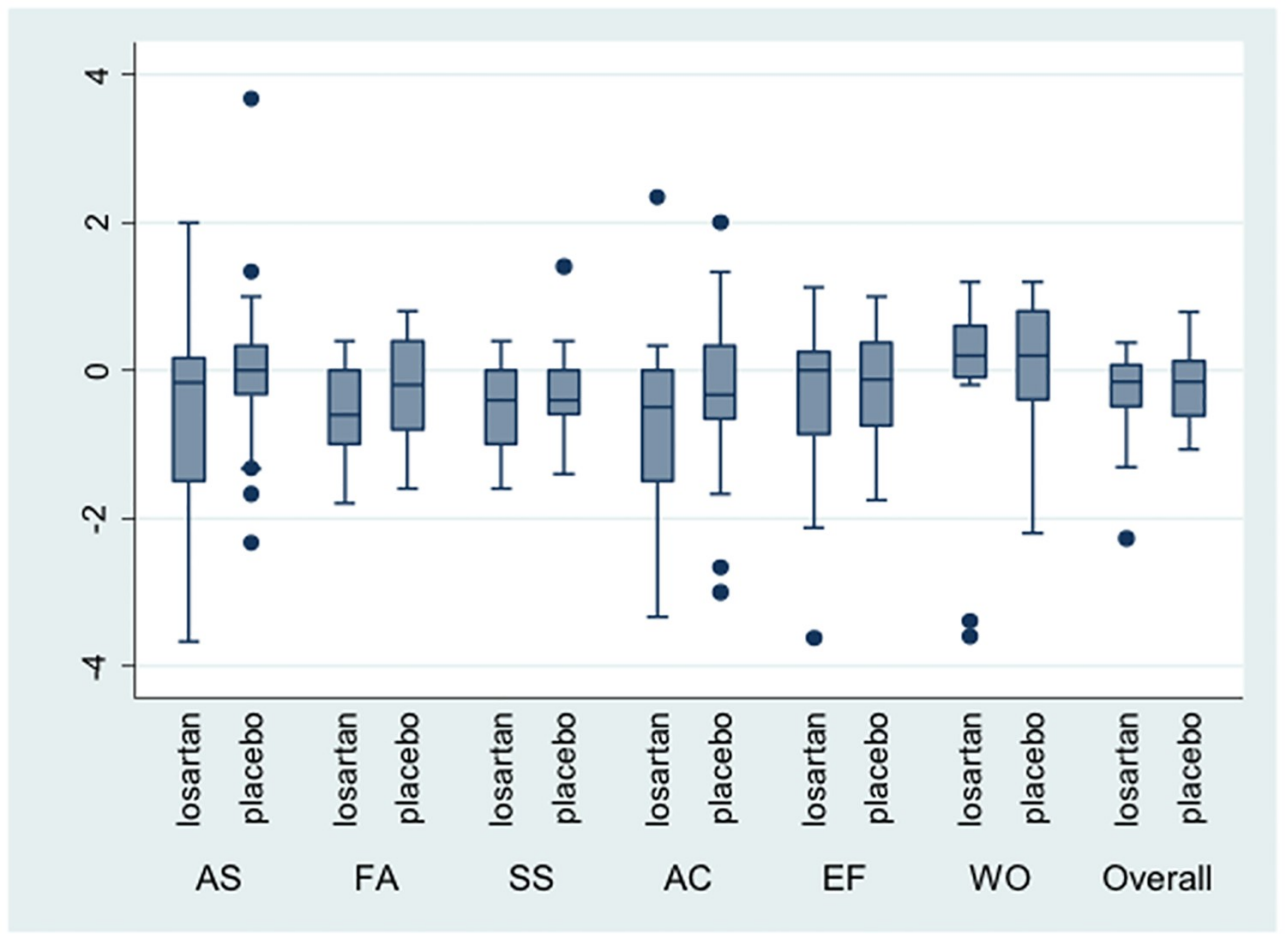
Quality of life as measured by the Chronic Liver Disease Questionnaire (CDLQ) during the study period (from baseline to end of treatment [week 96]). (AS = abdominal symptoms; FS = Fatigue symptoms; SS = systemic symptoms; AC = Activity; EF = Emotional functioning; WO = worry).

The change in biochemical and clinical features between baseline and end of treatment (96 weeks) is shown in Table 4 for the Losartan and placebo treated patients. All parameters were similar between baseline and end of treatment in both groups, but statistical testing was not preformed due to the small sample size. Overall, there was no change in NAS over the treatment period in patients treated with losartan (median change in NAS 0 [range -3 to 3]), whereas there was a reduction in NAS in the placebo treated patients (median change in NAS -1 [range -4 to 1]. Overall quality of life, as measured by the CLDQ, remained largely unchanged in both groups over the trial period (Fig 3).

**Table 4:** Clinical characteristics at baseline and 96 weeks in the losartan and placebo treated patients.

	Losartan				Placebo			
	Baseline (N = 24)		96 weeks (N = 18)		Baseline (N = 21)		96 weeks (N = 19)	
Variable	n	Median (range)	n	Median (range)	n	Median (range)	n	Median (range)
Weight (kg)	24	85.1 (74.2–121.0)	18	86.6 (73.5–113.9)	21	96.7 (61.6–132.5)	19	94.2 (60.3–129.7)
Waist circumference (cm)	24	105.9 (96.0–126.0)	18	108.0 (96.0–129.0)	21	111.4 (88.0–136.0)	19	112.5 (93.0–131.0)
AST (U/L)	21	35 (14–102)	17	31 (2–70)	18	46 (30–70)	16	36 (17–110)
ALT (U/L)	24	52.5 (21–136)	18	32.5 (7–80)	21	65 (33–135)	18	52 (18–133)
ALP (U/L)	24	89.5 (44–173)	18	89.5 (37–191)	21	72 (49–116)	17	79 (46–106)
Gamma GT (U/L)	24	70 (18–355)	18	55.5 (22–371)	21	62 (23–256)	17	48 (16–342)
ELF test	23	8.8 (6.5–11.8)	18	9.4 (7.9–11.0)	19	8.0 (6.4–10.3)	15	9.1 (7.3–10.8)
Creatinine (umol/L)	24	75.5 (48–105)	18	73.5 (43–106)	21	72 (5–97)	17	68 (52–94)
Triglyceride (mmol/L)	23	1.7 (0.9–7.9)	17	2 (0.8–6.5)	19	2.0 (0.4–4.4)	18	1.7 (0.9–4.4)
HDL Cholesterol (mmol/L)	22	1.1 (0.7–2.8)	16	1.0 (0.8–1.9)	20	1.1 (0.8–3.5)	16	1.1 (0.8–1.8)
Total Cholesterol (mmol/L)	23	4.3 (2.1–7.5)	16	4.0 (2.2–7.6)	20	4.6 (1.0–6.5)	18	4.4 (2.7–6.5)
LDL Cholesterol (mmol/L)	19	2.5 (0.8–3.8)	13	1.9 (0.5–4.2)	18	3.2 (1.2–4.4)	15	2.6 (1.0–3.8)
Glucose (mmol/L)	24	6.0 (4.4–17.1)	18	8.1 (4.8–19.7)	20	6.2 (3.6–15.9)	18	6.7 (4.3–15.8)
Systolic BP	24	133.5 (109–165)	18	129 (106–152)	21	127 (115–180)	19	125 (111–175)
Diastolic BP	24	78.5 (67–95)	18	74 (63–96)	21	81 (70–100)	19	80 (65–96)
Platelets (x10^9/L)	23	224 (137–360)	18	224 (135–362)	21	224 (158–404)	19	209 (155–349)

Median (range). AST, aspartame aminotransferase; ALT, alanine aminotransferase; ALP, alkaline phosphatase; Gamma GT, gamma glutamyl transferase; ELF, Enhanced liver fibrosis test; HDL, high density lipoprotein; LDL, low density lipoprotein; BP, blood pressure.

Due to the small sample size and the relatively few patients whose fibrosis stage changed over the study period, an analysis of the association between histological markers serum of fibrosis over the study period was not performed.

### Adverse Events (AEs)

Overall, there were 100 AEs in the Losartan treated patients and 101 in the placebo group (Table 5). There were 3 serious adverse events (SAEs) in patients who received Losartan, but all were thought not related to treatment (1 fracture metacarpal, 1 dislocation of metacarpal and 1 epistaxis). There was 1 SAE in the placebo group, though not related (1 fall and rib fracture).

**Table 5:** Reported adverse events in the study population (ITT analysis).

Related to treatment	Severity	Losartan n(%)	Placebo N(%)
**Not related**	**Mild**	43 (51.19%)	56 (76.71%)
	**Moderate**	34 (40.48%)	16 (21.92%)
	**Severe**	7 (8.33%)	0 (0%)
	**Missing**	0 (0%)	1 (1.37%)
	**Total**	**84**	**73**
**Possibly related**	**Mild**	9 (60%)	24 (92.31%)
	**Moderate**	6 (40%)	1 (3.85%)
	**Severe**	0 (0%)	1 (3.85%)
	**Missing**	0 (0%)	0 (0%)
	**Total**	**15**	**26**
**Probably related**	**Mild**	1 (100%)	0 (0%)
	**Moderate**	0 (0%)	0 (0%)
	**Severe**	0 (0%)	0 (0%)
	**Missing**	0 (0%)	0 (0%)
	**Total**	**1**	**0**
**Missing**	**Mild**	0 (0%)	2 (100%)
	**Moderate**	0 (0%)	0 (0%)
	**Severe**	0 (0%)	0 (0%)
	**Missing**	0 (0%)	0 (0%)
	**Total**	**0**	**2**
**Arm Total**		**100**	**101**

### Reasons for the inadequate recruitment

**Fig 4:**
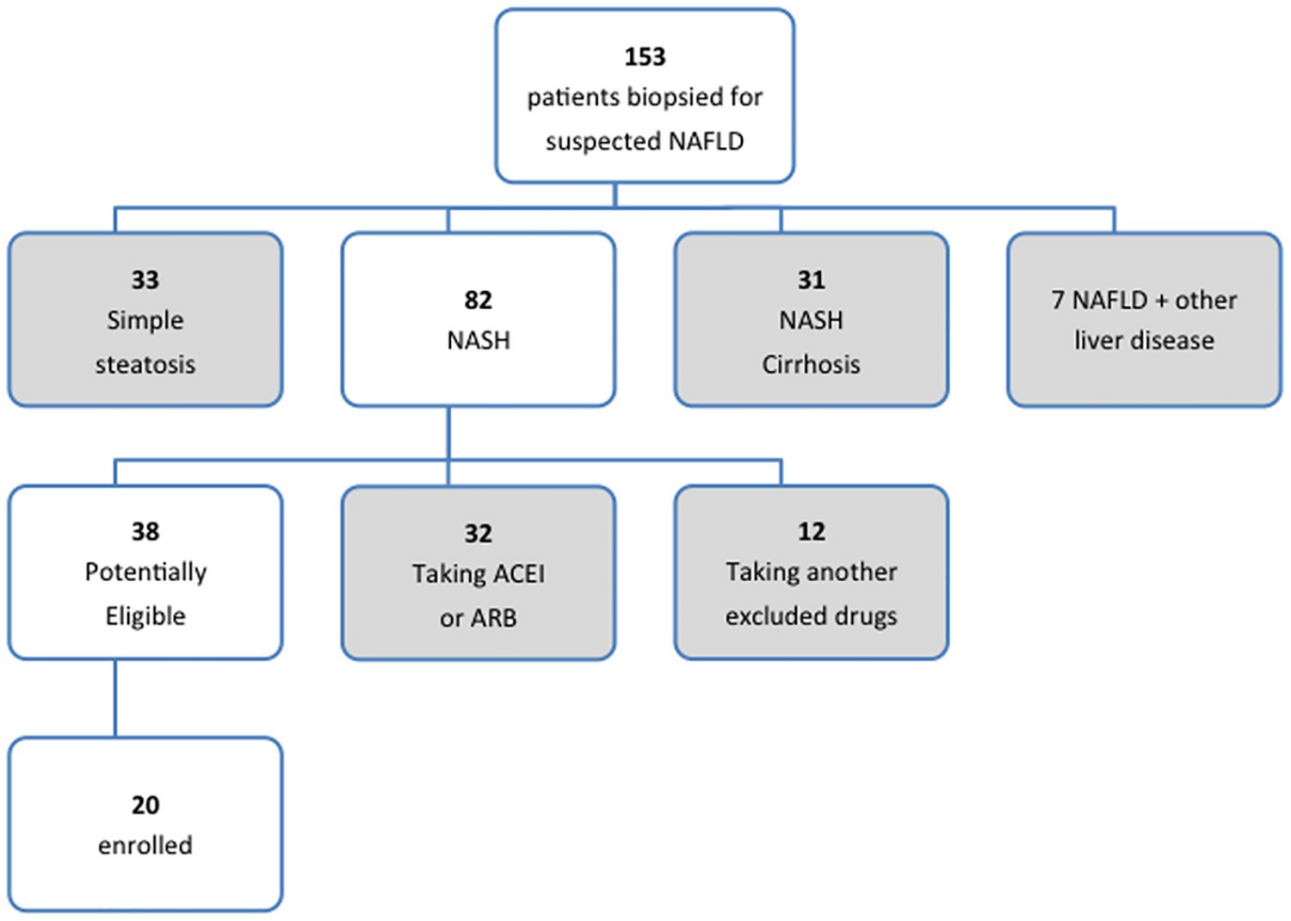
An overview of a detailed analysis of eligibility for all patients who had a liver biopsy to diagnose or stage NAFLD in Newcastle between Jan 2011 and Sept 2012 (21 months).

Overall, a total of 1130 patients were “pre-screened” in the sites for the study, and from this only 54 were formally screened for the study. The major reason for low recruitment rates appeared to be the widespread pre-existing use of ACEI or ARBs in NAFLD patients who were otherwise eligible for the study. A detailed analysis of eligibility for the study was conducted in patients from Newcastle and is shown in Fig 4. Overall, between Jan 2011 and Sept 2012 (21 months) 153 patients had a liver biopsy to diagnose or stage NAFLD. Of these, 82 (54%) had non-cirrhotic NASH, 38 (25%) were potentially eligible for the study and 20 (13%) were ultimately enrolled. Thirty-two (39%) of the patients with non-cirrhotic NASH were taking and ACEI or and ARB and 12 (15%) were taking other prohibited medication, making them ineligible for the study.

**Fig 5:**
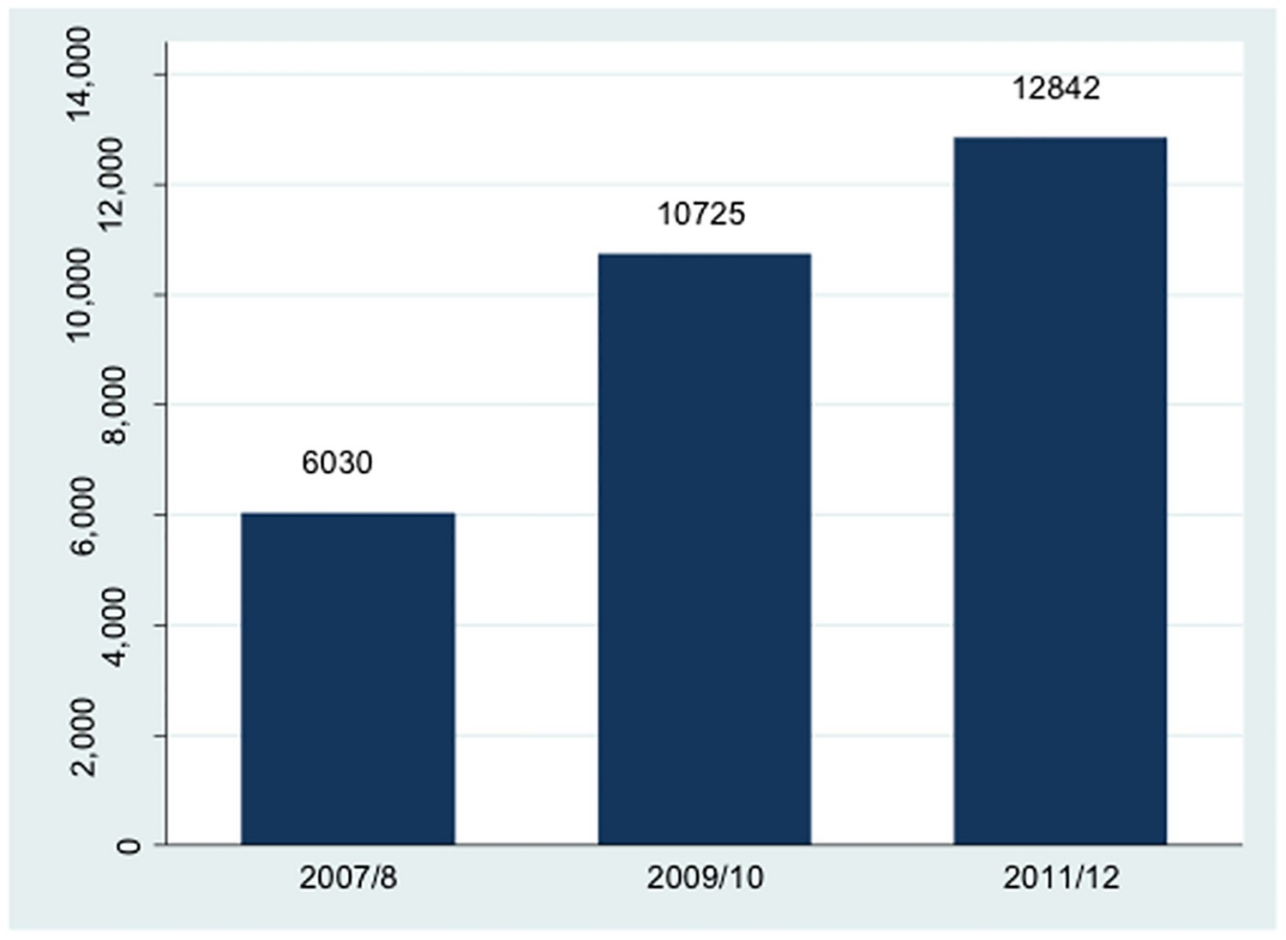
Number of patients prescribed Angiotensin Converting Enzyme Inhibitors (ACEI) or Angiotensin Receptor Blockers (ARB) in the Newcastle Primary care cohort (n = 149,097) from the time of conception (2007–8), to obtaining funding (2009), to recruitment beginning (2011).

Over the time from conception of the study (2007–8), to obtaining funding (2009), to recruitment beginning (2011) there was a change in the management of hypertension in the UK, with ACEI or ARBs being recommended first line by the National Institute of Health and Care Excellence (NICE) in August 2011 [35]. Fig 5 shows the increasing number of patients prescribed ACEI or ARBs in a Newcastle Primary care cohort, which includes prescribing data for 149,097 patients, between 2007 and 2012. There was a more than doubling of patients prescribed ACEI or ARBs in Newcastle from the time of conception of the study to recruitment commencing, which may have had an impact by reducing the pool of eligible patients for the study.

Other reasons cited by the participating centres for low recruitment rates was the increasing use of transient elastography to stage liver fibrosis in patients with NAFLD, which reduced the proportion of patients being diagnosed with NASH histologically. In addition, there were competing trials of other agents that were recruiting patients with NASH in the UK at that time.

## Discussion

Non-alcoholic fatty liver disease is now the most common chronic liver disease in many countries across the world [1], and is the second most common indication for liver transplantation in the USA, after hepatitis C [36]. Despite its high prevalence, there are currently no specific licensed therapies to treat NAFLD, and lifestyle modification and treatment of co-existent features of the metabolic syndrome are the mainstay of its management [14]. In NAFLD the most important long-term prognostic factor is stage of liver fibrosis, and patients with advanced (stage 3–4) fibrosis have increased rates of liver-related morbidity and mortality, as well as increased all-cause mortality [11–13]. Therefore, anti-fibrotic drugs are an attractive therapeutic option as they could have the potential to reverse liver fibrosis and improve long-term prognosis.

There is a wealth of data demonstrating the antifibrotic effects of ACEI and ARBs in experimental and animal models of liver fibrosis [20–23]. However, to date few studies have been conducted in humans, particularly in patients with NASH (see below) [24][25]. The original aim of the present study was to determine whether 96 weeks of treatment with losartan slowed, halted or reversed fibrosis in a sufficiently powered randomised controlled trial of patients with NASH. However, recruitment was slower than expected and the trial was halted early after only 45 patients were randomised, and as a result had insufficient power to answer this question. Only one of the 15 (6.7%) Losartan treated patients with paired biopsies was a “treatment responder” (less fibrosis on the follow up biopsy compared with the baseline biopsy) compared with 4 of the 17 (23.5%) patients in the placebo group, suggesting no benefit from Losartan in this small cohort.

A major reason for the inadequate recruitment to the study was the increasing use of ACEI or ARBs in the UK, particularly with the 2011 National Institute of health and Care Excellence (NICE) guidelines recommending ACEI or ARBs first line for hypertension [35]. This was not predicted during the conception of the study. Over the period from conception of the study (2007/8) to the trial being conducted (2011/12), data from the Newcastle Primary care research cohort (n = 147,097) suggested that there was a more than 2-fold increase in the number of patients were prescribed ACEIs and ARBs in the Newcastle region, which supports this hypothesis. Moreover, we conducted a detailed examination of eligibility for the study in all the patients who had a liver biopsy for suspected NAFLD in Newcastle over the recruitment period and found that 39% of the patients with non-cirrhotic NASH (potentially eligible) were already taking an ACEI or ARB. ACEI and ARBs are also widely prescribed for hypertension in other countries. In one study from the USA 186 of 290 (64%) hypertensive patients with NASH were treated with ACEI or ARBs. [37]. ACEI and ARB are also widely prescribed in for reno-protection in individuals with diabetes. Conducting future trials of these drugs in adults with NASH is likely to be difficult as many of the potential recruits will already be prescribed ACEI or ARBs. Conducting a trial of ACEI or ARBs in children with NASH may be an option to determine their efficacy, particularly as their long term safety has been well demonstrated.

As well as the low recruitment rate in this study, the dropout rate was higher than expected (29%). The requirement for a follow up liver biopsy is likely to be a factor contributing to this high rate. Another potential contributing factor is that when the study participants were advised of the change in trial protocol that they may have felt that is was not worth continuing in the study.

The primary outcome measure for this study was the change in fibrosis stage between baseline and 96 weeks. Overall, including patients from both groups, there was no change in fibrosis stage over the study period, with 5 patients having improvement by 1 fibrosis stage, 5 having progression of 1 fibrosis stage and the others remaining stable. Our observed rate of fibrosis progression was less than had been expected based on available natural history data at the time. Since this study was designed, other studies have more clearly defined the natural history of fibrosis in NAFLD. These show that overall approximately 40% of patients have fibrosis progression and 20% will have fibrosis regression over longer follow up periods (median follow up 3.7–6.6 years) [8, 9] In general, fibrosis progression is slow in NASH with patients progressing by one fibrosis stage every 7.1 years [6]. It is likely that studies may need to be conducted over longer periods, such as 5 years, to see benefit from some anti-fibrotics.

To date, most trials have used the NASH CRN system as the endpoint for assessment of fibrosis, which stages fibrosis semi-quantitatively from 0 to 4 [27]. However, this method is relatively insensitive for subtle changes in fibrosis and has the potential to miss minor changes in fibrosis that could be clinically significant over the longer term. Assessment of collagen proportionate area using image analysis of liver biopsy specimens might offer a more sensitive method to assess change in liver fibrosis in future studies of antifibrotics [38]. There is also need for accurate biomarkers that can give an early indication of an anti-fibrotic effect, analogous to the “rapid viral response” in patients with chronic hepatitis C treated with interferon and ribavirin [39]. One of the secondary outcomes of this study was to determine whether serum fibrosis biomarkers could predict changes in fibrosis over the study period. However, this analysis was not conducted due to the small sample size.

A few other studies have suggested benefit from treatment with ARBs in patients with NASH. Firstly, Yokohama et al showed that 4 of 7 hypertensive patients treated with Losartan for 48 weeks had improvement in liver fibrosis [24]. Subsequently, a larger randomized trial comparing Telmisartan with Valsartan for 20 months in 54 hypertensive patients with NASH found a significant reduction in fibrosis stage and NAS in the patients treated with Telimsartan, but no significant change in these parameters in the Valsartan treated patients [25]. There were significant improvements in steatosis, serum transaminases and insulin sensitivity in both groups over the study period. Further support for the potential anti-fibrotic effect of drugs blocking renin-angiotensin system (RAS) comes from a cross sectional study of 290 hypertensive patients with NASH, which found that patients treated with ACEI or ARBs had less advanced fibrosis than patients treated with other anti-hypertensives [37]. However, in contrast, a well-conducted randomized open label trial from the USA found that Losartan when combined with rosiglitazone was no more effective than rosiglitazone alone or rosiglitazone combined with metformin [26]. It therefore remains unknown whether ARBs have an anti-fibrotic effect in patients with NASH.

Experimental studies have suggested a number of mechanisms whereby drugs that block the RAS could have a beneficial effect in patients with NASH and other liver diseases (reviewed in [20, 40]). Overall, in models of liver injury, there is upregulation of RAS activity in areas of active fibrosis [41]. Angiotensin II (Ang II), the physiologically active product of the RAS, activates hepatic stellate cells promoting their differentiation into HMS and inducing a fibrogenic state. HMS themselves produce Ang II, which activates the angiotensin 1 receptor (AT1R) and promotes the survival of the HMS by upregulating anti-apoptosis genes via NF-κB activation. [18, 42] Therefore, blocking this pathway with ACEI or ARBs could promote HMS apoptosis and lead to regression of fibrosis. As well as being potentially anti-fibrotic, drugs that block the RAS may also have beneficial metabolic effects in patients with NAFLD. Previous studies have shown that activation of the RAS may impair insulin signalling, contributing to insulin resistance, which is a key pathophysiological mechanism in NAFLD [43]. Interestingly treatment with ACEI or ARBs can improve insulin sensitivity, and a large meta-analysis found there was a 20% reduction in new onset diabetes in patients treated with ACEI or ARBs [44]. This improvement in insulin sensitivity could have a secondary effect of improving steatosis and steatohepatitis [25].

The present study also highlights that it can take significant amount of time from submission of an application for grant funding to support an investigator-led study, before funding is awarded and enrolment commences. During this time, routine clinical practice may change, rendering the study moot or hampering the ability to recruit patients into the study. For future studies we therefore recommend that, when there is a delay of more than 6-months between submission of the initial funding application and the trial commencing, feasibility is reassessed and an initial pilot study is conducted to establish recruitment feasibility prior to expanding the trial to full recruitment.

In conclusion, the aim of this double-blind randomised-controlled trial was to determine whether treatment with Losartan had an anti-fibrotic effect in patients with NASH. However, the trial under recruited due to the widespread use of ACEI and ARBs in the study population and as a result the study was unable to determine whether losartan has anti-fibrotic effects in the liver. Given the widespread use of ACEI and ARBs for hypertension in patients with NAFLD, conducting a definitive placebo controlled trial of these drugs will be a challenge. As ARBs are effective antihypertensive and may also improve insulin sensitivity, they seem a good choice to treat hypertension in patients with NAFLD, even if an anti-fibrotic effect is not proven.

## Supporting information

S1 File FELINE trial protocol. (DOC)

S2 File CONSORT checklist. (DOC)

S3 File FELINE trial end of trial report. (PDF)
