# Supplementary material for: A randomised controlled trial of losartan as an anti-fibrotic agent in non-alcoholic steatohepatitis
Source: PLoS One. 2017 Apr 18;12(4):e0175717. doi: 10.1371/journal.pone.0175717 (PMC5395178; doi:10.1371/journal.pone.0175717)
Supplement: S1 File — (DOC) [file pone.0175717.s001.doc]

#

# A randomised controlled pilot trial of Losartan as an anti-fibrotic agent in non-alcoholic steatohepatitis

# (anti-Fibrotic Effects of Losartan In NASH Evaluation study - FELINE)

EudraCT Number: 2009-015166-62

ISRCTN Number: 57849521

REC Reference: 10/H0904/8

Protocol ID: BH134439/PD0117

Protocol Version 10.0 28 June 2013

Funded by: Newcastle NIHR Biomedical Research Centre

Grant reference: BH134439/PD0117

Sponsored by: The Newcastle Upon Tyne Hospitals NHS Foundation

Trust

Logo:
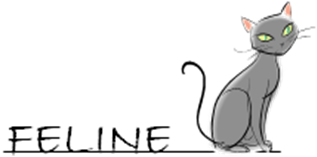


**1. Protocol Contacts**

**Chief Investigator**

Professor Christopher Paul Day

Institute of Cellular Medicine, Newcastle University, Faculty Office, Medical School, Newcastle upon Tyne, NE2 4HH, UK

+44 (0)191 2227003

[chris.day@ncl.ac.uk](mailto:c.p.day@ncl.ac.uk)

**Co-Investigators**

Professor Derek Mann

Institute of Cellular Medicine, Newcastle University, 4^th^ Floor, William Leech Building, Medical School, Newcastle upon Tyne, NE2 4HH, UK

+44(0)191 2223851

[derek.mann@ncl.ac.uk](mailto:derek.mann@ncl.ac.uk)

Dr. Stephen Frederick Stewart

Liver Centre

Mater Misericordiae Hospital

55 Eccles Street

Dublin 7

+353 1 803 2048

[sstewart@mater.ie](mailto:sstewart@mater.ie)

Dr Donald Stuart McPherson

Department of Gastroenterology

Freeman Hospital

High Heaton

Newcastle upon Tyne

NE7 7DN

+44(0)191 2232128

[stuart.mcpherson@nuth.nhs.uk](mailto:stuart.mcpherson@nuth.nhs.uk)

Dr Quentin Anstee

The Institute of Cellular Medicine

The Medical School

3rd Floor, William Leech Building

Newcastle University

Framlington Place

Newcastle-Upon-Tyne

NE2 4HH

+44 (0) 191 2227012

[quentin.anstee@newcastle.ac.uk](mailto:quentin.anstee@newcastle.ac.uk)

Professor Elaine McColl

Newcastle Clinical Trials Unit, Institute of Health and Society, Newcastle University, 4^th^ Floor William Leech Building, Medical School, Framlington Place, Newcastle upon Tyne, NE2 4HH, UK

+44(0)191 2227260

[e.mccoll@ncl.ac.uk](mailto:e.mccoll@ncl.ac.uk)

**Statistician**

Dr. Ian Nicholas Steen

Institute of Health and Society, Newcastle University, Baddiley-Clark Building, Richardson Road, Newcastle upon Tyne, NE2 4AX, UK

+44(0)191 2226488

[nick.steen@ncl.ac.uk](mailto:nick.steen@ncl.ac.uk)

**Senior Trial Manager**

Ms Jennifer Wilkinson

Newcastle Clinical Trials Unit, Institute of Health and Society, Newcastle University, 4^th^ Floor William Leech Building, Medical School, Framlington Place, Newcastle upon Tyne, NE2 4HH, UK

+44(0)191 222 7968

[jennifer.wilkinson@ncl.ac.uk](mailto:jennifer.wilkinson@ncl.ac.uk)

**Trial Manager**

Ms Jane Barnes

Newcastle Clinical Trials Unit, Institute of Health and Society, Newcastle University, 4^th^ Floor William Leech Building, Medical School, Framlington Place, Newcastle upon Tyne, NE2 4HH, UK

+44(0)191 222 7249

[jane.barnes@ncl.ac.uk](mailto:jane.barnes@ncl.ac.uk)

**Central Labs / Central Assessment contacts**

Professor A. D. Burt

Department of Cellular Pathology

Newcastle Hospitals NHS Foundation Trust

Royal Victoria Infirmary,

NEWCASTLE UPON TYNE

NE1 4LP

+44(0)191 2820700

[a.d.burt@ncl.ac.uk](mailto:a.d.burt@ncl.ac.uk)

**Trial Steering Committee**

(1) Dr Graeme Alexander

Consultant Hepatologist

Addenbrooke’s Hospital

Cambridge University Hospitals NHS Foundation Trust

CAMBRIDGE

CB2 0QQ

+44 (0)1223 586614

[gja1000@doctors.org.uk](mailto:gja1000@doctors.org.uk)

(2) Professor John N Matthews

Professor of Medical Statistics

School of Mathematics and Statistics

Newcastle University

Herschel Building

Newcastle upon Tyne

NE1 7RU, UK

+44 191 222 7259

[j.n.s.matthews@ncl.ac.uk](mailto:j.n.s.matthews@ncl.ac.uk)

(3) Joan Bedlington

10 Birchwood Close

Beamish

Stanley

Co. Durham

DH9 0SN

+44 191 3701713

[livernorth@hotmail.com](mailto:livernorth@hotmail.com)

(4) Elsie Clayton

5 Dunelm Drive

Dairy Lane Estate

Houghton-le-Spring

Tyne & Wear

DH4 5QG

+44 191 5844382

[wesnelsie@hlespring.freeserve.co.uk](mailto:wesnelsie@hlespring.freeserve.co.uk)

**Data Monitoring & Ethics Committee**

(1) **Chairman**

Professor John O’Grady

Consultant Hepatologist

Institute of Liver Studies

Kings College Hospital

Denmark Hill

London

SE5 9RS, UK

+44 (0)203 2999000

john.o’grady@kcl.ac.uk

(2) Dr Jane Collier

Oxford Radcliffe Hospitals NHS Trust

John Radcliffe Hospitals

Headley Way

Headington

Oxford

OX3 9DU, UK

+44 (0)1865 741166

[jane.collier@orh.nhs.uk](mailto:jane.collier@orh.nhs.uk)

(3) **Statistician**

Dr Walter Gregory

Cancer Division Director and Principal Statistician

Cancer Trials Research Unit

University of Leeds

Clinical Trials Research House

71-75 Clarendon Road

LEEDS, LS2 9PH, UK

+44 113 3431489

[w.m.gregory@leeds.ac.uk](mailto:w.m.gregory@leeds.ac.uk)

**Study Website address**

To be determined

**2. Protocol Signature Page**

## 2.1 PROTOCOL AUTHORISATION SIGNATORIES

Signature ……………………………… Date …………

**Professor Christopher Paul Day, Chief Investigator**

Signature ……………………………… Date …………

**Dr Ian Nicholas Steen, Statistician**

Signature ……………………………… Date …………

**Jennifer Wilkinson, Senior Trial Manager**

#### 2.2 Principal Investigator signature

I confirm that I have read and understood protocol version 10.0 dated 28 June 2013. I agree to comply with the study protocol, the principles of GCP, research governance, clinical trial regulations and appropriate reporting requirements.

Signature ……………………………… Date …………

Print Name ………………………………

Full Site Name ………………………………

**3. Contents Page**

**Contents Page**

Front Page

1. Protocol Contacts 2

2. Protocol Signature Page 6

3. Contents Page 7

4. Glossary of Abbreviations 8

5. Responsibilities 9

6. Protocol Summary 11

7. Background 12

8. Objectives 17

9. Study Design 20

10. Subject Population 24

11. Screening, Recruitment and Consent 26

12. Study Medication/Intervention Details 28

13. Randomisation 31

14. Blinding 31

15. Study Data 32

16. Statistical Considerations 40

17. Compliance and Withdrawal 42

18. Data Monitoring, Quality Control and Quality Assurance 43

19. Pharmacovigilance 45

20. Ethics and Regulatory Issues 50

21. Confidentiality 51

22. Insurance and Finance 52

23. Study Report/Publications 53

24. References 54

25. Appendices 55

**4. Glossary of Abbreviations**

| ***Abbreviation*** | ***Definition*** |
| --- | --- |
| *ACE* | *angiotensin converting enzyme* |
| *ALD* | *Alcoholic liver disease* |
| *ARB* | *angiotensin receptor blocker* |
| *AST* | *Aspartate transaminase* |
| *BLDCIG* | *British Liver Disease Clinical Interest Group* |
| *BRC* | *Biomedical Research Centre* |
| *CI* | *Chief investigator* |
| *CLDQ* | *Chronic Liver Disease Questionnaire* |
| *CRF* | *Case Report Form* |
| *CTA* | *Clinical trial authorisation* |
| *DILDIN* | *Drug Induced Liver Injury Network* |
| *ELF* | *enhanced liver fibrosis test* |
| *EME* | *Efficacy, Mechanism and Evaluation* |
| *GCP* | *Good Clinical Practice* |
| *GGT* | *Gamma Glutamyltransferase* |
| *GSK* | *GlaxoSmithKline* |
| *IMP* | *Investigational medicinal product* |
| *IHC* | *Immunohistochemistry* |
| *HCV* | *Hepatitis C Virus* |
| *HMS* | *hepatatic myofibroblasts* |
| *NAFLD* | *non-alcoholic fatty liver disease* |
| *NAS* | *NAFLD Activity Score* |
| *NASH* | *non-alcoholic steatohepatitis* |
| *NIHR* | *National Institute for Health Research* |
| *NSAIDs* | *Non-steroidal anti-inflammatory drugs* |
| *PCNA* | *Proliferating Cell Nuclear Antigen,* |
| *PI* | *Principal investigator* |
| PIC | *Patient Identification Centre* |
| Qrt-PCR | *Quantitative Real Time Polymerase Chain Reaction* |
| *R&D* | *Research and Development* |
| *REC* | *Research ethics committee* |
| *SAE* | *Serious adverse event* |
| *SF-36* | *Short form-36 (questionnaire)* |
| *SmPC* | *Summary of major product characteristics* |
| *SOP* | *Standard Operating Procedure* |
| *SUSAR* | *Suspected unexpected serious adverse reaction* |
| *TUNEL* | *Terminal deoxynucleotidyl transferase dUTP nick end labelling* |

**5. Responsibilities**

## Sponsor: Newcastle upon Tyne Hospitals NHS Foundation Trust will act as the sponsor for this study. Day-to-day responsibility for sponsor level activities will be delegated to the Chief Investigator and the Newcastle Clinical Trials Unit

**Funder:** Newcastle NIHR Biomedical Research Centre and Unit, Biomedical Research Building, Newcastle University, Campus for Ageing and Vitality, Newcastle upon Tyne, NE4 5PL. Funder’s reference: BH134439/PD0117. Contact Dr Mark Jarvis, NIHR Biomedical Research Centre & Unit Manager, Telephone: +44 (0) 191 248 1147; Email: [mark.jarvis@ncl.ac.uk](mailto:mark.jarvis@ncl.ac.uk)

**Trial Management:** A Trial Management Group (TMG) will be appointed and will be responsible for overseeing the progress of the trial. The day-to-day management of the trial will be co-ordinated by the FELINE Trial Manager.

**Chief Investigator:** The Chief Investigator (Professor Christopher Paul Day) will have overall responsibility for the conduct of the study as a whole

**Principal Investigator:** The Principal Investigator will have overall responsibility for the conduct of the study at a particular trial site

**Trial Management:**

The following functions falling under the responsibility of the sponsor will be delegated to Professor Day as Chief Investigator:

- Application for authorisation and Ethics Committee Opinion (including CTA request, research ethics committee opinion, R&D application, including site specific assessment & local approval procedures, notification of protocol amendments and end of trial ).
- Good Clinical Practice and Trial Conduct (including GCP arrangements, management of IMP, data monitoring, emergency & safety procedures).
- Pharmacovigilance (including defining & recording adverse events/reactions, reporting SUSARs, notifying investigators of SUSARs, ensuring that SAEs are reviewed by an appropriate committee for safety monitoring, annual listings & safety report).
- Administration of the study budget.

**Trial Conduct at Each Site:**

Principal Investigator responsibilities:

- Study conduct and the welfare of study subjects.
- Familiarity with the use of the IMP as described in the product information, appropriate storage, administration according to the protocol, and drug accountability. Ensuring that IMP is not used for any purposes other than the conduct of the study.
- Compliance with the protocol, documentation of any protocol deviations and reporting of all serious adverse events.
- Screening and recruitment of subjects.
- Ensuring that all trial-related medical decisions are made by a qualified physician, who is an investigator or co-investigator for the trial.
- Provision of adequate medical care in the event of an adverse event.
- Obtaining relevant local approvals and abide by the policies of Research Governance.
- Compliance with the Principles of GCP, Research Governance Framework and any national legislation implementing the EU Clinical Trials Directive (2001/20/EC) and subsequent amendments.
- Ensuring no participant is recruited into the study until all relevant regulatory permissions and approvals have been obtained.
- Obtaining written informed consent from participants prior to any study specific procedures.
- The PI should be qualified by education, training and experience to assume responsibility for the proper conduct of the trial. The Principal Investigator (PI) shall provide a current, signed and dated curriculum vitae as evidence for the Trial Master File
- Ensure that Study Site team members are appropriately qualified by education, training and experience to undertake the conduct of the study
- Availability for Investigator meetings, monitoring visits and in the case of an audit.
- Maintenance of study documentation and compliance with reporting requests
  - Maintaining an Investigator Site File, including copies of study approval, list of subjects and their signed informed consent forms.
  - Documenting appropriate delegation of tasks to study personnel eg. Pharmacist, Research Nurse, Investigator(s).
  - Ensuring data collected are accurate & complete.
  - Providing updates on the progress of the trial.
  - Ensuring subject confidentiality is maintained during the project and archival period.
  - Ensuring archival of study documentation for a minimum of 15 years following the end of the study, unless local arrangements require a longer period.

**6. Protocol Summary**

Short title: FELINE

Protocol version: 10.0

Protocol date: 28 June 2013

Chief Investigator: Professor Christopher Paul Day

Sponsor: The Newcastle upon Tyne Hospitals NHS Foundation Trust

Funder: Newcastle NIHR Biomedical Research Centre Funding

Study design: Randomised, double-blind, placebo-controlled pilot study

Study Intervention: Losartan versus placebo (1:1 ratio)

Primary objective: To determine whether Losartan is effective at slowing down, halting or reversing liver fibrosis in patients with NASH over a two year period.

Secondary objectives: To determine whether Losartan can prevent clinical deterioration in NASH over a two year period.

To determine the association between serum, radiological and histological markers of fibrosis in patients with NASH over a two year period.

###### Study population/size: 214 adult (aged ≥18 years) patients (107 per group)

with steatohepatitis and fibrosis, resulting from

non-alcoholic fatty liver disease.

Study duration: 42 months

**7. Background**

**7.1 Study Rationale**

The United Kingdom is experiencing a dramatic increase in the number of patients presenting with liver disease, with as many as 1 in 10 of the population having some form of liver disorder (British Liver Trust 2006). Liver disease is currently the 5th highest cause of mortality in the UK population and, in contrast to the "top 4", mortality from liver disease is rising. Recent estimates have suggested a doubling of liver disease mortality over the next decade. Liver disease is also associated with considerable morbidity, ranging from impaired quality of life through to the classical complications of advanced liver disease including liver failure, variceal bleeding, ascites, encephalopathy and hepatocellular carcinoma. These conditions, all the result of progressive liver fibrosis, are the complications which dominate the wards of Liver Units throughout the country.

One of the major reasons for the increase in mortality from liver disease is the well publicised "obesity epidemic" pre-disposing to non-alcoholic fatty liver disease (NAFLD), ranging from simple steatosis through non-alcoholic steatohepatitis (NASH) to cirrhosis. We have recently reported that, in addition to the classical complications of advanced liver disease, patients with NAFLD suffer from severe fatigue associated with inactivity and daytime somnolence.[1] (Newton et al. Gut. 2008 June: 57(6): 807-13). Treatment for patients with NAFLD is currently limited to lifestyle change (weight loss and exercise) which is, at best, only partially effective. For patients with advanced disease, liver transplantation is the only effective option: however, it is expensive, limited to certain groups and hampered by problems related to donor availability.

Currently there are no available therapies aimed at the fibrosis component of the disease, in spite of this being the main feature associated with liver-related mortality. An effective anti-fibrotic agent would have consequences for the complications of cirrhosis and, ultimately, mortality. Given that cirrhosis is the end-point for nearly every chronic liver disease, anti-fibrotic agents would have broad utility for hepatologists.

The mechanisms underlying the variable rates of fibrosis progression in NAFLD are unclear. Natural history data to date suggest that over a two to four year period, 20-30% of patients will undergo fibrosis regression, 25-40% will undergo progression and 35-50% will remain stable. Research into these mechanisms may shed light on this variability and will help in determining prognosis and targeting future therapies.

**7.2 Patient Population**

Study population will be patients (≥ 18 years) with steatohepatitis and fibrosis, resulting from non-alcoholic fatty liver disease. Steatohepatitis will be defined as those patients with steatosis, liver cell injury and lobular inflammation. Fibrosis can be Kleiner stage 1-3. These parameters should define a patient cohort at risk of fibrosis progression that has the capacity for fibrosis regression.

**7.3** **Previous Studies**

The proof-of-concept data [2] Yokohama et al. *Therapeutic efficacy of an angiotensin* *II receptor antagonist in patients with non-alcoholic steatohepatitis.* Hepatology 2004 40(5): 1222-5. [3] Colmenero et al, *Losartan reduces the expression of profibrogenic genes and inflammation in patients with chronic hepatitis C* Hepatology 2007 46(4) Suppl. 1, 716A) would lead us to believe that Losartan (an angiotensin receptor blocker (ARB)) will be an effective anti-fibrotic agent in NASH patients and, given the lack of therapies available in this condition, this would lead to a substantial health gain.

Natural history data to date suggest that over a two to four year period, 20-30% of patients with NASH undergo fibrosis regression, 25-40% will undergo progression and 35-50% will remain stable.[4](Lindor et al *Ursodeoxycholic Acid for Treatment of Nonalcoholic Steatohepatitis: Results of a Randomized Trial Hepatology*, 2004, 39(3), pp770-8; Adams et al [5]*The histological course of nonalcoholic fatty liver disease: a longitudinal study of 103 patients with sequential liver biopsies* Journal of Hepatology 2005, 42, pp132–138) The progression of fibrosis to cirrhosis is the most important factor that determines patient mortality in NASH.[6](Adams et al *The Natural History of Nonalcoholic Fatty Liver Disease: A Population-Based Cohort Study*, Gastroenterology 2005, 129(1) pp113-121) To date, no pharmacotherapeutic agents are prescribed with the aim of inhibiting or reversing fibrosis. Recently, however, evidence about the nature of fibrosis in liver disease and the mechanisms for fibrosis progression have led to clinical studies which make fibrogenesis a valid therapeutic target.

Fibrosis is now seen as a highly dynamic process with potential for regression and progression. Clinical and experimental studies provide strong evidence that even advanced fibrosis can undergo regression if the underlying cause of liver disease is treated [7, 8](Hepatology 2006; 43 [Suppl 1]:S82-8; International Journal of Biochemistry & Cell Biology 2007 39: 695-714). However, not all patients undergo spontaneous fibrosis regression and in those who are responsive the process is slow and incomplete. Moreover, regression will not occur in the vast majority of patients for whom we are unable to treat the underlying cause of disease.

The data supporting a clinical trial of an ARB, incorporating basic science studies investigating the mechanisms of fibrosis in NASH, are described below.

**7.3.1 NF-κB as a regulator of liver fibrosis**

The Mann laboratory has investigated mechanisms by which liver fibrosis can undergo regression. Apoptosis is a naturally occurring process by which cells effectively commit suicide either in response to a death signal or due to the loss of survival signals. The Mann laboratory was involved in early work identifying apoptosis as a mechanism that clears hepatic myofibroblasts (HMS) and enables resolution of hepatic wound-healing [9](Wright MC et al, Gastroenterology, 2001 Sep;121(3):685-98). Most significantly, they identified the nuclear transcription factor NF-κB as a regulator of the survival and persistence of HMS. Specific inhibition of NF-κB on its own is sufficient to induce apoptosis of human HMS [10](Watson M et al, J Hepatol 2008 Apr;48(4):589-97). This was an important discovery since human HMSs are particularly resistant to apoptosis. Furthermore, the Mann laboratory was responsible for the seminal discovery that, when rats with liver fibrosis are treated with inhibitors of NF-κB, the fibrotic tissue is rapidly remodelled, with clear evidence for rapid regression of scars [11](Oakley F et al, Gastroenterology 2005 Jan;128(1):108-20). These exciting studies established a foundation for the pilot studies that led to the current proposal.

**7.3.2 A fibrogenic signaling pathway that promotes survival of HMS**


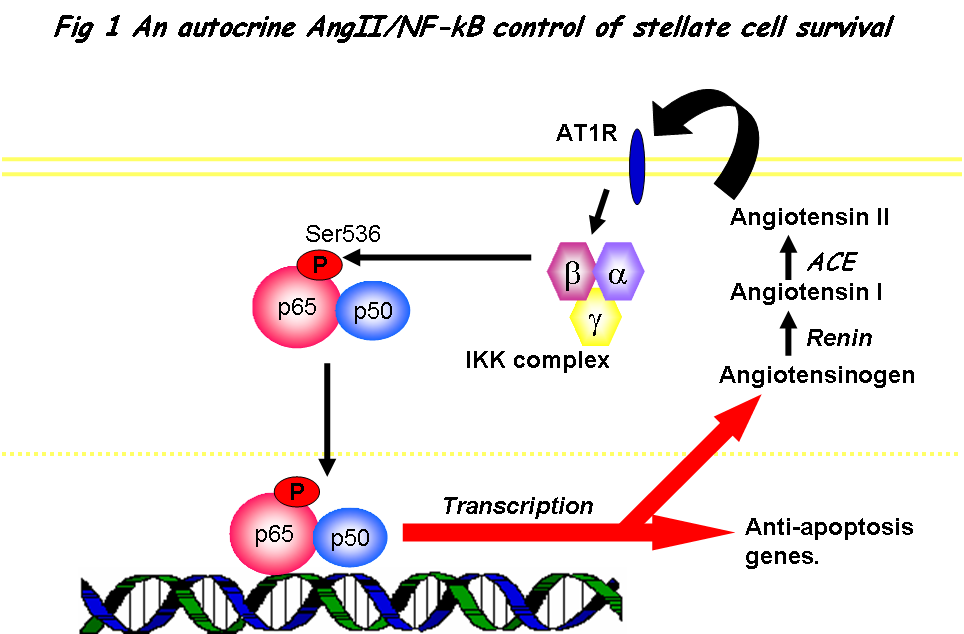
Recent work by the Mann laboratory has addressed the question of why NF-κB is constitutively active in HMS. In the process of answering this question they have discovered a signalling pathway that promotes fibrosis and which can be therapeutically targeted with drugs that are already known to be very well tolerated in man. Earlier studies by Bataller and colleagues described a local renin-angiotensin system in HMS which is a powerful stimulator of fibrosis [12, 13](Bataller R et al, Gastroenterology. 2003 Jul;125(1):117-25; Bataller R et al, Hepatology. 2005 May;41(5):1046-55). HMSs express angiotensinogen and the enzymes required to process it into angiotensin II which is continuously released by the cell. Furthermore, HMS express the ATI receptor which in response to angiotensin II stimulates fibrogenic characteristics of HMS including collagen synthesis. The Mann laboratory has discovered that angiotensin II stimulation of AT1 activates the IKappaKinase (IKK) complex, and subsequent phosphorylation of NF-κB on its RelA subunit at a specific residue, Ser536. Phosphorylation of RelA-Ser536 (RelA-pSer536) is required for NF-κB to enter the nucleus and drive transcription of cell survival genes (Fig 1). Human HMSs have constitutive nuclear RelA-pSer536 which is almost completely lost when cells are treated with ACE inhibitors or AT1 blockers such as Losartan. Such treatments are also associated with elevated rates of HMS apoptosis and, in animal models, regression of fibrosis. [10] (Watson, M.R. et al Hepatology 2008 48: 4 589-97) It was also established that expression of angiotensinogen is regulated by NF-κB. When taken together these observations provide evidence for an autocrine positive feedback pathway that enables HMSs to avoid apoptosis (fig 1).

**7.3.3 Human Studies with Angiotensin Receptor Blockers**

With respect to human studies, a small study in seven hypertensive patients with NAFLD showed a reduction in serum markers of fibrosis (Hyaluronic acid, procollagen III peptide and TGF-beta) after 48 weeks treatment with the ARB, Losartan [2](Yokohama et al Hepatology 2004;40:1222-5), Further 'proof-of-concept' evidence supporting the anti-fibrotic effect of Losartan comes from recent studies by collaborators in Barcelona demonstrating the ability of Losartan to promote regression of advanced fibrosis in transplant patients with recurring HCV disease [3](Colmenero et al Hepatology, Vol 46, No. 4, Suppl. 1, 2007 716A) .

**7.3.4 RelA-pSer536 as a predictive marker for fibrosis that is susceptible to regression in response to treatment with Losartan**


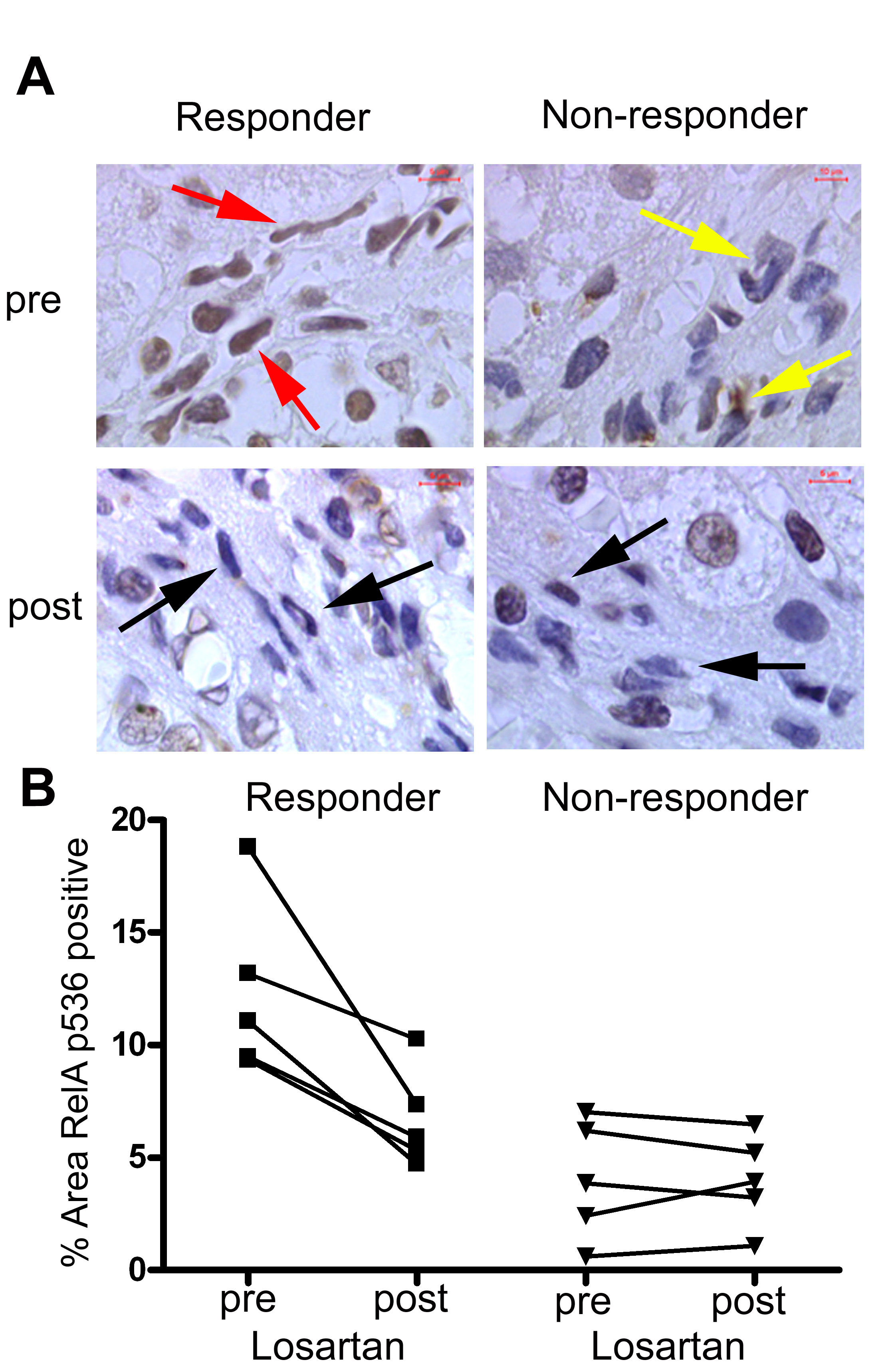
Our collaborator, Bataller (Barcelona) carried out a proof-of-principle trial in which 14 patients with fibrosis resulting from chronic HCV infection were treated with Losartan for 18 months. Patients underwent biopsy at the start and end of the trial and were assessed by pathology grading for regression of fibrosis in response to Losartan. Seven patients responded, with evidence for regression of fibrosis by one grade on the Metavir scale. The Mann laboratory obtained paired liver biopsy slides from responders and non-responders and determined levels of RelA-pSer536 by immunohistochemistry and morphometry. The livers of patients who underwent fibrosis regression had raised levels of RelA-pSer536 compared with non-responders (fig 2). Furthermore, with Losartan treatment, RelA-pSer536 expression in responders fell to the low levels measured in non-responders (which was unchanged between start and end points).

*Fig 2 – Hepatic RelA-pSer536 in HCV+ Losartan responders and non-responders (A)=Immunohistochemistry in liver sections (B) Counts of RelA-pSer536+ cells.*

From these observations we suggest that for a high proportion of patients, fibrosis is regulated at least in part by the angiotensin II/RelA-pSer536 pathway. We additionally propose the hypothesis that high levels of RelA-pSer536 in the pre-treatment biopsy may be predictive of liver disease patients who would benefit from therapy with either an ACE inhibitor or an AT1 receptor blocker.

**7.4 Current Treatment and Limitations**

NASH is a common condition and many hepatologists have a large cohort of patients with no specific therapy for this disease. There is now an urgent need to perform clinical trials of ARBs, such as Losartan, in patients with liver fibrosis, in order to halt the rising morbidity and mortality and to impact on the number of patients that progress to end-stage liver disease or transplant.

Although Losartan is not currently licensed as an anti-fibrotic, it is used widely in patients with hypertension, a common condition in patients with NASH. We know that it is cheap and safe, and we have no ethical concerns with prescribing it in this population. We feel that an angiotensin receptor blocker is more appropriate than an ACE inhibitor because the proof-of-concept work in humans was done with an ARB (specifically, Losartan). The dose and route of administration are those that have been proved to be safe, and those used in the pilot studies.

We believe that NAFLD is the obvious disease in which to evaluate this therapy as: (a) it is common and increasing in prevalence, (b) there is no alternative therapy of proven benefit, and (c) the only published proof-of-concept data are in this group of patients.

The main risks of ARB therapy are hypotension and renal failure. The population we will study is more likely to have hypertension as a component of the metabolic syndrome, so we would not expect hypotension to be a major concern. Nevertheless, monitoring will be aimed at early detection of both this and renal failure and, if necessary, the drug can be reduced or stopped.

The primary benefit to the patient is the slowing down of the progressive liver disease, and the reduced progression to cirrhosis, and end-stage liver disease with significant morbidity and mortality. This will also translate into a benefit to society, with fewer patients admitted to hospital with decompensation episodes (the clinical manifestations of end-stage liver disease such as encephalopathy, ascites and variceal bleeding), and fewer patients requiring transplantation, or dying from end-stage liver disease.

There are limitations to the study. The natural history of NASH is probably greater than 20 years from no fibrosis to cirrhosis in most patients. As such, a two-year period of treatment may be too short to determine whether the treatment prevents fibrosis progression. There are also concerns with regard to sampling error. A biopsy taken from one part of the liver may have a different fibrosis score to that taken from another part. This particular limitation should, however balance out between the treatment and placebo arms, and the statistical methods used should determine whether there is a treatment effect.

**8. Objectives**

**8.1** **Primary Clinical Objective**

The primary clinical objective is to determine whether Losartan (50 mg once a day vs. matched placebo, for 24 months) is effective at slowing down, halting or reversing liver fibrosis in patients with NASH. As cirrhosis occurs over many (10-20) years, the clinical assessments described below will attempt to measure a mean rate of change of fibrosis over a two-year period.

**8.2** **Secondary Clinical Objectives**

A secondary clinical objective is to determine whether Losartan can prevent clinical deterioration in NASH or impact on the quality of life. In addition, we intend to determine the association between serum, radiological and histological markers of fibrosis in patients with NASH, over a two-year period.

**8.3 Proposed Outcome Measures**

The primary outcome will be change in Kleiner fibrosis score [14][Kleiner DE et al Hepatology 2005] based on histological fibrosis stage (as judged by two independent blinded histopathologists, from liver biopsies), from pre-treatment to end-of-study (ie. two year follow-up).

Secondary outcomes will be changes in radiological (fibroscan) and serological (ELF and Fibromax) markers of fibrosis; both of which have been validated against the histological ‘gold standard’. These will be performed at trial entry, 48 weeks and end-of-study (96 weeks). A further secondary outcome is the change in NAFLD activity score (NAS) from baseline. This will be determined from trial entry and end-of-study liver biopsies. The NAS specifically includes only features of active injury that are potentially reversible, and is defined as the un-weighted sum of scores for steatosis (0-3), lobular inflammation (0-3) and hepatocyte ballooning (0-2). 'Responder rate' for placebo vs intervention will be compared. Responder rate is defined as end-of-treatment liver fibrosis stage less baseline liver fibrosis stage.

**8.4 Mechanistic Objectives**

**8.3.1. *To* *determine whether expression of RelA-pSer536 can be used to predict which patients with NAFLD will undergo regression of fibrosis in response to Losartan therapy***

This will involve quantifying the numbers of RelA-pSer536 positive HMSs in liver biopsy tissue, prior to and following treatment. We anticipate that those patients who respond to therapy will express high numbers of RelA-pSer536+ HMSs prior to therapy, which will then fall to the low levels observed in patients who fail to respond to therapy. By combining RelA-pSer536 IHC with stains for apoptosis and proliferation we will also discover whether expression of RelA-pSer536 is a determinant of HMS survival.

**8.3.2.** ***To determine the cellular mechanisms underlying response and lack of response to Losartan***

Our hypothesis is that Losartan stimulates regression of liver fibrosis by inhibiting phosphorylation of RelA at Ser536 which leads to the loss of nuclear NF-κB and expression of anti-apoptotic genes in HMs. Apoptosis of HMs then removes the cellular source of scar proteins and the collagenase inhibitor TIMP-1; this enables collagenases to remodel the fibrotic tissue. We suggest that the fibrotic tissue of patients who respond to Losartan is predominantly populated by HMs that rely on angiotensin II- regulated phosphorylation of RelA for fibrosis progression. By contrast, fibrotic tissue in patients who fail to respond do not predominantly employ this mechanism for HMS survival. We will test this hypothesis by employing qRT-PCR of biopsy material to determine changes in the expression of NF-κB-response genes, apoptosis regulators and fibrogenic genes resulting from Losartan therapy. Further, we will then compare these alterations in gene expression between responders and non-responders. We anticipate that we will identify alterations in gene expression that are selective for responders versus non-responders, which will provide important insights into the molecular events that favour Losartan-induced regression of fibrosis.

We will meet these aims by achieving the following objectives:

**8.3.2.1** ***Generate “in-house” anti-RelA-pSer536 monoclonal antibodies (Months 1-3)***

This is an important objective to ensure that we have a standardised supply of high quality antibodies for optimisation, validation and standardisation of the immunohistochemical assay.

**8.3.2.2** ***Antibody characterisation and IHC (Months 3-18)***

We will validate which monoclonal antibodies provide optimal recognition of the phosphorylated form of RelA using a combination of biochemical (Western blot) and cell biology (in situ immunoflourescence) methods prior to IHC. For IHC we will utilise archived paraffin-embedded blocks of human fibrotic liver to optimise antibodies for important components of the IHC technique such as primary antibody dilution; etc. The optimised protocol will be validated for detection of RelA-pSer536 in archived fibrotic liver from NAFLD patients.

**8.3.2.3 *Development of methods for the quantification and localisation of RelA-pSer536 in pre- and post-Losartan biopsy tissue (Months 12-24***

We will evaluate two protocols: (1) Blinded manual counting of RelA-pSer536 HMs (2) Image analysis/morphometric quantification of RelA-pSer536 staining. We will additionally assess protocols for determining HMS apoptosis (TUNEL) and proliferation (PCNA) and correlate these with levels of RelA-pSer536.

**8.3.2.4 *Development of qRT-PCR assays for validation of anti-fibrotic activities of Losartan using liver biopsy material (Months 1-24)***

To probe the mechanisms underlying response or lack of response to Losartan, we will develop a series of qRT-PCR assays for detection of NF-κB-target genes, poptosis regulatory genes and fibrogenic genes in biopsy specimens.

**8.3.2.5 *Application of RelA-pSer536 IHC and qRT-PCR biopsy assays on tissues from the Losartan trial (Months 24-36)***

The optimised and validated IHC and qRT-PCR assays will be employed to quantify RelA-pSer536 and gene expression in biopsy material obtained pre- and post-treatment from every patient in the trial.

**9. Study Design**

**9.1 Clinical Trial**

The planned intervention for this study is treatment with an angiotensin receptor blocker (Losartan) at a dose of 50 mg once a day vs. matched placebo for 24 months. The drug is very well tolerated and we do not predict a large loss to follow-up due to non-compliance.

In addition, in accordance with NASH Lifestyle Guidelines, all study participants (both arms) must follow the instructions from their treating physician with regard to diet, exercise and weight maintenance in compliance with standard care for NASH. The recommendation will be for 150 minutes of exercise/per week, combined with a reduction in intake of 500 kcal per day.

The trial element of this study comprises a parallel group, double-blind, randomised controlled trial of Losartan versus placebo, with analysis on the basis of intention-to-treat. Patients will be allocated in a 1:1 ratio, to Losartan 50 mg once a day or matched placebo, for 24 months.

Since diabetes is a known major risk factor for progression of fibrosis, we will stratify by presence of diabetes, to avoid imbalances between groups with respect to this potential confounder; we will also stratify by centre.

Randomisation will be via a centralised system to ensure concealment of treatment allocation. Patients, treating clinicians and those involved in outcome assessment will be blinded to treatment group by the over-encapsulation of Losartan, and the production of matched placebo capsules. .

We will record demographic and clinical characteristics carefully for all those screened to participate, and will note whether they are eligible according to our inclusion and exclusion criteria, and, if eligible, whether they agree to participate. This will allow us to demonstrate how representative the enrolled patients are of the underlying population of NASH patients; on the basis of previous studies, we estimate that 50% of those screened will prove eligible and will agree to participate. Even if the participant discontinues study medication prematurely, we will make every effort to obtain outcome data (prioritising primary outcome data) for all subjects over 24 months.

Based on previous studies in similar populations, we anticipate a loss to follow-up, in respect of the primary outcome (change in fibrosis score, based on biopsy-derived histological fibrosis stage, from baseline to 24 months follow-up), of approximately 20%. However, some patients who are unwilling to undergo follow-up biopsy may nonetheless be willing to complete monitoring of the non-invasive markers of fibrosis, and thus will provide data for analysis of secondary outcomes.

There are currently no pre-planned interim analyses or ‘stopping rules’ for the study as a whole. However, individual patients may be withdrawn from therapy if they develop a side-effect of the medication: hypotension, renal impairment or hyperkalaemia. If patients are withdrawn from therapy on the advice of treating clinicians, or at their own request, they will be asked to continue with follow-up assessments to 24 months. If they are unwilling to continue with follow-up visits for this length of time, they will be asked to consent to an ‘end of study’ visit, including liver biopsy and non-invasive tests of liver function and fibrosis at the point of withdrawal.

The end of study will be the last participant’s final study contact, at 24 months follow-up.

- 1. **Mechanistic studies**

***Generate an in-house affinity purified rabbit polyclonal and monoclonal anti-RelA-pSer536 (Months 1-3).***

Commercial antibodies are available for IHC detection of RelA-pSer536; however, they are of poor quality and suffer from batch-to-batch variability. We will generate our own antibodies to assure high quality control, which is critical for quantitative analysis of IHC staining of biopsy tissues. We will initially raise a rabbit polyclonal antisera to a 12-amino acid phosphopeptide corresponding to the Ser536 region of human RelA. Success with production of a polyclonal reagent will lead to subsequent immunisations in mice to generate monoclonal antibodies which have improved specificity for phosphorylated epitopes. However, in the unlikely case that we fail to generate a monoclonal antibody our approach should at the least generate large quantities of a polyclonal reagent. Production, purification of the antigen and conjugation to a carrier protein will be carried out commercially by Biogenes GmbH. We will also contract Biogenes GmbH to produce the anitibody, as they guarantee production of several millilitres of polyclonal reagent, and for monoclonals, at least 3 stable hybridoma clones per antigen, together with a supply of 10ml of hybridoma supernatant.

*Outcome – 6 hybridoma clones (3 for native antigen and 3 for fixed antigen).*

***Basic antisera and monoclonal antibody characterisation and IHC (Months 3-18).***

Antibodies supplied by Biogenes will initially be characterised by Western blot (using protein extracts from human myofibroblasts) and compared with commercial antibodies. A good antibody will recognise a single complex of 65kDa at a dilution of no less than 1:50. Specificity will be determined by the ability of the original phosphopeptide antigen to block the Western signal. Antibodies will also be tested for the ability to detect nuclear Rel-pSer536 in formalin-fixed and live human myofibroblasts prior to testing for IHC detection in sections of diseased human liver. We have access to an extensive archived bank of paraffin-embedded blocks of diseased liver tissue for these studies, including tissue from fibrotic NAFLD livers. Sections from normal and fibrotic liver will be de-waxed before gentle antigen retrieval using Vector labs unmasking solution, prior to blocking for endogenous avidin/biotin and non-specific protein binding, and overnight incubation with hybridoma supernatants (between 1:10 and 1:10,000 dilutions). Amplification will be achieved using the Vector Elite mouse and rabbit IgG kits, prior to DAB colour development and counter stain with Meyer’s haematoxylin (blue). High power light microscopy will be employed to determine which antibodies generate the best visualisation of RelA-pSer536 in fibrotic sections (taking into account factors such as background noise-to-signal ratio, antibody dilution, quality and intensity of nuclear stain, etc). Quality of immunohistochemistry will be compared with that obtained using a commercial antibody (eg. cell signalling technology; Phospho-NF-κB p65 (Ser536) (7F1) Mouse mAb #3036). Dual staining protocols will be developed for cell localisation (anti-αSMA for HMs), apoptosis (TUNEL) and proliferation (PCNA) which will be important for determining the association of RelA-pSer536 with fibrogenic HMs and loss of RelA-pSer536 with apoptotic HMs.

*Outcome – a panel of monoclonal antibodies recognising RelA-pSer536 which can be used for detailed IHC analyses in biopsy tissue.*

***Development of methods for the quantification and localisation of RelA-pSer536 in diseased liver (Months 12-24).***

The anti-RelA-pSer536 monoclonal (or in the event that these fail, polyclonal) antibody that gives the best IHC detection of antigen will be employed for development of methods to quantify antigen expression. We will initially develop a manual system in which a scientist blinded to the study will use high power light microscopy to count numbers of RelA-pSer536+ cells. Using the optimised dual IHC stains, we will also count numbers of RelA-pSer536+/α-SMA+ cells to generate specific counts for HMs, for numbers of RelA-pSer536+/PCNA+ (proliferation) cells and RelA-pSer536+/TUNEL+ (apoptosis) cells. These latter dual stains are important for determining if there is a mechanistic association between RelA-pSer536 and HMs proliferation/survival. We will also employ image analysis/morphometry (using the Leica DMR microscope and Leica Qwin image analysis software package) to provide quantitative assessment of RelA-pSer536 staining. For accurate morphometric analysis it is essential that there is minimal variation in IHC staining between slides arising from differences in the staining protocol. We will therefore validate use of an automated (Ventana) IHC staining system via collaboration with the academic haematology department. This system enables up to 480 slides to be processed per run and will be validated for RelA-pSer536, TUNEL and PCNA stains using slides generated from explant tissue (post-transplant cirrhotic liver).

*Outcome – quantitative methods for measurement of numbers of RelA-pSer536+ HMs and for association of the antigen with apoptotic or proliferative HMs.*

***Development of qRT-PCR assays for validation of anti-fibrotic activities of Losartan using liver biopsy material (Months 1-24).***

Molecular mechanisms by which Losartan promotes fibrosis regression will be investigated by analysis of the expression of NF-κB-target genes, regulators of apoptosis and genes that control fibrogenesis. This will be achieved by developing a panel of qRT-PCR assays (using the Applied Biosystems 7500 fast real-time PCR system) to determine differences in gene expression between pre- and post-treatment biopsy tissue and between responders and non-responders to Losartan therapy. Pathology and IHC studies demand a >16mm biopsy (we usually get 2 cm) and a paraffin block that makes 8-14 slides of 3-5 microns thick. We can safely remove a 0.5cm fraction of biopsy to produce sufficient total RNA for qRT-PCR analysis. To work up a protocol for use with diseased tissue we will initially use 0.5cm biopsy-like specimens from fibrotic human liver obtained from explant material and as control from “normal” liver (taken as part of liver resection with adjacent tumour tissue). Quality of cDNA generated from total RNA will be determined by control qPCRs (TaqMan) for β-actin and G-6PDH, prior to qPCR analysis of:

1. NF-κB target/inflammatory genes (IL-6, IL-8, TNFα, angiotensinogen, ICAM1 and MCP-1)

2. Apoptosis regulators (Gadd45β, Bcl2, Bcl-xL, c-FLIP, X-IAP and p52)

3. Fibrogenic genes (Collagen I, α-SMA, TIMP-1, TGFβ).

*Outcome – a validated method for qRT-PCR analysis of gene expression in biopsy tissue, and a panel of qRT-PCR assays that probe mechanism(s) of Losartan-induced fibrosis regression.*

***Application of RelA-pSer536 IHC and qRT-PCR biopsy assays on tissues from the Losartan trial (Months 24-36).***

IHC and qRT-PCR assays will be carried out once all pre- and post-biopsy tissues are collected and initial pathology analyses (grading of liver damage, inflammation and fibrosis) are completed. Slides will be coded and blinded to the scientist, and stained for RelA-pSer536, RelA-pSer536/αSMA, RelA-pSer536/TUNEL and RelA-pSer536/PCNA under identical conditions using the Ventana automated slide system. As we anticipate a total of 296 slides (148 patients) per stain, automation will ensure uniform IHC protocols which are essential for morphometry. Manual counts of stained cells will be determined by the scientist and verified by an independent pathologist (Prof AD Burt, Newcastle University) before codes are revealed. Data will undergo analysis of variance (ANOVA) to determine statistically-relevant differences between pre- and post-treatment, and between responders and non-responders. We will then cross-reference the data with fibrosis pathology scores to determine whether high numbers of RelA-pSer536+ HMs in the pre-biopsy tissue are a predictive indicator of a patient who will respond to Losartan therapy.

For qRT-PCR, quality of RNA will be determined using a Agilent 2100 Bioanalyzer, and quality of cDNA checked with reference to housekeeping genes (*β-actin, GAPDH, Histone 3, UBQ7*). We will then determine differences in expression of NF-κB target genes, apoptosis regulators and fibrogenic genes (genes to be measured in triplicate/biopsy cDNA to control for technical variability). ANOVA will identify statistically significant differences in gene expression between pre- and post-treatment biopsies, and between responders and non-responders.

*Outcomes – determine RelA-pSer536 is a biomarker for effective Losartan therapy and provide mechanistic insights into how Losartan stimulates regression of liver fibrosis.*

**10. Subject Population**

The study population will be patients with steatohepatitis and fibrosis, resulting from non-alcoholic fatty liver disease. The eligibility criteria have been deliberately kept as broad as possible to maximise recruitment and give the trial findings greater external validity.

**10.1 Inclusion criteria**

Adults (aged 18+), with steatohepatitis and fibrosis (Kleiner F1-F3), resulting from non-alcoholic fatty liver disease.

**10.2 Exclusion criteria**

1. Refusal or inability (lack of capacity) to give informed consent.
2. Average alcohol ingestion > 21 units per week (males) or > 14 units per week (females).
3. History or presence of Type 1 diabetes mellitus.
4. Haemoglobin A1C >15.0
5. Other causes of chronic liver disease or hepatic steatosis.
6. Any contra-indication to liver biopsy.
7. History of, or planned, gastrointestinal bypass surgery.
8. Hepatocellular carcinoma.
9. Previous liver transplantation.
10. Recent significant weight loss (>5% total body weight within last 6 months).
11. Electrolyte disturbance: potassium level outside the normal (local) range.
12. ALT or AST > 10 x ULN at screening.
13. Recent (within 6 months of baseline liver biopsy and screening visit) or concomitant use of agent known to cause hepatic steatosis (corticosteroids, amiodarone, methotrexate, tamoxifen, tetracycline, high dose oestrogens, valproic acid) or concomitant use of pioglitazone, fluconazole, rifampicin or any drug contra-indicated in the Losartan SmPC.
14. Introduction of metformin, glitazones, a GLP-1 agonist, Vitamin E or C, betaine, s-adenosyl methionine, ursodeoxycholic acid, silymarin, fibrate, pentoxifylline, orlistat, sibutramine or rimonabant within 3 months of baseline liver biopsy and screening visit.
15. Intolerance of ARBs or presence of multiple allergic reactions to drugs.
16. Use of ACE inhibitor or ARB in previous year.
17. Hypotension (Systolic < 100, diastolic < 60).
18. Renal failure (Cr >130).
19. Participation in any clinical study of an investigational agent within 30 days or five half-lives of the investigational product, whichever is longer.
20. Presence of clinically-relevant cardiovascular, pulmonary, gastro-intestinal, renal, hepatic, metabolic, haematological, neurological, psychiatric, systemic, ocular, gynaecological or acute infectious disease or signs of acute illness that, in the opinion of the investigator, might compromise the patient’s safe participation in the trial.
21. Presence or history of cancer in the last 5 years with the exception of adequately-treated localised basal cell carcinoma of the skin, in situ cervical carcinoma or solid malignancy surgically excised in toto without recurrence for five years.
22. Women of child-bearing potential not protected by an effective contraceptive method of birth control or surgical sterilization and/or who are unwilling or unable to be tested for pregnancy.

(Pregnancy status will be checked by serum pregnancy testing before initiation of study treatment and by urine pregnancy testing during the trial.)

1. Known allergy or sensitivity to Losartan or its excipients (microcrystalline cellulose (E460); lactose monohydrate; pregelatinized maize starch; magnesium stearate (E572); hydroxypropyl cellulose (E463); hypromellose (E464))

**11. Screening, Recruitment and Consent**

# 11.1 Screening

Potential participants will be identified at routine clinic outpatient appointments at trial sites and at approved Participant Identification Centres (PICs). All staff involved in identifying patients will hold substantive or honorary -contracts with the relevant hospital Trusts.

An eligibility screening form will be completed by the investigator to document the potential study participant’s fulfilment of the entry criteria. This will be done for all patients considered for the study and subsequently included or excluded. Anonymised demographic details of those excluded will be recorded, to facilitate ascertainment of the extent of participation bias. Reasons for exclusion will be recorded, in so far as is possible, to facilitate construction of the CONSORT diagram.

The screening assessments (as per routine clinical practice) will occur no more than 2 weeks prior to the baseline visit, randomisation and start of study drug. The initial liver biopsy must have been undertaken within 6 months of Visit 2.

# 11.2 Recruitment

Eligible participants will be invited to participate by the site PI or another member of site research staff with documented delegated responsibility, and the study explained to them. A study Patient Information Sheet will be provided at this time and the patient will be allowed to take this away for further consideration and discussion with family and/or friends.

A screening log will be kept to document details of subjects invited to participate in the study. For subjects who decline participation, this will document any reasons available for non-participation. The log will also ensure potential participants are only approached once.

# 11.3 Consent *(written consent must be obtained prior to any study specific procedures/visits/assessments/investigations)*

Informed consent discussions will be undertaken by appropriately-trained site staff (as per delegation log) involved in the study, including medical staff and research nurses, with the opportunity for participants to ask any questions. Following receipt of information about the study, participants will be given reasonable time (a minimum of 24 hours) to decide whether or not they would like to participate. Those wishing to take part will provide written informed consent by signing and dating the study consent form. The principal investigator or other member of site research staff with documented delegated authority will counter-sign and date it. Where the patient is unable to sign his/her name because of problems with literacy, or visual or motor impairments, verbal consent will be taken in the presence of an independent witness who will sign and date the consent form on behalf of the patient. Written informed consent should always be obtained prior to randomisation and the start of study specific procedures/investigations (including screening procedures which do not comprise normal clinical care).

The original signed consent form will be retained in the Investigator Site File, with a copy in the clinical notes and a copy provided to the participant. The participant will specifically consent to their GP being informed of their participation in the study.

The right to refuse to participate without giving reasons will be respected.

Due to the small subject population, the information sheet and consent form for the study will be available only in English. Interpreters will be arranged for all visits of patients who require them, either for verbal translation or for vision- or hearing-impaired individuals wishing to take part in the study, via local NHS arrangements. Qualified interpreters will be used to explain the consent form and information sheet, and great priority will be placed on finding the most direct form of communication.

# 11.4 Histopathological material

Diagnosis and staging of NASH will be done at the local hospital by the local histopathologist. At the end of treatment, the pre- and post-treatment paraffin-embedded biopsies will be sent to the delegated Histopathologist at the Histopathology Department in Newcastle University. These will be labelled with the patient’s randomisation code and sent in supplied pre-addressed Royal Mail packages. These will then be analysed by two blinded histopathologists for the primary outcome, fibrosis stage, and the secondary outcomes of steatosis, lobular inflammation and hepatocyte ballooning: the NAS score. In addition, sections will be cut for the RelA-pSer536, RelA-pSer536/αSMA, RelA-pSer536/TUNEL and RelA-pSer536/PCNA staining, to be done in the Mann laboratory.

In addition to the above, where possible, patients should have a further liver core taken at pre- and post-treatment biopsies which will be frozen and transported to the Mann laboratory for the qRT-PCR studies described above.

At the end of the study, histopathological samples will be stored in a NASH biobank with frozen serum and DNA, for further studies.

**11.5 Study Samples**

A recent collaboration with GlaxoSmithKline (GSK) and the Sponsor has been agreed to develop new biomarkers for fibrosis. The collection of samples and the process thereafter will be explained to patients, and a separate clause will be included in the consent form. If the patient agrees, serum, plasma and whole blood samples will be collected from patients at study visits as indicated in the study schedule. Patients may opt out of this part of the study. The samples provided will be collected and transferred to GSK laboratories or Clinical Research Organisations currently collaborating with GSK for analysis of biomarkers.

**12. Study Medication / Intervention Details**

**12.1 General Information for Study Medication**

Losartan (w.g. Cozaar®; Merck Sharp & Dohme Limited) is a synthetic oral angiotensin-II receptor (type AT_1_) antagonist, used in the treatment of essential hypertension in adults, for the treatment of renal disease in patients with hypertension and type 2 diabetes mellitus with proteinuria, and for the treatment of chronic heart failure. Losartan is, therefore, used outwith its Marketing Authorisation in the FELINE trial, and will be treated as an Investigational Medicinal Product (IMP). Losartan is used in this context because of the now overwhelming evidence that angiotensin II is pro-fibrotic, and that blocking the angiotensin II receptor can lead to stellate cell apoptosis in vitro, and fibrosis resolution.

In the FELINE trial, patients with NASH will be randomised to receive either Losartan or matching placebo (with blinding achieved by over-encapsulation) in a 1:1 ratio.

For reported side effects of Losartan please refer to section 19.2 Pharmacovigilance.

Please refer to the Summary of Product Characteristics (Appendix 1) for more detail.

Blinding of the study drug will be achieved by over-encapsulation in a Cellulose capsule shell, with an inert filler.

**12.2 Administration of Study Drug:**

Study medication will be labelled according to the requirements of Annex 13.

All study medication for a particular site will be provided by Catalent, clinical supplies company, to the site pharmacist, following site initiation. Study medication is for use by trial participants only.

Active and placebo study medication will be provided as an initial four-week and then 20-24 week supply of 50 mg capsules. Each participant pack will be presented as HDPE bottles with child resistant caps.

The starting dose will be 50mg Losartan once a day. This is the standard dose used in the treatment of hypertension and has been administered in previous proof-of-concept studies using Losartan to treat liver fibrosis: [2] Yokohama et al. *Therapeutic efficacy of an angiotensin* *II receptor antagonist in patients with non-alcoholic steatohepatitis.* Hepatology 2004 40(5): 1222-5 and [3] Colmenero et al, *Losartan reduces the expression of profibrogenic genes and inflammation in patients with chronic hepatitis C.* Hepatology 2007 46(4) Suppl. 1, 716A). Side effects will be documented by the participant throughout the course of the trial, and any participant reporting significant side effects will be withdrawn from the study.

A seven day visit window is allowed for each dispensing visit. For example, one bottle containing approx 37 capsules will be dispensed at Visit 2 (Baseline) for a maximum of four weeks; a second carton containing approx 148 capsules will be dispensed at Visit 4 (week four) for a maximum of 20 weeks. At Visits 5 (24 weeks), 6 (48 weeks) and 7 (72 weeks), a 24 week supply of capsules will be given (approx 185 capsules). The treatment will end at Visit 8 (week 96). Each visit date should be planned from the date of Visit 2, rather than the previous visit.

Study medication will be prescribed by a study clinician according to the protocol, and dispensed to the patient or clinical staff according to local pharmacy policy. Patients in possession of their study medication shall return all trial supplies in their original packaging (even if empty) to the study clinician for forwarding to the pharmacist. All returned, or unused, study medication will be stored in Pharmacy until the end of the study, or until the trial manager has completed appropriate reconciliation. It will then be destroyed.

Documentation for the prescribing, dispensing and return of study medication shall be maintained for study records.

At the end of the study, participants will discontinue the drug or placebo. It will be the decision of the patient’s clinical consultant as to whether they are prescribed Losartan after the results of the trial are known.

**12.3 Concomitant medication:**

A complete list of all concomitant medication (both prescribed medication and over-the-counter medication, including vitamin, mineral and other food supplements) received during the treatment phase must be recorded in the relevant CRF.

**12.3.1 Concomitant medication to avoid:**

Other antihypertensive agents may increase the hypotensive action of losartan. Concomitant use with other substances which may induce hypotension as an adverse reaction (like tricyclic

antidepressants, antipsychotics, baclofen and amifostine) may increase the risk of hypotension.

Losartan is predominantly metabolised by cytochrome P450 (CYP) 2C9 to the active carboxy acid metabolite. In a clinical trial it was found that fluconazole (inhibitor of CYP2C9) decreases the exposure to the active metabolite by approximately 50%. It was

found that concomitant treatment of losartan with rifampicin (inducer of metabolism enzymes) gave a 40% reduction in plasma concentration of the active metabolite. The clinical relevance of this effect is unknown. No difference in exposure was found with concomitant treatment with fluvastatin (weak inhibitor of CYP2C9).

As with other medicinal products that block angiotensin II or its effects, concomitant use of other medicinal products which retain potassium (e.g. potassium sparing diuretics: amiloride,

triamterene, spironolactone) or may increase potassium levels (e.g. heparin), potassium supplements or salt substitutes containing potassium may lead to increases in serum potassium. Co medication is not advisable.

Reversible increases in serum lithium concentrations and toxicity have been reported during concomitant administration of lithium with ACE inhibitors. Very rare cases have also been reported with angiotensin II receptor antagonists. Co administration of lithium and losartan should be undertaken with caution. If this combination proves essential, serum lithium level monitoring is recommended during concomitant use.

When angiotensin II antagonists are administered simultaneously with NSAIDs (i.e. selective COX 2 inhibitors, acetylsalicylic acid at anti inflammatory doses and non selective NSAIDs), attenuation of the antihypertensive effect may occur. Concomitant use of angiotensin II antagonists or diuretics and NSAIDs may lead to an increased risk of worsening of renal function, including possible acute renal failure, and an increase in serum potassium, especially in patients with poor pre existing renal function. The combination should be administered with caution, especially in the elderly. Patients should be adequately hydrated and consideration should be given to monitoring renal function after initiation of concomitant

therapy, and periodically thereafter.

**13. Randomisation**

A blocked allocation system will be used to allocate patients to the 2 groups (block size will not be disclosed to the investigators), with centre as a stratifying factor. Diabetes is a major risk factor for the progression of fibrosis in NASH. To reduce the risk of unequal proportions of diabetic and non-diabetic patients in active and placebo groups we will stratify for presence/absence of diabetes on randomisation of patients to Losartan or placebo.

Randomisation will generate a treatment number for each participant that links to the corresponding allocated study drug (blinded), in accordance with block size and strata. The treatment number must be clearly documented by the investigator on the trial prescription, to ensure the study pharmacist dispenses the correct study medication.

Randomisation will be administered centrally via Newcastle Clinical Trials Unit, using a system to ensure concealment of allocation.

Contact details for Randomisation: Please access the online randomisation system by using the following web address: <https://apps.ncl.ac.uk/random/>

If any problems with accessing the system or whilst using the system please contact The Institute of Health and Society Randomisation Service by email or by telephone number 0191 2227647 during office hours.

**14. Blinding**

Assignment to either active or placebo arm will be blinded to both the participant and investigator/assessor (double-blind). A code-break list will be kept in the pharmacy at participating hospitals; this list should be accessed only in an emergency and only when knowledge of allocation status would guide treatment decisions in that emergency (following discussion with the Principal Investigator or individual with delegated responsibility at site). Whenever the code is broken, the Chief Investigator will be informed immediately. If the code is broken, details including the participant number, who broke the code, why and when shall be recorded and maintained in the site file. Code breaks will not be routinely opened for participants who complete study treatment. Following a code break, should a clinician wish to supply clinical Losartan, this must then be supplied from normal routine pharmacy stock and not from clinical trial supplies.

At the final visit, the integrity of the blind will be assessed by asking both the participants and their treatment assessor: “Do you think you were taking Losartan or the dummy pill? Why do you think this?” The treatment assessor will record his/her answers before asking the participant in order to avoid bias.

**15. Study Data**

**15.1 Assessments / Data Collection**

Each study visit should last no more than 30 minutes.

The exception to this is the Baseline Visit where the number of assessments and

recording of background information will make this the longest visit, lasting up to two

hours.

A liver biopsy will be carried out within 6 months of Visit 2 and at Visit 8/end of treatment.

**SCHEDULE OF VISITS/EVENTS**

| **Procedure** | **Visit 1**  **Screening** | **Visit 2**  **Baseline** | **Visit 3 (1 week ± 2 days)** | **Visit 4 (4 weeks**  **± 7 days)** | **Visit 5 (24 weeks ± 7 days)** | **Visit 6 (48 weeks ± 7 days)** | **Visit 7 (72 weeks ± 7 days)** | **Visit 8 (96 weeks ± 7 days)**  **End of**  **treatment** | **Visit 9**  **(108 weeks**  **± 7 days)** |
| --- | --- | --- | --- | --- | --- | --- | --- | --- | --- |
| *Informed consent* | X |  |  |  |  |  |  |  |  |
| Demographics | X |  |  |  |  |  |  |  |  |
| Med history | X | - |  | - | - | - | - | - | - |
| *Leisure Time*  *Activity Assessment* | X |  |  |  |  |  |  |  |  |
| Physical exam | X | X |  | X | X | X | X | X | X |
| *Vital signs* | X | X | X | X | X | X | X | X |  |
| Height | X |  |  |  |  |  |  |  |  |
| Weight | X | X |  | X | X | X | X | X | X |
| *Waist circumference* | X | X | X | X | X | X | X | X | X |
| *ELF* | X |  |  |  |  | X |  | X |  |
| *ECG* | X |  |  |  |  |  |  |  |  |
| *Ultrasound ***** | X |  |  |  |  | X |  | X |  |
| *Fibroscan* *** | X |  |  |  |  | X |  | X |  |
| Biochemistry including urea and electrolytes and GGT | X | X | X | X | X | X | X | X | X |
| Haematology  FBC & Coagulation | X | X |  | X | X | X | X | X | X |
| *Pregnancy test (serum)* | X |  |  |  |  |  |  |  |  |
| *Pregnancy test (urine)* |  | X |  | X | X | X | X |  |  |
| *Liver biopsy* |  | ** |  |  |  |  |  | X |  |
| DNA sample |  | X |  |  |  |  |  |  |  |
| LFTs including AST | X | X |  | X | X | X | X | X | X |
| Lipid Panel | X | X |  | X | X | X | X | X | X |
| Glucose | X | X |  | X | X | X | X | X | X |
| Apolipoproteins A |  | X |  |  | X | X | X | X |  |
| HBA1C | X | X |  |  |  | X |  | X |  |
| CK 18 |  | X |  |  |  | X |  | X |  |
| Alpha-2 macroglobulin |  | X |  |  |  | X |  | X |  |
| Haptoglobins |  | X |  |  |  | X |  | X |  |
| Immunoglobulins |  | X |  |  |  |  |  | X |  |
| Insulin |  | X |  |  |  |  |  | X |  |
| C-peptide |  | X |  |  |  |  |  | X |  |
| *Serum sample* |  | X |  |  | X | X | X | X |  |
| *Samples for Biomarkers of Fibrosis (GSK sample)♦* |  | X |  | X | X | X | X | X | X |
|  |  |  |  |  |  |  |  |  |  |
| *CLDQ* |  | X |  |  |  | X |  | X |  |
| *SF-36* |  | X |  |  |  | X |  | X |  |
| *Randomisation* |  | X |  |  |  |  |  |  |  |
| *Dispense study medication* |  | X |  | X | X | X | X |  |  |
| *Review compliance with study medication* |  | X | X | X | X | X | X | X |  |
| *Adverse events* |  | X | X | X | X | X | X | X | X |
| *Concomitant medications* | X | X | X | X | X | X | X | X |  |

*** - Liver Biospy is required within 6 months of Visit 2.*

**** Patients with BMI of <35 should have a Fibroscan (where facilities are available)*

*****Ultrasound to be carried out +/- 1 month from screening date*

*♦ Patients may opt out of having these samples taken.*

*X* – *denotes routine clinical practice.*

**Patients will be asked to fast prior to all study visits APART from Visit 3: Week 1.**

***Visit 1: Screening***

The following procedures and assessments will be carried out at the screening visit:

1. Informed consent for participant.
2. Record demographics.

3. Medical history

Note that any history of the following represents an exclusion criterion:

a. Type 1 diabetes mellitus

b. Any other causes of chronic liver disease or hepatic steatosis

c. Any contra-indication to liver biopsy

d. History of or planned gastro-intestinal bypass surgery

e. Hepato-cellular carcinoma

f. Previous liver-transplant

g. Renal failure (Cr > 130)

h. Presence or history of cancer within the last 5 years, with the exception of adequately treated localised basal cell carcinoma of the skin, in situ cervical carcinoma or solid malignancy surgically excised in toto without recurrence for five years

i. Intolerance to ARBs or presence of multiple allergic reactions to drugs

j. Use of ACE inhibitor or ARB in previous year

k. Presence of clinically relevant cardiovascular, pulmonary, gastro-intestinal, renal, hepatic, metabolic, haematological, neurological, psychiatric, systemic, ocular, gynaecological or acute infectious disease or signs of acute illness that, in the opinion of the investigator, might compromise the patient’s safe participation in the trial

4. Leisure Time Activity Assessment – using the ipaq self-administered questionnaire (Ref Booth, M.L. (2000)

*Assessment of Physical Activity: An International Perspective.* Research Quarterly for Exercise and Sport, 71 (2): s114-20

5. Physical examination

6. Vital signs

a. Pulse

b. Blood pressure (Hypotension of systolic < 100 and diastolic < 60 represents an exclusion criterion)

7. Height

8. Weight

(Recent significant weight loss (> 5% total body weight within last 6 months represents an exclusion criterion)

9. Waist circumference

10. Enhanced Liver Fibrosis Test

11. ECG

12. Ultrasound (+/- 1 month from date of screening)

13. Fibroscan (if BMI <35)

14. Biochemistry profile (urea, electrolytes and GGT)

15. Haematology (Full blood count and coagulation)

16. Pregnancy Test, if applicable

Pregnancy status will be checked by serum pregnancy testing before initiation of study treatment and by urine pregnancy testing during the trial

17. Liver biopsy needs to be done within 6 months of Visit 2

18. Liver Function Tests including AST

(ALT or AST > 10 x ULN represents an exclusion criteria)

19. Lipid Panel

20. Glucose

21. HBA1C (>15.0 represents an exclusion criterion)

22. Record concomitant medications

***Visit 2: Baseline***

The following procedures and assessments will be carried out at the baseline visit:

1. Physical examination

2. Vital signs

3. Weight

4. Waist circumference

5. DNA sample (blood sample)

6. Biochemistry profile (urea, electrolytes and GGT)

7. Haematology (Full Blood Count and Coagulation)

8. Serum sample

9. A blood sample will be collected and serum, plasma and blood samples frozen to observe for novel serum markers of fibrosis that maybe discovered during the course of the study (GSK).

10. Liver Function Tests including AST

11. Lipid Panel

12. Glucose

13. Apolipoprotein A

14. HBA1C

15. CK 18

16. Alpha-2 macroglobulin

17. Haptoglobins

18. Immunoglobulins

19. Insulin

20. C-peptide

21. Chronic Liver Disease Questionnaire (CLDQ)

22. SF-36

23. Pregnancy test (urine sample)

24. Randomisation

25. Dispense study medication

26. Record concomitant medications.

***Visit 3: (1 week ± 2 days)***

The following procedures and assessments will be carried out at visit 3:

1. Vital signs
2. Biochemistry (urea, electrolytes and GGT)
3. Waist circumference
4. Review compliance with study medication
5. Record adverse events
6. Record concomitant medications

***Visit 4: (4 weeks ± 7days)***

The following procedures and assessments will be carried out at Visit 4:

1. Physical examination

2. Vital signs

3. Weight

4. Waist circumference

5 Biochemistry (urea, electrolytes and GGT)

6 Haematology (Full Blood Count and Coagulation)

7. Liver Function Tests including AST

8. A blood sample will be collected and serum, plasma and blood samples frozen to observe for novel serum markers of fibrosis that maybe discovered during the course of the study (GSK)

9. Lipid Panel

10. Glucose

11. Pregnancy test (urine sample)

12. Dispense study medication

13. Review compliance with study medication

14. Record adverse events

15. Record concomitant medications

***Visit 5 (24 weeks ± 7 days)***

1. Physical examination

2. Vital signs

3. Weight

4. Waist circumference

5. Biochemistry profile (urea, electrolytes and GGT)

6. Haematology (Full blood count and coagulation)

7. Liver function tests, including ASTs

8. Serum sample

9. A blood sample will be collected and serum, plasma and blood samples frozen to observe for novel serum markers of fibrosis that maybe discovered during the course of the study (GSK).

10. Lipid panel

11. Glucose

12. Apolipoprotein A

13. Pregnancy test (urine sample)

14. Dispense study medication

15. Review compliance with study medication

16. Record adverse events

17. Record concomitant medications.

***Visit 6 (48 weeks ± 7 days)***

1. Physical examination

2 Vital signs

3. Weight

4. Waist circumference

5. Enhanced Liver Fibrosis Test

6. Ultrasound

7. Fibroscan (if BMI <35)

8. Biochemistry profile (urea, electrolytes and GGT)

9. Haematology (Full blood count and coagulation)

10. Liver function tests including AST

11. Lipid panel

12. Glucose

13. Apolipoprotein A

14. HBA1C

15. CK 18

16. Alpha-2 macroglobulin

17. Haptoglobins

18. Serum sample

19. A blood sample will be collected and serum, plasma and blood samples frozen to observe for novel

serum markers of fibrosis that maybe discovered during the course of the study

(GSK).

20. Chronic Liver Disease Questionnaire (CLDQ)

21. SF-36

22. Pregnancy test (urine sample)

23. Dispense study medication

24. Review compliance with study medication

25. Record adverse events

26. Record concomitant medications.

***Visit 7 (72 weeks ± 7 days)***

1. Physical examination

2. Vital signs

3. Weight

4. Waist circumference

5. Biochemistry profile (urea, electrolytes and GGT)

6. Haematology (Full blood count and coagulation)

7. Liver function tests including AST

8. Serum sample

9. A blood sample will be collected and serum, plasma and blood samples frozen to observe for novel serum markers of fibrosis that maybe discovered during the course of the study (GSK).

10. Lipid panel

11. Glucose

12. Apolipoprotein A

13. Pregnancy test (urine sample)

14. Dispense study medication

15. Review compliance with study medication

16. Record adverse events

17. Record concomitant medications.

***Visit 8 (96 weeks ± 7 days)***

1. Physical examination

2. Vital signs

3 Weight

4. Waist circumference

5. Enhanced Liver Fibrosis Test

6. Ultrasound

7. Fibroscan (if BMI <35)

8. Biochemistry profile (urea, electrolytes and GGT)

9. Haematology (Full blood count and coagulation)

10. Liver biopsy

11 Liver function tests including ASTs

12. Serum sample

13. A blood sample will be collected and serum, plasma and blood samples frozen to observe for novel serum markers of fibrosis that maybe discovered during the course of the study (GSK).

14. Lipid panel

15. Glucose

16. Apolipoprotein A

17. HBA1C

18. CK 18

19. Alpha-2 macroglobulin

20. Haptoglobins

21. Immunoglobulins

22. Insulin

23. C-peptide

24. Chronic Liver Disease Questionnaire (CLDQ)

25. SF-36

26. Review compliance with study medication

27. Record adverse events

28. Record concomitant medications and check to see study medication returned.

***Visit 9 (108 weeks ± 7 days)***

1. Physical examination

2. Weight

3. Waist circumference

4. Biochemistry profile (urea, electrolytes and GGT)

5. Haematology (Full blood count and coagulation)

6. Liver function tests including ASTs

7. A blood sample will be collected and serum, plasma and blood samples frozen to observe for novel serum markers of fibrosis that maybe discovered during the course of the study (GSK).

8. Lipid panel

9. Glucose

10. Review compliance with study medication

11. Record adverse events

**15.2 Data Handling & Record Keeping**

Data collected on paper Case Report Forms will be entered by the Research Nurse at site, on a secure validated clinical data management system, or at the Study Centre.

Data will be handled, computerised and stored in accordance with the Data Protection Act 1998. No participant identifiable data will leave the study site.

The quality and retention of study data will be the responsibility of the Chief Investigator. All study data will be retained in accordance with the latest Directive on GCP (2005/28/EC) and local policy.

**16. Statistical Considerations**

Natural history studies have suggested that, over a two year period, fibrosis progresses in around 25% of patients with NAFLD [4](Lindor et al Hepatology 2004, 39(3) pp770-8). There is also regression in around a quarter of patients and the mean rate of progression throughout the population is very close to zero. Pilot data, from a small trial of Losartan in a post-transplant population with hepatitis C, have suggested that the proportion that progress could be brought to nearly 0% with regression of fibrosis in up to 50% of patients [3](Colmenero et al Hepatology, 2007 46(4), Suppl. 1, 716A). A further study giving Losartan for 48 weeks in seven patients with NASH showed a one point regression in fibrosis in four of seven patients with no progression in the other three. [2](Yokohama et al Hepatology 2004; 401222-5). The mean progression in this treated population is then -0.57 points per year. No studies are available to inform us as to what may happen in the second year of treatment. Using data available, we have made conservative estimates of what we will see over two years with placebo (10% progress by 2 points, 15% progress by 1 point, 55% no change, 20% regress by 1 point) and Losartan (5% progress by 2 points, 5% progress by 1 point, 48% no change, 42% regress by 1 point, 0% regress by 2 points). These figures give us a difference in mean change of fibrosis score between Losartan and placebo of 0.42 with a standard deviation of 0.84. This give us an effect size (mean difference between placebo and intervention in change in fibrosis score/SD) of 0.5.

In order to detect this effect, with 90% power with a significance of 0.05 (two tailed test), an achieved sample of 85 patients providing baseline and end-of-study biopsies will be required in each group, a total of 170. This achieved sample size would also provide 90% power, alpha = 0.05, to detect a difference in responder rate between placebo and intervention of 25% vs. 60%, and in rate of fibrosis progression of 25% vs. 3%.

Previous studies in this population have shown that loss to follow-up (including unwillingness to undergo a second biopsy, or inadequate biopsy results) is around 20% of patients so we would plan to recruit 214 patients. We estimate that 450 individuals will need to be screened to recruit this number of patients. Seven sites were anticipated to be sufficient in the first instance. Since the start of recruitment, observed rates of eligibility and consent have led to a revision of the number of sites and expansion in number of sites included.

Initially, each of the seven units approached, suggested that they could screen at least four patients per week, and therefore recruitment was scheduled to take a maximum of six months between the seven centres. To allow time for centre initiation, 12 months was factored in for recruitment. With a two-year follow-up and six months for analysis the study would take up to 42 months. Since the start of recruitment several more centres have agreed to participate but with a recent amendment to funding arrangements it has been decided that no further sites will be recruited.

**16.1 Statistical Analysis**

Given the early termination of recruitment to the study the proposed statistical analysis has been modified. The main output from the study will now relate to the recruitment process. Interval estimates of key parameters of interest will be calculated. These include: the proportion of patients approached that were eligible; proportion of eligible patients that agreed to be randomised; proportion of patients for who received treatment to which they were randomised; baseline demographics; level of data completeness; measures of central tendency and spread for all study outcomes. We will also produce an interval estimate of clinical effectiveness; examination of the upper and lower limits will give an indication of the reduced power to detect a clinically significant difference. Analysis will be on the basis of intention-to-treat. The primary analysis for efficacy will be based on change in fibrosis score, from baseline to end-of-treatment (month 0 to month 24). Differences between placebo and intervention groups will be assessed using analysis of covariance, with baseline fibrosis score as a covariate along with age, body mass index and presence of diabetes (factors previously associated with increased risk of progression). Assumptions underlying these tests will be checked and if necessary the effects of Losartan will be re-estimated using non-parametric procedures such as bootstrapping.

**17. Compliance and Withdrawal**

**17.1 Subject Compliance**

Where feasible, study visits will coincide with routine clinical follow-up, with good compliance expected. Follow-up visits will be at weeks 1, 4, 24, 48, 72, 96 and 108, relative to baseline. Visit windows of +/- seven days should ensure visit attendance; non-attendance for study visits will prompt follow-up by telephone.

Compliance with study medication will be assessed by checking and recording the remaining number of tablets at each visit. Study drug accountability will be assessed and documented by local pharmacy. The clinical team will also perform a quick review of any returned study medication at each study visit, to identify any obvious compliance concerns, and address these immediately with the participant.

**17.2 Withdrawal of participants**

## Participants have the right to withdraw from the study at any time for any reason. The investigator also has the right to withdraw patients from the study drug in the event of inter-current illness, AEs, SAEs, SUSARs, protocol violations, cure, administrative reasons or other reasons.

## It is understood by all concerned that an excessive rate of withdrawals can render the study uninterpretable; therefore, unnecessary withdrawal of patients should be avoided. Should a patient decide to withdraw from the study, all efforts will be made to report the reason for withdrawal as thoroughly as possible. Should a patient withdraw from the study drug only, efforts will be made to continue to obtain follow-up data, with the permission of the patient.

Individual patients may be withdrawn from therapy if they develop a side-effect of the medication; eg. hypotension, renal impairment or hyperkalaemia. If patients are withdrawn from therapy on the advice of treating clinicians, or at their own request, they will be asked to continue with follow-up assessments to 24 months. If they are unwilling to continue with follow-up visits for this length of time, they will be asked to consent to an ‘end of study’ visit, including non-invasive tests of liver function and fibrosis at the point of withdrawal. These tests will be the same as the patients completing the study would have at 96 weeks, apart from the liver biopsy.

Participants who wish to withdraw from study medication will be asked to confirm whether they are still willing to provide the following:

- study specific data (Visits 1 – 9).
- data collected as per routine clinical practice (Visits 1 – 9).

If participants agree to any of the above, they will be asked to complete a ‘confirmation of withdrawal’ form to document their decision, and the data they have provided will be retained.

**18. Data Monitoring, Quality Control and Quality Assurance**

**18.1 Discontinuation rules**

The trial may be prematurely discontinued on the basis of new safety information, or for other reasons given by the Data Monitoring & Ethics Committee / Trial Steering Committee, regulatory authority or ethics committee concerned.

Following 6 months of recruitment, initial rates of recruitment will be used to project total recruitment to ensure sufficient participants to power the trial. The Trial Steering Committee will advise on whether to continue, discontinue or extend the study (in time and/or in number of centres) and make a recommendation to the funder and sponsor. If the study is prematurely discontinued, active participants will be informed and no further participant data will be collected. A recent change in funding arrangements means that no further patients will be recruited.

**18.2 Monitoring, quality control and assurance**

The trial will be managed by the Newcastle Clinical Trials Unit. The Trial Management Group (TMG) will include: Professor C Day, Professor D Mann, Dr S. M^c^Pherson, Dr Q Anstee, Professor E McColl, Dr N Steen, Ms J Wilkinson, Ms J Barnes and Data Manager.

The Principal Investigators will be responsible for the day-to-day study conduct at site.

The Trial Manager will provide day-to-day support for the sites and provide training through Investigator meetings, site initiation visit and routine monitoring visits.

Quality control will be maintained through adherence to NCTU-wide SOPs, FELINE Study SOPs, study protocol, the principles of GCP, research governance and clinical trial regulations.

An independent Data Monitoring and Ethics Committee (DMEC: listed on Page 4) will be convened to undertake independent review. The roles and responsibilities of the DMEC are detailed in Appendix Two, but essentially the purpose of this committee is to monitor efficacy and safety endpoints. Only the DMEC will have access to unblinded study data. The committee will meet at least annually, and more often as appropriate, and meetings will be timed so that reports can be fed into the TSC. At the first meeting, the DMEC will discuss and advise on the inclusion of an interim analysis and possible adoption of a formal stopping rule for efficacy or safety.

A Trial Steering Committee (TSC) will be established to provide overall supervision of the trial; the roles and responsibilities of the TSC are set out in Appendix Three. Membership of the TSC is detailed on Page 4. The committee will meet annually, although there may be periods when more frequent meetings are necessary.

Monitoring of study conduct and data collected will be performed by a combination of central review and site monitoring visits to ensure that the study is conducted in accordance with GCP. Study site monitoring will be undertaken by the trial manager. The main areas of focus will include consent, serious adverse events, recording of primary outcome measures, essential documents in study files, and drug accountability and management.

Site monitoring will be determined following a full risk assessment of the study and

production of the study Monitoring Plan. It is anticipated that this will include:

- All original consent forms, reviewed as part of the study file.
- The presence of a copy of the consent form in the patient hospital notes. This will be confirmed for a selected number of patients as determined by the study risk assessment and Monitoring Plan.
- All original consent forms, compared against the study Participant Identification List and the Study Personnel Delegation Log.
- All reported serious adverse events, verified against treatment notes/medical records (source data verification).
- A check of the presence of all essential documents in the study file will be checked.
- Source data verification of primary endpoint data and eligibility data will be undertaken for a random sample of participants entered in the study. The numbers will be determined by the study risk assessment and Monitoring Plan.
- Drug accountability and management checked at site level.

Central monitoring will include:

- All applications for study authorisations or submissions of progress/safety reports will be reviewed for accuracy and completeness, prior to submission.
- All documentation essential for study initiation reviewed prior to site authorisation.
- Statistical monitoring of data, including outliers and inconsistencies.

All monitoring findings will be reported and followed up with the appropriate persons in a timely manner.

The study may be subject to inspection and audit by The Newcastle upon Tyne Hospitals

NHS Foundation Trust under their remit as sponsor, and other regulatory bodies (eg

MHRA) to ensure adherence to GCP. The investigator(s) / institutions will permit trial-

related monitoring, audits, REC review and regulatory inspection(s), providing direct

access to source data/documents.

**19. Pharmacovigilance**

**19.1 Definitions**

**Adverse event (AE):** Any untoward medical occurrence which does not necessarily have a causal relationship with the treatment. “Treatment” includes all investigational agents (including comparative agents) administered during the course of the study. Medical conditions/diseases present before starting study treatment are only considered adverse events if they worsen after starting study treatment.

**Adverse Reaction (AR):** Any untoward or unintended responses to an Investigational Medicinal Product (IMP) related to any dose administered. All AEs judged by either the reporting investigator or the sponsor as having reasonable causal relationship to a medicinal product qualify as adverse reactions. The expression “reasonable causal relationship” means to convey in general that there is evidence or argument to suggest a causal relationship.

***Causality:***

The assignment of the causality should be made by the investigator responsible for the care of the participant using the definitions in the table below. All adverse events judged as having a reasonable suspected causal relationship to the IMP(s) (i.e definitely, probably or possibly related) are considered to be adverse reactions. If any doubt about the causality exists, the local Principal Investigator should consult the Chief Investigator. In the case of discrepant views on causality between the Principal Investigator and others, all parties will discuss the case and will refer as necessary to the DMEC. In the event that no agreement is reached, the MHRA, main REC and other bodies will be informed of both points of view.

| **Relationship** | **Description** |
| --- | --- |
| **Unrelated** | There is no evidence of any causal relationship |
| **Unlikely** | There is little evidence to suggest there is a causal relationship (e.g. the event did not occur within a reasonable time after administration of the trial medication). There is another reasonable explanation for the event (e.g. the participant’s clinical condition, other concomitant treatment). |
| **Possible** | There is some evidence to suggest a causal relationship (e.g. because the event occurs within a reasonable time after administration of the trial medication). However, the influence of other factors may have contributed to the event (e.g. the participant’s clinical condition, other concomitant treatments). |
| **Probable** | There is evidence to suggest a causal relationship and the influence of other factors is unlikely. |
| **Definitely** | There is clear evidence to suggest a causal relationship and other possible contributing factors can be ruled out. |
| **Not assessable** | There is insufficient or incomplete evidence to make a clinical judgement of the causal relationship. |

**Serious Adverse Event** **(SAE)** or **Serious Adverse Reaction (SAR):** any untoward medical occurrence or effect that at any dose

- Results in death
- Is life-threatening – refers to an event in which the subject was at risk of death at the time of the event; it does not refer to an event which hypothetically might have caused death if it were more severe
- Requires hospitalisation, or prolongation of existing in-patients’ hospitalisation
- Results in persistent or significant disability or incapacity
- Is a congenital anomaly or birth defect

Medical judgement should be exercised in deciding whether an AE/AR is serious in other situations. Important AE/ARs that are not immediately life-threatening or do not result in death or hospitalisation but may jeopardise the subject or may require intervention to prevent one of the other outcomes listed in the definition above, should also be considered serious.

**Suspected, Unexpected Serious Adverse Reaction (SUSAR):** an adverse reaction that is both unexpected and serious. An adverse reaction is ‘unexpected’ if its nature or severity is not consistent with the applicable product information (see section 19.2).

**19.2 Expected adverse reactions**

Most adverse events and adverse drug reactions that occur in this study, whether they are serious or not, will be expected treatment-related toxicities due to the drugs used in this study.

The most common known adverse reactions (in a range of clinical indications) to Losartan are: dizziness, vertigo, hypotension, asthenia/fatigue, hypoglycaemia and hyperkalaemia.

For a full list of expected undesirable effects of Losartan, please refer to the Summary of Product Characteristics for Losartan (Appendix One).

**19.3 Protocol Specifications**

For purposes of this protocol

- All non-serious adverse reactions will be recorded at all visits.
- Any serious adverse events will be recorded throughout the duration of the trial until after trial therapy is stopped.
- Serious adverse events exclude any pre-planned hospitalisations not associated with clinical deterioration.
- Serious adverse events exclude routine treatment or monitoring of the studied indication, not associated with any deterioration in condition.
- Serious adverse events exclude elective or scheduled treatment for pre-existing conditions that did not worsen during the study.
- Serious adverse events exclude the FELINE study primary outcome measure of change in fibrosis status, which is already documented and monitored within study.

**19.4 Recording & Reporting Adverse Events or Reactions**

All adverse events should be reported. Depending on the nature of the event, the reporting procedures below should be followed. Any questions concerning adverse event reporting should be directed to the Chief Investigator (or Trial Manager in the absence of the Chief Investigator) in the first instance. A flowchart (figure X) is given below to aid in the reporting procedures.

**Adverse Event (including Adverse Reaction):** Severity of AEs will be graded on a three-point scale (mild, moderate, severe). Relation of the AE to the treatment (causality) should be assessed by the investigator at site.

All non-serious adverse events / reactions during drug treatment will be reported on the study CRF and sent to the Trial Manager within four weeks of the visit at which the AE/AR was ascertained.

**Serious Adverse Event / Reaction (SAE/SAR, including SUSARs):**

Serious Adverse Events (SAEs) and Serious Adverse Reactions (SARs, including SUSARS and expected SARs) will be reported to the Chief Investigator and Trial Management Team within 24 hours of the site Principal Investigator becoming aware of the SAE/SAR/SUSAR.

The initial report can be made by secure fax which will also generate an email copy to the Chief Investigator, Senior Trial Manager and Trial Manager. In the case of incomplete information at the time of initial reporting, all appropriate information should be provided as follow-up, on the appropriate SAE follow-up form. As indicated above, relationship of the SAE to the treatment (causality) should be assessed by the investigator at site, as should the expected or unexpected nature (by reference to the SmPC for Losartan) of any serious adverse reactions.

The MHRA and REC will be notified, by the Trial Management Team, of all SUSARs occurring during the study according to the following timelines; fatal and life-threatening within 7 days of notification and non-life threatening within 15 days. All investigators will be informed of all SUSARs occurring throughout the study on a case-by-case basis.

The Chief Investigator will ensure that the Newcastle upon Tyne Hospitals NHS Foundation Trust, as sponsor, is notified of any SUSARs in accordance with local Trust policy.

Local investigators should report any SUSARs and / or SAEs as required by their local Research & Development Office.

***Figure 1***

| **Adverse event** | | | | | | |
| --- | --- | --- | --- | --- | --- | --- |
| **Not Serious** | | **Not sure** |  | **Serious** | |  |
| **Unrelated** | **Related** |  | **Unrelated** | | **Related** | |
|  |  |  |  | | Expected | **Unexpected** |
| **Complete**  **CRFs** | **Complete**  **CRFs** | **Complete SAE form or contact CI** | **Complete SAE form** | | **Complete SAE form** | **Complete SAE form** |

Contact details for reporting SAEs and SUSARs

Please send SAE form(s) via Fax number: 0191 5800707 or

Tel: 0191 2227647 (Mon to Fri 09.00 – 17.00)

**19.5. Pregnancies**

**19.5.1. Time period for collecting pregnancy information**

All pregnancies in female subjects and female partners of male subjects will be collected after the start of dosing and until last follow-up visit.

**19.5.2. Action to be taken if pregnancy occurs**

The investigator will collect pregnancy information on any female subject who becomes pregnant while participating in this study. The investigator will record pregnancy information on the appropriate form, and submit it to the Chief Investigator within 2 weeks of learning of a subject's pregnancy. The subject will also be followed-up to determine the outcome of the pregnancy. Information on the status of the mother and child will be forwarded to the Chief Investigator. Generally, follow-up will be no longer than 8-12 weeks following the estimated delivery date.

Any premature termination of the pregnancy will be reported. While pregnancy itself is not considered to be an AE or SAE, any pregnancy complication or elective termination of a pregnancy for medical reasons will be recorded as an AE or SAE (see AE/SAE section of the protocol for definitions and a description of follow-up). A spontaneous abortion is always considered to be an SAE and will be reported as such.

Furthermore, any SAE occurring as a result of a post-study pregnancy and considered reasonably related to the investigational product by the investigator, will be reported to the Chief Investigator. While the investigator is not obliged actively to seek this information in former study participants, he or she may learn of an SAE through spontaneous reporting.

Any female participant who becomes pregnant while participating will be withdrawn from the study.

Any female participant who becomes pregnant during dosing, will stop dosing immediately.

**20. Ethics & Regulatory Issues**

The conduct of this study will be in accordance with the recommendations for physicians involved in research on human subjects adopted by the 18th World Medical Assembly, Helsinki 1964 and later revisions, up to and including the April 2008 version.

Favourable ethical opinion from a Type 3 Research Ethics Committee, R&D approval via the NIHR Coordinated System for gaining NHS Permissions and Clinical Trial Authorisation from the Medicines and Healthcare Products Regulatory Agency will be sought prior to commencement of the study. Local R&D approval will be sought before recruitment may commence at each site. The Trial Management Team will require a written copy of the local approval documentation, before initiating each centre and accepting participants into the study.

Information sheets will be provided to all eligible subjects and written informed consent obtained prior to any study procedures being carried out. For subjects who cannot provide written informed consent for themselves because of vision, literacy or motor problems an appropriate independent witness will witness the oral consent. Individuals who do not have the capacity to provide informed consent will be excluded from the study.

**21. Confidentiality**

Personal data will be regarded as strictly confidential. To preserve anonymity, any data leaving the site will identify participants by their initials, date of birth, and a unique study identification code only. The study will comply with the Data Protection Act, 1998. All study records, including signed consent forms and Investigator Site Files will be kept at site, in a locked filing cabinet with restricted access.

All laboratory samples will be labelled with a unique study identification number and patient’s initials and date of birth only (linked in anonymised form).

**22. Insurance and Finance**

The participating NHS Trusts have liability for clinical negligence that harms individuals toward whom they have a duty of care. Indemnity in respect of negligent harm arising from study management is provided via NHS schemes by The Newcastle upon Tyne Hospitals NHS Foundation Trust in its role as Sponsor. Indemnity in respect of negligent harm arising from study conduct is provided by NHS schemes, via the participating NHS Trusts, covering NHS-employed staff and medical academic staff with honorary NHS contracts who are conducting the trial. Indemnity in respect of negligent harm arising from study design / protocol authorship is provided by NHS schemes for those protocol authors whose substantive contract of employment lies with the NHS, and via Newcastle University insurance for protocol authors who have their substantive contract with the University. This is a non-commercial study and there are no arrangements for non-negligent compensation.

The NIHR Efficacy and Mechanism Evaluation Programme (NIHR EME) has provided funding up until 01/01/13. Additional funding has been secured and Newcastle NIHR BRC will continue to fund the current patient follow-ups.

**23. Study Report / Publications**

The data will be the property of the Chief Investigator and Co-Investigator(s). Publication will be the responsibility of the Chief Investigator.

It is planned to publish this study in peer-reviewed journals and to present data orally and/or in posters, at national and international meetings. Results of the study will also be reported to the Sponsor and Funder, and will be available on the Funder’s web site. All manuscripts, abstracts or other modes of presentation will be reviewed by the Funder prior to submission. Individuals will not be identified from any study report.

Participants will be informed about their treatment and their contribution to the study at the end of the study, and will be provided with a lay summary of the results.

**24. References**

Provide the citation for all publications referenced in the text of the protocol.

**1. Newton, J.L., et al., *Fatigue in non-alcoholic fatty liver disease (NAFLD) is significant and associates with inactivity and excessive daytime sleepiness but not with liver disease severity or insulin resistance.* Gut, 2008. 57(6): p. 807-13.**

**2. Yokohama, S., et al., *Therapeutic efficacy of an angiotensin II receptor antagonist in patients with nonalcoholic steatohepatitis.* Hepatology, 2004. 40(5): p. 1222-5.**

**3. Colmenero, J., et al., *Losartan reduces the expression of profibrogenic genes and inflammation in patients with chronic hepatitis C* Hepatology, 2007. 46(4, S1): p. 716A-717A.**

**4. Lindor, K.D., et al., *Ursodeoxycholic acid for treatment of nonalcoholic steatohepatitis: results of a randomized trial.* Hepatology, 2004. 39(3): p. 770-8.**

**5. Adams, L.A., et al., *The histological course of nonalcoholic fatty liver disease: a longitudinal study of 103 patients with sequential liver biopsies.* Journal of Hepatology, 2005. 42(1): p. 132-138.**

**6. Adams, L.A., et al., *The natural history of nonalcoholic fatty liver disease: a population-based cohort study.* Gastroenterology, 2005. 129(1): p. 113-21.**

**7. Hepatology 2006. 43(S1): p. S82-8.**

**8. International Journal of Biochemistry & Cell Biology 2007. 39: p. 695-714.**

**9. Wright, M.C., et al., *Gliotoxin stimulates the apoptosis of human and rat hepatic stellate cells and enhances the resolution of liver fibrosis in rats.* Gastroenterology, 2001. 121(3): p. 685-98.**

**10. Watson, M.R., et al., *NF-kappaB is a critical regulator of the survival of rodent and human hepatic myofibroblasts.* Hepatology, 2008. 48(4): p. 589-97.**

**11. Oakley, F., et al., *Inhibition of inhibitor of kappaB kinases stimulates hepatic stellate cell apoptosis and accelerated recovery from rat liver fibrosis.* Gastroenterology, 2005. 128(1): p. 108-20.**

**12. Bataller, R., et al., *Systemic infusion of angiotensin II exacerbates liver fibrosis in bile duct-ligated rats.* Hepatology, 2005. 41(5): p. 1046-55.**

**13. Bataller, R., et al., *Activated human hepatic stellate cells express the renin-angiotensin system and synthesize angiotensin II.* Gastroenterology, 2003. 125(1): p. 117-25.**

**14. Kleiner, D.E., et al., *Design and validation of a histological scoring system for nonalcoholic fatty liver disease.* Hepatology, 2005. 41(6): p. 1313-21.**

**25. Appendices**

**APPENDIX ONE – Summary of Product Characteristics for Losartan**

*
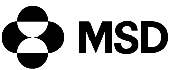
*

| **Merck Sharp & Dohme Limited** |  |
| --- | --- |
| Hertford Road, Hoddesdon, Hertfordshire, EN11 9BU  Telephone: +44 (0)1992 467 272  Fax: +44 (0)1992 451 066  1. NAME OF THE MEDICINAL PRODUCT   |  | Cozaar®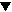* 12.5 mg film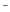coated tablets  Cozaar®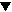* 25 mg film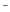coated tablets  Cozaar®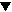* 50 mg film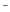coated tablets  Cozaar®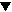* 100 mg film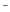coated tablets  ** Intensive monitoring is requested only when used for the recently-licensed indication in chronic heart failure* |  | | --- | --- | --- |   [Go to top of the page](http://emc.medicines.org.uk/medicine/8320/SPC/COZAAR+12.5+mg%2C+25+mg%2C+50+mg+and+100+mg+Film-Coated+Tablets/#tableOfContents)  2. QUALITATIVE AND QUANTITATIVE COMPOSITION   |  | Each COZAAR 12.5 mg Tablet contains 12.5 mg of losartan potassium.  Each COZAAR 25 mg Tablet contains 25 mg of losartan potassium.  Each COZAAR 50 mg Tablet contains 50 mg of losartan potassium.  Each COZAAR 100 mg Tablet contains 100 mg of losartan potassium.  Each COZAAR 12.5 mg tablet contains 25.25 mg lactose monohydrate.  Each COZAAR 25 mg tablet contains 12.75 mg lactose monohydrate.  Each COZAAR 50 mg tablet contains 25.5 mg lactose monohydrate.  Each COZAAR 100 mg tablet contains 51.0 mg lactose monohydrate.  For a full list of excipients, see section 6.1. |  | | --- | --- | --- |   [Go to top of the page](http://emc.medicines.org.uk/medicine/8320/SPC/COZAAR+12.5+mg%2C+25+mg%2C+50+mg+and+100+mg+Film-Coated+Tablets/#tableOfContents)  3. PHARMACEUTICAL FORM   |  | Film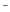coated tablets  COZAAR 12.5 mg tablet  Blue, oval film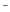coated tablets marked 11 on one side and plain on the other.  COZAAR 25 mg tablet  White, oval unscored film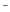coated tablets marked 951 on one side and plain on the other.  COZAAR 50 mg tablet  White, oval film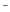coated tablets marked 952 on one side and scored on the other.  The tablet can be divided into equal halves.  COZAAR 100 mg tablet  White, teardrop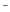shaped film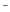coated tablets marked 960 on one side and plain on the other. |  | | --- | --- | --- |   [Go to top of the page](http://emc.medicines.org.uk/medicine/8320/SPC/COZAAR+12.5+mg%2C+25+mg%2C+50+mg+and+100+mg+Film-Coated+Tablets/#tableOfContents)  4. CLINICAL PARTICULARS   |  |  |  | | --- | --- | --- |   [Go to top of the page](http://emc.medicines.org.uk/medicine/8320/SPC/COZAAR+12.5+mg%2C+25+mg%2C+50+mg+and+100+mg+Film-Coated+Tablets/#tableOfContents)  4.1 Therapeutic indications   |  | • Treatment of essential hypertension in adults and in children and adolescents 6-18 years of age.  • Treatment of renal disease in adult patients with hypertension and type 2 diabetes mellitus with proteinuria 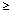0.5 g/day as part of an antihypertensive treatment.  • Treatment of chronic heart failure (in patients 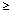60 years), when treatment with Angiotensin converting enzyme (ACE) inhibitors is not considered suitable due to incompatibility*, especially cough,* or contraindication. Patients with heart failure who have been stabilised with an ACE inhibitor should not be switched to losartan. The patients should have a left ventricular ejection fraction 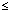40% and should be clinically stable and on an established treatment regimen for chronic heart failure.  • Reduction in the risk of stroke in adult hypertensive patients with left ventricular hypertrophy documented by ECG (see section 5.1 LIFE study, Race). |  | | --- | --- | --- |     4.2 Posology and method of administration   |  | Losartan tablets should be swallowed with a glass of water. COZAAR may be administered with or without food.  Hypertension  The usual starting and maintenance dose is 50 mg once daily for most patients. The maximal antihypertensive effect is attained 3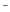6 weeks after initiation of therapy. Some patients may receive an additional benefit by increasing the dose to 100 mg once daily (in the morning).  Losartan may be administered with other antihypertensive agents, especially with diuretics (e.g. hydrochlorothiazide).  Hypertensive type II diabetic patients with proteinuria 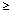0.5 g/day  The usual starting dose is 50 mg once daily. The dose may be increased to 100 mg once daily based on blood pressure response from one month onwards after initiation of therapy. Losartan may be administered with other antihypertensive agents (e.g. diuretics, calcium channel blockers, alpha- or beta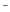blockers, and centrally acting agents) as well as with insulin and other commonly used hypoglycemic agents (e.g.sulfonylureas, glitazones and glucosidase inhibitors).  Heart failure  The usual initial dose of losartan in patients with heart failure is 12.5 mg once daily. The dose should generally be titrated at weekly intervals (i.e. 12.5 mg daily, 25 mg daily, 50 mg daily) to the usual maintenance dose of 50 mg once daily, as tolerated by the patient.  Reduction in the risk of stroke in hypertensive patients with left ventricular hypertrophy documented by ECG  The usual starting dose is 50 mg of losartan once daily. A low dose of hydrochlorothiazide should be added and/ or the dose of losartan should be increased to 100 mg once daily based on blood pressure response.  *Special populations*  Use in patients with intravascular volume depletion:  For patients with intravascular volume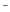depletion (e.g. those treated with high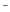dose diuretics), a starting dose of 25 mg once daily should be considered (see section 4.4).  Use in patients with renal impairment and haemodialysis patients:  No initial dosage adjustment is necessary in patients with renal impairment and in haemodialysis patients.  Use in patients with hepatic impairment:  A lower dose should be considered for patients with a history of hepatic impairment. There is no therapeutic experience in patients with severe hepatic impairment. Therefore, losartan is contraindicated in patients with severe hepatic impairment (see sections 4.3 and 4.4).  Use in paediatric patients  There are limited data on the efficacy and safety of losartan in children and adolescents aged 6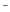18 years old for the treatment of hypertension (see section 5.1). Limited pharmacokinetic data are available in hypertensive children above one month of age (see section 5.2).  For patients who can swallow tablets, the recommended dose is 25 mg once daily in patients>20 to <50 kg. (In exceptional cases the dose can be increased to a maximum of 50 mg once daily). Dosage should be adjusted according to blood pressure response.  In patients>50 kg, the usual dose is 50 mg once daily. In exceptional cases the dose can be adjusted to a maximum of 100 mg once daily. Doses above 1.4 mg/ kg (or in excess of 100 mg) daily have not been studied in paediatric patients.  Losartan is not recommended for use in children under 6 years old, as limited data are available in these patient groups.  It is not recommended in children with glomerular filtration rate < 30 ml/ min / 1.73 m^2^, as no data are available (see also section 4.4).  Losartan is also not recommended in children with hepatic impairment (see also section 4.4).  Use in Elderly  Although consideration should be given to initiating therapy with 25 mg in patients over 75 years of age, dosage adjustment is not usually necessary for the elderly. |  | | --- | --- | --- |   [Go to top of the page](http://emc.medicines.org.uk/medicine/8320/SPC/COZAAR+12.5+mg%2C+25+mg%2C+50+mg+and+100+mg+Film-Coated+Tablets/#tableOfContents)  4.3 Contraindications   |  | Hypersensitivity to the active substance or to any of the excipients (see section 4.4 and 6.1).  2nd and 3rd trimester of pregnancy (see section 4.4 and 4.6).  Severe hepatic impairment. |  | | --- | --- | --- |   [Go to top of the page](http://emc.medicines.org.uk/medicine/8320/SPC/COZAAR+12.5+mg%2C+25+mg%2C+50+mg+and+100+mg+Film-Coated+Tablets/#tableOfContents)  4.4 Special warnings and precautions for use   |  | Hypersensitivity  *Angio-oedema.* Patients with a history of angio-oedema (swelling of the face, lips, throat, and/ or tongue) should be closely monitored (see section 4.8).  Hypotension and electrolyte/fluid imbalance  Symptomatic hypotension, especially after the first dose and after increasing of the dose, may occur in patients who are volume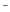 and/or sodium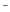depleted by vigorous diuretic therapy, dietary salt restriction, diarrhoea or vomiting. These conditions should be corrected prior to administration of losartan, or a lower starting dose should be used (see section 4.2). This also applies to children 6 to 18 years of age.  Electrolyte imbalances  Electrolyte imbalances are common in patients with renal impairment, with or without diabetes, and should be addressed. In a clinical study conducted in type 2 diabetic patients with nephropathy, the incidence of hyperkalemia was higher in the group treated with losartan as compared to the placebo group (see section 4.8). Therefore, the plasma concentrations of potassium as well as creatinine clearance values should be closely monitored, especially patients with heart failure and a creatinine clearance between 30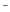50 ml/ min should be closely monitored.  The concomitant use of potassium-sparing diuretics, potassium supplements and potassium-containing salt substitutes with losartan is not recommended (see section 4.5).  Hepatic impairment  Based on pharmacokinetic data which demonstrate significantly increased plasma concentrations of losartan in cirrhotic patients, a lower dose should be considered for patients with a history of hepatic impairment. There is no therapeutic experience with losartan in patients with severe hepatic impairment. Therefore losartan must not be administered in patients with severe hepatic impairment (see sections 4.2, 4.3 and 5.2).  Losartan is not recommended in children with hepatic impairment (see section 4.2).  Renal impairment  As a consequence of inhibiting the renin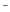angiotensin system, changes in renal function including renal failure have been reported (in particular, in patients whose renal function is dependent on the renin- angiotensin-aldosterone system such as those with severe cardiac insufficiency or pre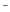existing renal dysfunction). As with other medicinal products that affect the renin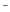angiotensin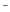aldosterone system, increases in blood urea and serum creatinine have also been reported in patients with bilateral renal artery stenosis or stenosis of the artery to a solitary kidney; these changes in renal function may be reversible upon discontinuation of therapy. Losartan should be used with caution in patients with bilateral renal artery stenosis or stenosis of the artery to a solitary kidney.  *Use in paediatric patients with renal impairment*  Losartan is not recommended in children with glomerular filtration rate < 30 ml/ min/ 1.73 m^2^ as no data are available (see section 4.2).  Renal function should be regularly monitored during treatment with losartan as it may deteriorate. This applies particularly when losartan is given in the presence of other conditions (fever, dehydration) likely to impair renal function.  Concomitant use of losartan and ACE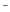inhibitors has shown to impair renal function. Therefore, concomitant use is not recommended (see section 4.5).  Renal transplantation  There is no experience in patients with recent kidney transplantation.  Primary hyperaldosteronism  Patients with primary aldosteronism generally will not respond to antihypertensive medicinal products acting through inhibition of the renin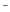angiotensin system. Therefore, the use of losartan is not recommended.  Coronary heart disease and cerebrovascular disease  As with any antihypertensive agents, excessive blood pressure decrease in patients with ischaemic cardiovascular and cerebrovascular disease could result in a myocardial infarction or stroke.  Heart failure  In patients with heart failure, with or without renal impairment, there is 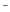as with other medicinal products acting on the renin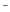angiotensin system 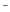a risk of severe arterial hypotension, and (often acute) renal impairment.  There is no sufficient therapeutic experience with losartan in patients with heart failure and concomitant severe renal impairment, in patients with severe heart failure (NYHA class IV) as well as in patients with heart failure and symptomatic life threatening cardiac arrhythmias. Therefore, losartan should be used with caution in these patient groups. The combination of losartan with a beta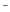blocker should be used with caution (see section 5.1).  Aortic and mitral valve stenosis, obstructive hypertrophic cardiomyopathy  As with other vasodilators, special caution is indicated in patients suffering from aortic or mitral stenosis, or obstructive hypertrophic cardiomyopathy.  Excipients  This medicinal product contains lactose. Patients with rare hereditary problems of galactose intolerance, the Lapp lactase deficiency or glucose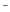galactose malabsorption should not take this medicine.  Pregnancy  Losartan should not be initiated during pregnancy. Unless continued losartan therapy is considered essential, patients planning pregnancy should be changed to alternative anti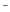hypertensive treatments which have an established safety profile for use in pregnancy. When pregnancy is diagnosed, treatment with losartan should be stopped immediately, and, if appropriate, alternative therapy should be started (see sections 4.3 and 4.6).  Other warnings and precautions  As observed for angiotensin converting enzyme inhibitors, losartan and the other angiotensin antagonists are apparently less effective in lowering blood pressure in black people than in non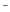blacks, possibly because of higher prevalence of low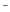renin states in the black hypertensive population. |  | | --- | --- | --- |   [Go to top of the page](http://emc.medicines.org.uk/medicine/8320/SPC/COZAAR+12.5+mg%2C+25+mg%2C+50+mg+and+100+mg+Film-Coated+Tablets/#tableOfContents)  4.5 Interaction with other medicinal products and other forms of interaction   |  | Other antihypertensive agents may increase the hypotensive action of losartan. Concomitant use with other substances which may induce hypotension as an adverse reaction (like tricyclic antidepressants, antipsychotics, baclofen and amifostine) may increase the risk of hypotension.  Losartan is predominantly metabolised by cytochrome P450 (CYP) 2C9 to the active carboxy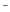acid metabolite. In a clinical trial it was found that fluconazole (inhibitor of CYP2C9) decreases the exposure to the active metabolite by approximately 50%. It was found that concomitant treatment of losartan with rifampicin (inducer of metabolism enzymes) gave a 40% reduction in plasma concentration of the active metabolite. The clinical relevance of this effect is unknown. No difference in exposure was found with concomitant treatment with fluvastatin (weak inhibitor of CYP2C9).  As with other medicinal products that block angiotensin II or its effects, concomitant use of other medicinal products which retain potassium (e.g. potassium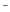sparing diuretics: amiloride, triamterene, spironolactone) or may increase potassium levels (e.g. heparin), potassium supplements or salt substitutes containing potassium may lead to increases in serum potassium. Co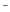medication is not advisable.  Reversible increases in serum lithium concentrations and toxicity have been reported during concomitant administration of lithium with ACE inhibitors. Very rare cases have also been reported with angiotensin II receptor antagonists. Co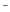administration of lithium and losartan should be undertaken with caution. If this combination proves essential, serum lithium level monitoring is recommended during concomitant use.  When angiotensin II antagonists are administered simultaneously with NSAIDs (i.e. selective COX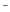2 inhibitors, acetylsalicylic acid at anti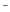inflammatory doses and non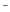selective NSAIDs), attenuation of the antihypertensive effect may occur. Concomitant use of angiotensin II antagonists or diuretics and NSAIDs may lead to an increased risk of worsening of renal function, including possible acute renal failure, and an increase in serum potassium, especially in patients with poor pre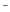existing renal function. The combination should be administered with caution, especially in the elderly. Patients should be adequately hydrated and consideration should be given to monitoring renal function after initiation of concomitant therapy, and periodically thereafter. |  | | --- | --- | --- |   [Go to top of the page](http://emc.medicines.org.uk/medicine/8320/SPC/COZAAR+12.5+mg%2C+25+mg%2C+50+mg+and+100+mg+Film-Coated+Tablets/#tableOfContents)  4.6 Pregnancy and lactation   |  | Pregnancy  The use of losartan is not recommended during the first trimester of pregnancy (see section 4.4). The use of losartan is contra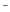indicated during the 2nd and 3rd trimester of pregnancy (see section 4.3 and 4.4).  Epidemiological evidence regarding the risk of teratogenicity following exposure to ACE inhibitors during the first trimester of pregnancy has not been conclusive; however a small increase in risk cannot be excluded. Whilst there is no controlled epidemiological data on the risk with Angiotensin II Receptor Inhibitors (AIIRAs), similar risks may exist for this class of medicinal products. Unless continued AIIRA therapy is considered essential, patients planning pregnancy should be changed to alternative anti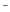hypertensive treatments which have an established safety profile for use in pregnancy. When pregnancy is diagnosed, treatment with losartan should be stopped immediately and, if appropriate, alternative therapy should be started.  Exposure to AIIA therapy during the second and third trimesters is known to induce human fetotoxicity (decreased renal function, oligohydramnios, skull ossification retardation) and neonatal toxicity (renal failure, hypotension, hyperkalaemia) (see also 5.3). Should exposure to losartan have occurred from the second trimester of pregnancy, ultrasound check of renal function and skull is recommended.  Infants whose mothers have taken losartan should be closely observed for hypotension (see also section 4.3 and 4.4).  Lactation  Because no information is available regarding the use of losartan during breastfeeding, losartan is not recommended and alternative treatments with better established safety profiles during breastfeeding are preferable, especially while nursing a newborn or preterm infant. |  | | --- | --- | --- |   [Go to top of the page](http://emc.medicines.org.uk/medicine/8320/SPC/COZAAR+12.5+mg%2C+25+mg%2C+50+mg+and+100+mg+Film-Coated+Tablets/#tableOfContents)  4.7 Effects on ability to drive and use machines   |  | No studies on the effects on the ability to drive and use machines have been performed. However, when driving vehicles or operating machinery it must be borne in mind that dizziness or drowsiness may occasionally occur when taking antihypertensive therapy, in particular during initiation of treatment or when the dose is increased. |  | | --- | --- | --- |   [Go to top of the page](http://emc.medicines.org.uk/medicine/8320/SPC/COZAAR+12.5+mg%2C+25+mg%2C+50+mg+and+100+mg+Film-Coated+Tablets/#tableOfContents)  4.8 Undesirable effects   |  | Losartan has been evaluated in clinical studies as follows:  • in controlled clinical trials in approximately 3300 adult patients 18 years of age and older for essential hypertension,  • in a controlled clinical trial in 9193 hypertensive patients 55 to 80 years of age with left ventricular hypertrophy  • in a controlled clinical trial in approximately 3900 patients 20 years of age and older with chronic heart failure  • in a controlled clinical trial in 1513 type 2 diabetic patients 31 years of age and older with proteinuria  • in a controlled clinical trial in 177 hypertensive paediatric patients 6 to 16 years of age  In these clinical trials, the most common adverse reaction was dizziness.  The frequency of adverse reactions listed below is defined using the following convention:  *very common (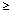 1/10); common (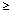 1/100, to < 1/10); uncommon (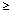 1/1,000, to < 1/100); rare (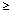 1/10,000,to < 1/1,000); very rare (< 1/10,000), not known (cannot be estimated from the available data).*  Hypertension  In controlled clinical trials of approximately 3300 adult patients 18 years of age and older, for essential hypertension with losartan, the following adverse reactions were reported  *Nervous system disorders:*  common: dizziness, vertigo  uncommon: somnolence, headache, sleep disorders  *Cardiac disorder*:  uncommon: palpitations, angina pectoris  *Vascular disorders:*  uncommon: symptomatic hypotension (especially in patients with intravascular volume depletion, e.g. patients with severe heart failure or under treatment with high dose diuretics), dose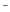related orthostatic effects, rash.  *Gastro-intestinal disorders*:  uncommon: abdominal pain, obstipation  *General disorders and administration site conditions:*  uncommon: asthenia, fatigue, oedema  *Investigations:*  In controlled clinical trials, clinically important changes in standard laboratory parameters were rarely associated with administration of losartan tablets. Elevations of ALT occurred rarely and usually resolved upon discontinuation of therapy. Hyperkalaemia (serum potassium>5.5 mmol/l) occurred in 1.5% of patients in hypertension clinical trials.  Hypertensive patients with left ventricular hypertrophy  In a controlled clinical trial in 9193 hypertensive patients 55 to 80 years of age, with left ventricular hypertrophy, the following adverse reactions were reported:  *Nervous system disorders:*  common: dizziness  *Ear and labyrinth disorders:*  common: vertigo  *General disorders and administration site conditions:*  common: asthenia/fatigue  Chronic heart failure  In a controlled clinical trial in approximately 3900 patients 20 years of age and older, with cardiac insufficiency, the following adverse reactions were reported:  *Nervous system disorders:*  uncommon: dizziness, headache  rare: paraesthesia  *Cardiac disorders:*  rare: syncope, atrial fibrillation, cerebrovascular accident  *Vascular disorders:*  uncommon: hypotension, including orthostatic hypotension  *Respiratory, thoracic and mediastinal disorders:*  uncommon: dyspnoea  *Gastro-intestinal disorders:*  uncommon: diarrhoea, nausea, vomiting  *Skin and subcutaneous tissue disorders:*  uncommon: urticaria, pruritus, rash  *General disorders and administration site conditions:*  uncommon: asthenia/fatigue  *Investigations:*  uncommon: increase in blood urea, serum creatinine and serum potassium has been reported.  Hypertension and type 2 diabetes with renal disease  In a controlled clinical trial in 1513 type 2 diabetic patients 31 years of age and older, with proteinuria (RENAAL study, see section 5.1), the most common drug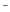related adverse events which were reported for losartan are as follows:  *Nervous system disorders:*  common: dizziness  *Vascular disorders:*  common: hypotension  *General disorders and administration site conditions:*  common: asthenia/fatigue  *Investigations:*  common: hypoglycaemia, hyperkalaemia  The following adverse reactions occurred more often in patients receiving losartan than placebo:  *Blood and lymphatic system disorders:*  not known: anaemia  *Cardiac disorders:*  not known: syncope, palpitations  *Vascular disorders:*  not known: orthostatic hypotension  *Gastro-intestinal disorders:*  not known: diarrhoea  *Musculoskeletal and connective tissue disorders:*  not known: back pain  *Renal and urinary disorders:*  not known: urinary tract infections  *General disorders and administration site conditions:*  not known: flu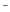like symptoms  *Investigations:*  In a clinical study conducted in type 2 diabetic patients with nephropathy, 9.9% of patients treated with losartan tablets developed hyperkalaemia>5.5 mEq/l and 3.4% of patients treated with placebo.  Post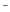marketing experience  The following adverse reactions have been reported in post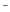marketing experience:  *Blood and lymphatic system disorders:*  not known: anaemia, thrombocytopenia  *Ear and labyrinth disorders:*  not known: tinnitus  *Immune system disorders:*  rare: hypersensitivity: anaphylactic reactions, angio-oedema including swelling of the larynx and glottis causing airway obstruction and/or swelling of the face, lips, pharynx, and/or tongue; in some of these patients angio-oedema had been reported in the past in connection with the administration of other medicines, including ACE inhibitors; vasculitis, including Henoch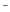Schonlein purpura.  *Nervous system disorders:*  not known: migraine  *Respiratory, thoracic and mediastinal disorders:*  not known: cough  *Gastro-intestinal disorders:*  not known: diarrhoea, pancreatitis  *General disorders and administration site conditions:*  not known: malaise  *Hepatobiliary disorders:*  rare: hepatitis  not known: liver function abnormalities  *Skin and subcutaneous tissue disorders:*  not known: urticaria, pruritus, rash, photosensitivity  *Musculoskeletal and connective tissue disorders:*  not known: myalgia, arthralgia, rhabdomyolysis  *Reproductive system and breast disorders:*  not known: erectile dysfunction/impotence  *Renal and urinary disorders*:  As a consequence of inhibiting the renin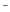angiotensin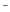aldosterone system, changes in renal function including renal failure have been reported in patients at risk; these changes in renal function may be reversible upon discontinuation of therapy (see section 4.4)  *Psychiatric disorders:*  not known: depression  *Investigations:*  not known: hyponatraemia  Paediatric population  The adverse reaction profile for paediatric patients appears to be similar to that seen in adult patients. Data in the paediatric population are limited. |  | | --- | --- | --- |   [Go to top of the page](http://emc.medicines.org.uk/medicine/8320/SPC/COZAAR+12.5+mg%2C+25+mg%2C+50+mg+and+100+mg+Film-Coated+Tablets/#tableOfContents)  4.9 Overdose   |  | *Symptoms of intoxication*  No case of overdose has been reported. The most likely symptoms, depending on the extent of overdose, are hypotension, tachycardia, possibly bradycardia.  *Treatment of intoxication*  Measures are depending on the time of medicinal product intake and kind and severity of symptoms. Stabilisation of the cardiovascular system should be given priority. After oral intake, the administration of a sufficient dose of activated charcoal is indicated. Afterwards, close monitoring of the vital parameters should be performed. Vital parameters should be corrected if necessary.  Neither losartan nor the active metabolite can be removed by haemodialysis. |  | | --- | --- | --- |   [Go to top of the page](http://emc.medicines.org.uk/medicine/8320/SPC/COZAAR+12.5+mg%2C+25+mg%2C+50+mg+and+100+mg+Film-Coated+Tablets/#tableOfContents)  5. PHARMACOLOGICAL PROPERTIES   |  | Pharmacotherapeutic group: Angiotensin II antagonists, plain ATC code: C09CA01 |  | | --- | --- | --- |   [Go to top of the page](http://emc.medicines.org.uk/medicine/8320/SPC/COZAAR+12.5+mg%2C+25+mg%2C+50+mg+and+100+mg+Film-Coated+Tablets/#tableOfContents)  5.1 Pharmacodynamic properties   |  | Losartan is a synthetic oral angiotensin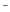II receptor (type AT_1_) antagonist. Angiotensin II, a potent vasoconstrictor, is the primary active hormone of the renin/angiotensin system and an important determinant of the pathophysiology of hypertension. Angiotensin II binds to the AT_1_ receptor found in many tissues (e.g. vascular smooth muscle, adrenal gland, kidneys and the heart) and elicits several important biological actions, including vasoconstriction and the release of aldosterone. Angiotensin II also stimulates smooth muscle cell proliferation.  Losartan selectively blocks the AT_1_ receptor. *In vitro* and *in vivo* losartan and its pharmacologically active carboxylic acid metabolite E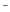3174 block all physiologically relevant actions of angiotensin II, regardless of the source or route of its synthesis.  Losartan does not have an agonist effect nor does it block other hormone receptors or ion channels important in cardiovascular regulation. Furthermore losartan does not inhibit ACE (kininase II), the enzyme that degrades bradykinin. Consequently, there is no potentiation of undesirable bradykinin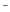mediated effects.  During administration of losartan, removal of the angiotensin II negative feedback on renin secretion leads to increased plasma renin activity (PRA). Increase in the PRA leads to an increase in angiotensin II in plasma. Despite these increases, antihypertensive activity and suppression of plasma aldosterone concentration are maintained, indicating effective angiotensin II receptor blockade. After discontinuation of losartan, PRA and angiotensin II values fell within three days to the baseline values.  Both losartan and its principal active metabolite have a far greater affinity for the AT_1_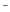receptor than for the AT_2_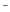receptor. The active metabolite is 10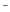 to 40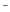 times more active than losartan on a weight for weight basis.  Hypertension studies  In controlled clinical studies, once***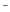***daily administration of losartan to patients with mild to moderate essential hypertension produced statistically significant reductions in systolic and diastolic blood pressure. Measurements of blood pressure 24 hours post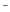dose relative to 5 – 6 hours post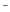dose demonstrated blood pressure reduction over 24 hours; the natural diurnal rhythm was retained. Blood pressure reduction at the end of the dosing interval was 70 – 80 % of the effect seen 5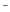6 hours post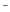dose.  Discontinuation of losartan in hypertensive patients did not result in an abrupt rise in blood pressure (rebound). Despite the marked decrease in blood pressure, losartan had no clinically significant effects on heart rate.  Losartan is equally effective in males and females, and in younger (below the age of 65 years) and older hypertensive patients.  LIFE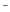study  The Losartan Intervention For Endpoint Reduction in Hypertension [LIFE] study was a randomised, triple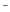blind, active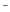controlled study in 9193 hypertensive patients aged 55 to 80 years with ECG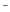documented left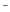ventricular hypertrophy. Patients were randomised to once daily losartan 50 mg or once daily atenolol 50 mg. If goal blood pressure (< 140/90 mmHg) was not reached, hydrochlorothiazide (12.5 mg) was added first and, if needed, the dose of losartan or atenolol was then increased to 100 mg once daily. Other antihypertensives, with the exception of ACE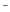inhibitors, angiotensin II antagonists or beta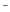blockers were added if necessary to reach the goal blood pressure.  The mean length of follow up was 4.8 years.  The primary endpoint was the composite of cardiovascular morbidity and mortality as measured by a reduction in the combined incidence of cardiovascular death, stroke and myocardial infarction. Blood pressure was significantly lowered to similar levels in the two groups. Treatment with losartan resulted in a 13.0% risk reduction (p=0.021, 95 % confidence interval 0.77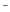0.98) compared with atenolol for patients reaching the primary composite endpoint. This was mainly attributable to a reduction of the incidence of stroke. Treatment with losartan reduced the risk of stroke by 25% relative to atenolol (p=0.001 95% confidence interval 0.63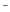0.89). The rates of cardiovascular death and myocardial infarction were not significantly different between the treatment groups.  *Race*  In the LIFE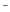Study black patients treated with losartan had a higher risk of suffering the primary combined endpoint, i.e. a cardiovascular event (e.g. cardiac infarction, cardiovascular death) and especially stroke, than the black patients treated with atenolol. Therefore the results observed with losartan in comparison with atenolol in the LIFE study with regard to cardiovascular morbidity/mortality do not apply for black patients with hypertension and left ventricular hypertrophy.  RENAAL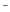study  The Reduction of Endpoints in NIDDM with the Angiotensin II Receptor Antagonist losartan RENAAL study was a controlled clinical study conducted worldwide in 1513 Type 2 diabetic patients with proteinuria, with or without hypertension. 751 patients were treated with losartan. The objective of the study was to demonstrate a nephroprotective effect of losartan potassium over and above the benefit of lowering blood pressure.  Patients with proteinuria and a serum creatinine of 1.3 – 3.0 mg/dl were randomised to receive losartan 50 mg once a day, titrated if necessary, to achieve blood pressure response, or to placebo, on a background of conventional antihypertensive therapy excluding ACE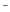inhibitors and angiotensin II antagonists.  Investigators were instructed to titrate the study medication to 100 mg daily as appropriate; 72 % of patients were taking the100 mg daily dose for the majority of the time. Other antihypertensive agents (diuretics, calcium antagonists, alpha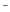 and beta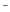receptor blockers and also centrally acting antihypertensives) were permitted as supplementary treatment depending on the requirement in both groups. Patients were followed up for up to 4.6 years (3.4 years on average).  The primary endpoint of the study was a composite endpoint of doubling of the serum creatinine end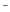stage renal failure (need for dialysis or transplantation) or death.  The results showed that the treatment with losartan (327 events) as compared with placebo (359 events) resulted in a16.1 % risk reduction (p = 0.022) in the number of patients reaching the primary composite endpoint. For the following individual and combined components of the primary endpoint, the results showed a significant risk reduction in the group treated with losartan: 25.3 % risk reduction for doubling of the serum creatinine (p = 0.006); 28.6 % risk reduction for end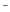stage renal failure (p = 0.002); 19.9 % risk reduction for end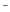stage renal failure or death (p = 0.009); 21.0 % risk reduction for doubling of serum creatinine or end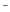stage renal failure (p = 0.01).  All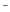cause mortality rate was not significantly different between the two treatment groups. In this study losartan was generally well tolerated, as shown by a therapy discontinuation rate on account of adverse reactions that was comparable to the placebo group.  ELITE I and ELITE II studies  In the ELITE Study carried out over 48 weeks in722 patients with heart failure (NYHA Class II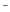IV), no difference was observed between the patients treated with losartan and those treated with captopril with regard to the primary endpoint of a long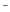term change in renal function. The observation of the ELITE I Study, that, compared with captopril, losartan reduced the mortality risk, was not confirmed in the subsequent ELITE II Study.  In the ELITE II Study losartan 50 mg once daily (starting dose 12.5 mg, increased to 25 mg, then 50 mg once daily) was compared with captopril 50 mg three times daily (starting dose 12.5 mg, increased to 25 mg and then to 50 mg three times daily). The primary endpoint of this prospective study was the all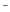cause mortality.  In this study, 3152 patients with heart failure (NYHA Class IIIV) were followed for almost two years (median: 1.5 years) in order to determine whether losartan is superior to captopril in reducing all cause mortality. The primary endpoint did not show any statistically significant difference between losartan and captopril in reducing allcause mortality.  In both comparatorcontrolled (not placebocontrolled) clinical studies on patients with heart failure the tolerability of losartan was superior to that of captopril, measured on the basis of a significantly lower rate of discontinuations of therapy on account of adverse reactions and a significantly lower frequency of cough.  An increased mortality was observed in ELITE II in the small subgroup (22% of all HF patients) taking betablockers at baseline.  Paediatric Population  Paediatric hypertension  The antihypertensive effect of losartan was established in a clinical study involving 177 hypertensive paediatric patients 6 to 16 years of age with a body weight> 20 kg and a glomerular filtration rate> 30 ml/ min/ 1.73 m^2^. Patients who weighed> 20kg to < 50 kg received either 2.5, 25 or 50 mg of losartan daily and patients who weighed> 50 kg received either 5, 50 or 100 mg of losartan daily. At the end of three weeks, losartan administration once daily lowered trough blood pressure in a dose-dependent manner.  Overall, there was a doseresponse. The doseresponse relationship became very obvious in the low dose group compared to the middle dose group (period I: 6.2 mmHg vs. 11.65 mmHg), but was attenuated when comparing the middle dose group with the high dose group (period I: 11.65 mmHg vs. 12.21 mmHg). The lowest doses studied, 2.5 mg and 5 mg, corresponding to an average daily dose of 0.07 mg/ kg, did not appear to offer consistent antihypertensive efficacy.  These results were confirmed during period II of the study where patients were randomised to continue losartan or placebo, after three weeks of treatment. The difference in blood pressure increase as compared to placebo was largest in the middle dose group (6.70 mmHg middle dose vs. 5.38 mmHg high dose). The rise in trough diastolic blood pressure was the same in patients receiving placebo and in those continuing losartan at the lowest dose in each group, again suggesting that the lowest dose in each group did not have significant antihypertensive effect.  Longterm effects of losartan on growth, puberty and general development have not been studied. The longterm efficacy of antihypertensive therapy with losartan in childhood to reduce cardiovascular morbidity and mortality has also not been established.  In hypertensive (N=60) and normotensive (N=246) children with proteinuria, the effect of losartan on proteinuria was evaluated in a 12-week placebo- and active-controlled (amlodipine) clinical study. Proteinuria was defined as urinary protein/creatinine ratio of 0.3. The hypertensive patients (ages 6 through 18 years) were randomised to receive either losartan (n=30) or amlodipine (n=30). The normotensive patients (ages 1 through 18 years) were randomised to receive either losartan (n=122) or placebo (n=124). Losartan was given at doses of 0.7 mg/kg to 1.4 mg/kg (up to maximum dose of 100 mg per day). Amlodipine was given at doses of 0.05 mg/kg to 0.2 mg/kg (up to a maximum dose of 5 mg per day).  Overall, after 12 weeks of treatment, patients receiving losartan experienced a statistically significant reduction from baseline in proteinuria of 36% versus 1% increase in placebo/amlodipine group (p0.001). Hypertensive patients receiving losartan experienced a reduction from baseline proteinuria of -41.5% (95% CI -29.9;-51.1) versus +2.4% (95% CI -22.2;14.1) in the amlodipine group. The decline in both systolic blood pressure and diastolic blood pressure was greater in the losartan group (5.5/-3.8 mmHg) versus the amlodipine group (-0.1/+0.8 mm Hg). In normotensive children a small decrease in blood pressure was observed in the losartan group (-3.7/-3.4 mm Hg) compared to placebo. No significant correlation between the decline in proteinuria and blood pressure was noted, however it is possible that the decline in blood pressure was responsible, in part, for the decline in proteinuria in the losartan treated group. Long-term effects of reduction of proteinuria in children have not been studied. |  | | --- | --- | --- |   [Go to top of the page](http://emc.medicines.org.uk/medicine/8320/SPC/COZAAR+12.5+mg%2C+25+mg%2C+50+mg+and+100+mg+Film-Coated+Tablets/#tableOfContents)  5.2 Pharmacokinetic properties   |  | Absorption  Following oral administration, losartan is well absorbed and undergoes firstpass metabolism, forming an active carboxylic acid metabolite and other inactive metabolites. The systemic bioavailability of losartan tablets is approximately 33%. Mean peak concentrations of losartan and its active metabolite are reached in 1 hour and in 34 hours, respectively.  Distribution  Both losartan and its active metabolite are 99% bound to plasma proteins, primarily albumin. The volume of distribution of losartan is 34 litres.  Biotransformation  About 14% of an intravenously or orallyadministered dose of losartan is converted to its active metabolite. Following oral and intravenous administration of ^14^Clabelled losartan potassium, circulating plasma radioactivity primarily is attributed to losartan and its active metabolite. Minimal conversion of losartan to its active metabolite was seen in about 1% of individuals studied.  In addition to the active metabolite, inactive metabolites are formed.  Elimination  Plasma clearance of losartan and its active metabolite is about 600 ml/min and 50 ml/min, respectively. Renal clearance of losartan and its active metabolite is about 74 ml/min and 26 ml/min, respectively. When losartan is administered orally, about 4% of the dose is excreted unchanged in the urine, and about 6% of the dose is excreted in the urine as active metabolite. The pharmacokinetics of losartan and its active metabolite are linear with oral losartan potassium doses up to 200 mg.  Following oral administration, plasma concentrations of losartan and its active metabolite decline polyexponentially, with a terminal halflife of about 2 hours and 69 hours, respectively. During oncedaily dosing with 100 mg, neither losartan nor its active metabolite accumulates significantly in plasma.  Both biliary and urinary excretions contribute to the elimination of losartan and its metabolites. Following an oral dose/intravenous administration of ^14^Clabelled losartan in man, about 35% / 43% of radioactivity is recovered in the urine and 58%/ 50% in the faeces.  Characteristics in patients  In elderly hypertensive patients the plasma concentrations of losartan and its active metabolite do not differ essentially from those found in young hypertensive patients.  In female hypertensive patients the plasma levels of losartan were up to twice as high as in male hypertensive patients, while the plasma levels of the active metabolite did not differ between men and women.  In patients with mild to moderate alcoholinduced hepatic cirrhosis, the plasma levels of losartan and its active metabolite after oral administration were respectively 5 and 1.7 times higher than in young male volunteers (see section 4.2 and 4.4).  Plasma concentrations of losartan are not altered in patients with a creatinine clearance above 10 ml/minute. Compared to patients with normal renal function, the AUC for losartan is about 2times higher in haemodialysis dialysis patients.  The plasma concentrations of the active metabolite are not altered in patients with renal impairment or in haemodialysis patients.  Neither losartan nor the active metabolite can be removed by haemodialysis.  Pharmacokinetics in paediatric patients  The pharmacokinetics of losartan have been investigated in 50 hypertensive paediatric patients> 1 month to < 16 years of age following once daily oral administration of approximately 0.54 to 0.77 mg/ kg of losartan (mean doses).  The results showed that the active metabolite is formed from losartan in all age groups. The results showed roughly similar pharmacokinetic parameters of losartan following oral administration in infants and toddlers, preschool children, school age children and adolescents. The pharmacokinetic parameters for the metabolite differed to a greater extent between the age groups. When comparing preschool children with adolescents these differences became statistically significant. Exposure in infants/ toddlers was comparatively high. |  | | --- | --- | --- |   [Go to top of the page](http://emc.medicines.org.uk/medicine/8320/SPC/COZAAR+12.5+mg%2C+25+mg%2C+50+mg+and+100+mg+Film-Coated+Tablets/#tableOfContents)  5.3 Preclinical safety data   |  | Preclinical data reveal no special hazard for humans based on conventional studies of general pharmacology, genotoxicity and carcinogenic potential. In repeated dose toxicity studies, the administration of losartan induced a decrease in the red blood cell parameters (erythrocytes, haemoglobin, haematocrit), a rise in ureaN in the serum and occasional rises in serum creatinine, a decrease in heart weight (without a histological correlate) and gastro-intestinal changes (mucous membrane lesions, ulcers, erosions, haemorrhages). Like other substances that directly affect the reninangiotensin system, losartan has been shown to induce adverse effects on the late foetal development, resulting in foetal death and malformations. |  | | --- | --- | --- |   [Go to top of the page](http://emc.medicines.org.uk/medicine/8320/SPC/COZAAR+12.5+mg%2C+25+mg%2C+50+mg+and+100+mg+Film-Coated+Tablets/#tableOfContents)  6. PHARMACEUTICAL PARTICULARS   |  |  |  | | --- | --- | --- |   6.1 List of excipients   |  | microcrystalline cellulose (E460)  lactose monohydrate  pregelatinized maize starch  magnesium stearate (E572)  hydroxypropyl cellulose (E463)  hypromellose (E464)  COZAAR 12.5 mg, 25 mg, 50 mg and 100 mg contain potassium in the following amounts: 1.06 mg (0.027 mEq), 2.12 mg (0.054 mEq), 4.24 mg (0.108 mEq) and 8.48 mg (0.216 mEq) respectively.  COZAAR 12.5 mg tablets also contain carnauba wax (E903), titanium dioxide (E171), indigo carmine (E132) aluminum lake.  COZAAR 25 mg tablets also contain carnauba wax (E903), titanium dioxide (E171).  COZAAR 50 mg tablets also contain carnauba wax (E903), titanium dioxide (E171).  COZAAR 100 mg tablets also contain carnauba wax (E903), titanium dioxide (E171). |  | | --- | --- | --- |   [Go to top of the page](http://emc.medicines.org.uk/medicine/8320/SPC/COZAAR+12.5+mg%2C+25+mg%2C+50+mg+and+100+mg+Film-Coated+Tablets/#tableOfContents)  6.2 Incompatibilities   |  | Not applicable. |  | | --- | --- | --- |   6.3 Shelf life   |  | 3 years |  | | --- | --- | --- |   6.4 Special precautions for storage   |  | Blisters: Store in the original package in order to protect from light and moisture. HDPE Bottle: do not store above 25^o^C. Store in original container. Keep the bottle tightly closed in order to protect from light and moisture. |  | | --- | --- | --- |   [Go to top of the page](http://emc.medicines.org.uk/medicine/8320/SPC/COZAAR+12.5+mg%2C+25+mg%2C+50+mg+and+100+mg+Film-Coated+Tablets/#tableOfContents)  6.5 Nature and contents of container   |  | COZAAR 12.5 mg PVC/PE/PVDC blister packages with aluminum foil lidding in cartons containing 7, 14, 21, 28, 50, 98, 210 or 500 tablets. HDPE bottles of 100 tablets.  COZAAR 25 mg PVC/PE/PVDC blister packages with aluminum foil lidding in cartons containing 7 or 28 tablets.  COZAAR 50 mg PVC/PE/PVDC blister packages with aluminum foil lidding in cartons containing 7, 10, 14, 20, 28, 30, 50, 56, 84, 90, 98, 280 or 500 tablets. HDPE bottles of 100 or 300 tablets.  COZAAR 100 mg - PVC/PE/PVDC blister packages with aluminum foil lidding in cartons containing 7, 10, 14, 15, 20, 28, 30, 50, 56, 84, 90, 98 or 280 tablets. HDPE bottles of 100 tablets.  Not all pack sizes may be marketed. |  | | --- | --- | --- |   [Go to top of the page](http://emc.medicines.org.uk/medicine/8320/SPC/COZAAR+12.5+mg%2C+25+mg%2C+50+mg+and+100+mg+Film-Coated+Tablets/#tableOfContents)  6.6 Special precautions for disposal and other handling   |  | No special requirements. |  | | --- | --- | --- |   [Go to top of the page](http://emc.medicines.org.uk/medicine/8320/SPC/COZAAR+12.5+mg%2C+25+mg%2C+50+mg+and+100+mg+Film-Coated+Tablets/#tableOfContents)  7. MARKETING AUTHORISATION HOLDER   |  | Merck Sharp & Dohme Limited  Hertford Road  Hoddesdon  Herts  EN11 9BU |  | | --- | --- | --- |   [Go to top of the page](http://emc.medicines.org.uk/medicine/8320/SPC/COZAAR+12.5+mg%2C+25+mg%2C+50+mg+and+100+mg+Film-Coated+Tablets/#tableOfContents)  8. MARKETING AUTHORISATION NUMBER(S)   |  | 12.5 mg: PL 0025/0515  25 mg: PL 0025/0336  50 mg: PL 0025/0324  100 mg: PL 0025/0416 |  | | --- | --- | --- |   [Go to top of the page](http://emc.medicines.org.uk/medicine/8320/SPC/COZAAR+12.5+mg%2C+25+mg%2C+50+mg+and+100+mg+Film-Coated+Tablets/#tableOfContents)  9. DATE OF FIRST AUTHORISATION/RENEWAL OF THE AUTHORISATION   |  | 12.5 mg: 6 January 2009  25 mg/50 mg: 15 December 1994  100 mg: 28 November 2001 |  | | --- | --- | --- |   [Go to top of the page](http://emc.medicines.org.uk/medicine/8320/SPC/COZAAR+12.5+mg%2C+25+mg%2C+50+mg+and+100+mg+Film-Coated+Tablets/#tableOfContents)  10. DATE OF REVISION OF THE TEXT   |  | August 2009  **®** denotes registered trademark of E. I. du Pont de Nemours and Company, Wilmington, Delaware, USA.  © Merck Sharp & Dohme Limited 2009. All rights reserved.  SPC.CZR.08.UK.3005-II-003 |  | | --- | --- | --- |   **More information about this product**   - Patient Information Leaflets (PILs):   [COZAAR 12.5mg, 25 mg, 50 mg and 100 mg Film-Coated Tablets](http://emc.medicines.org.uk/medicine/8321/PIL/COZAAR 12.5mg, 25 mg, 50 mg and 100 mg Film-Coated Tablets/)   - Alternative format Patient Information Leaflets (X-PILs):   [COZAAR 12.5mg, 25 mg, 50 mg and 100 mg Film-Coated Tablets](http://xpil.medicines.org.uk/viewpil.aspx?docid=8321)   - Medicine Guides:   [Cozaar](http://medguides.medicines.org.uk/document.aspx?name=Cozaar)  Link to this document from your website: http://emc.medicines.org.uk/medicine/8320/SPC/COZAAR 12.5 mg, 25 mg, 50 mg and 100 mg Film-Coated Tablets/     |  |  |  | | --- | --- | --- | |  | Document Links    [More information  about this product](http://emc.medicines.org.uk/medicine/8320/SPC/COZAAR+12.5+mg%2C+25+mg%2C+50+mg+and+100+mg+Film-Coated+Tablets/#furtherInfo)  [View all medicines  from this company](http://emc.medicines.org.uk/company/86/Merck+Sharp+%26+Dohme+Limited/)  [Print this page](http://emc.medicines.org.uk/printfriendlydocument.aspx?documentid=8320&companyid=86)  [View document history](http://emc.medicines.org.uk/history/8320/SPC/COZAAR+12.5+mg%2C+25+mg%2C+50+mg+and+100+mg+Film-Coated+Tablets) |  | |  |  |  | |  |  |  | |  | Black Triangle   |  | | --- | | This medicine is monitored intensively by the CHM and MHRA | |  | |  |  |  |  |  |  |  | | --- | --- | --- | |  | Legal Categories     |  | POM – Prescription  Only Medicine | | --- | --- | |  | P – Pharmacy | |  | GSL – General Sales List | |  | CE Mark | |  | |  |  |  | |  |  |  | |  | Active Ingredients/Generics     |  | [losartan potassium](http://emc.medicines.org.uk/ingredient/1464/losartan potassium/) | | --- | --- | |  | |  |  |  | |

**APPENDIX TWO - Data Monitoring and Ethics Committee Terms of Reference**

**DATA MONITORING & ETHICS COMMITTEE (DMEC) – ROLES AND RESPONSIBILITIES**

The main features of the DMEC are as follows:

- Access to the unblinded comparative data.
- Monitoring of data and making recommendations to the TSC on whether there are any ethical or safety reasons why the trial should not continue.
- Considering the safety, rights and well-being of the trial participants as paramount.
- Considering whether any interim analysis is necessary, the data from any analysis, requests for its release, and advising the TSC.
- The DMEC may be asked by the TSC, Trial Sponsor or Trial Funder to consider data emerging from other, related studies.
- If funding is required above the level originally requested, the DMEC may be asked by the Chief Investigator, TSC, Trial Sponsor or Trial Funder to provide advice and, where appropriate, information on the data gathered to date, in a way that will not unblind the trial.
- Membership of the DMEC is completely independent[[1]](#footnote-2), small (3‑4 members) and consists of experts in the field eg. a clinician with experience in the relevant area, and expert trial statisticians.
- Responsibility for calling and organising DMEC meetings lies with the Chief Investigator, in association with the Chair of the DMEC. The project team should provide the DMEC with a comprehensive report, the content of which should be agreed in advance by the Chair of the DMEC.
- The DMEC will meet at least annually, and more often as appropriate, and meetings should be timed so that reports can be fed into the TSC.

**APPENDIX THREE – Trial Steering Committee Terms of Reference**

**TRIALS STEERING COMMITTEE (TSC) – ROLES AND RESPONSIBILITIES**

The main features of the TSC are as follows:

- The TSC will provide overall supervision for the trial on behalf of the Trial Sponsor and Trial Funder, and will ensure that the trial is conducted to the rigorous standards set out in the Medical Research Council’s (MRC) Guidelines for Good Clinical Practice. It should be noted that the day‑to‑day management of the trial is the responsibility of the Investigators, the Chief Investigator and the Trial Management Group (TMG) to assist with this function.
- The TSC will concentrate on progress of the trial, adherence to the protocol, patient safety and the consideration of new information of relevance to the research question.
- The TSC will consider that the safety and well-being of the trial participants are the most important considerations, which should prevail over the interests of science and society.
- The TSC will provide advice, via its chair, to the Chief Investigator(s), the Trial Sponsor, the Trial Funder, the Host Institution and the Contractor on all appropriate aspects of the trial.
- Membership of the TSC will be limited and on this occasion includes an independent Chair[[2]](#footnote-3), an independent member, and two consumer representatives. Involvement of these independent members provides protection for both Trial Participants and the Principal Investigator.
- The TSC shall invite representatives of the Trial Sponsor and the Trial Funder to all TSC meetings.
- Responsibility for calling and organising TSC meetings lies with the Chief Investigator. The TSC will meet at least annually, although there may be periods when more frequent meetings are necessary.
- There may be occasions when the Trial Sponsor or the Trial Funder will wish to organise and administer these meetings for particular trials. In the HTA Programme’s case this is unlikely, but it reserves the right to convene a meeting of the TSC in exceptional circumstances.
- The TSC will provide evidence to support any requests for extensions, indicating that all practical steps have been taken to achieve targets.

**APPENDIX FOUR – Questionnaires and Assessment Tools**

**4a: Leisure Time Activity Assessment**

**4b: Chronic Liver Disease Questionnaire (CLDQ)**

**4c: Short Form 36 (SF-36)**

**APPENDIX FIVE – Patient Information Leaflet and Consent Form**

**5a: Patient Information and Consent (Main Study)**

**5b: Patient Information and Consent (Donation of DNA Sample)**

**APPENDIX SIX – Letter to General Practitioner**

**APPENDIX SEVEN – Patient Card**

1. Independence, in respect of the DMEC, is defined as independent from the Chief Investigator, TSC and Host Institution. [↑](#footnote-ref-2)
2. The Good Clinical Practice (GCP) guidelines define independence as: ‘not involved directly in the trial other than as a member of the TSC’. [↑](#footnote-ref-3)
