# Supplementary material for: A randomised controlled trial of losartan as an anti-fibrotic agent in non-alcoholic steatohepatitis
Source: PLoS One. 2017 Apr 18;12(4):e0175717. doi: 10.1371/journal.pone.0175717 (PMC5395178; doi:10.1371/journal.pone.0175717)
Supplement: S3 File — (PDF) [file pone.0175717.s003.pdf]

Newcastle Clinical Trials Unit

End of Trial Report: FELINE Trial

A randomised controlled pilot trial of Losartan as an anti-fibrotic agent in non-alcoholic steatohepatitis

(anti-Fibrotic Effects of Losartan In NASH Evaluation study – FELINE)

Version number: 1.1
Date: 28/02/2017

ISRCTN Number: 57849521

Report prepared by:
Dr Nina Wilkinson (Trial Statistician)
Dr Deborah Stocken (IHS Head of Statistics)
Nicola Goudie (Trial Manager)
Dr Jennifer Wilkinson (Senior Trial Manager)
Professor Elaine McColl (CTU Director)

on behalf of the FELINE TMG:
Chief Investigator: Professor Christopher Day
Co-Investigators: Professor Derek Mann, Dr Stephen Frederick Stewart, Dr Donald Stuart McPherson, Dr Quentin Anstee, Professor Alastair D. Burt, Dr Dina Tiniakos

Contents

1. **Introduction** **3**
   - 1.1 Trial Summary 3
   - 1.2 Trial Objectives 4
   - 1.3 Sample size 5
   - 1.4 Trial diagram/flowchart 6
2. **Timing and reporting interim and final analyses** **6**
3. **Recruitment and randomisation** **7**
   - 3.1 Recruitment 7
   - 3.2 Randomisation 11
   - 3.3 Ineligible Patients 11
4. **Study population** **13**
   - 4.1 Defining populations for analysis 13
   - 4.2 Baseline Patient Characteristics 13
5. **Treatment received** **20**
   - 5.1 Withdrawals 24
6. **Safety analysis** **28**
7. **Outcome data** **33**
   - 7.1 Definition and Calculation of Outcome Measures 33
   - 7.2 Patient follow-up 36
   - 7.3 Analyses of outcome data 37
   - 7.4 Informing future studies 67
   - 7.5 Statistical Software 67
8. **Study Report/Dissemination of results** **67**
9. **Storage and archiving** **67**

1. INTRODUCTION

This end of trial report includes the final analyses and presentation of the FELINE trial. This report, along with all other documents relating to the analysis of this trial, will be stored in the 'Statistical Documentation' of the Trial Master File. This analysis complies with protocol version 10.0.

Trial Status: Closed to follow up

Grant awarded: EME: 10th October 2009, BRC: 16th January 2013

Ethics awarded: Reviewed by Sunderland REC and Favourable Opinion granted23rd October 2010 (REC Ref: 10/H0904/8)

Number of sites: 11

Date 1st site open: December 2010

Date 1st patient randomised: 6th July 2011

Total number of patients randomised: 45

Date last patient randomised: 19th October 2012

Date for last follow up: 24th November 2014

Date of final dataset: 6th October 2015

1.1 Trial Summary

Short title:	FELINE
Chief Investigator:	Professor Christopher Paul Day
Sponsor:	The Newcastle upon Tyne Hospitals NHS Foundation Trust
Funder:	NIHR EME and Newcastle NIHR Biomedical Research Centre Funding
Study design:	Randomised, double-blind, placebo-controlled pilot study
Study Intervention:	Losartan (a dose of 50mg once a day for 24 months) versus matched placebo (1:1 ratio)<br><br>In addition both arms followed instructions from their treating physician in relation to diet, exercise and weight maintenance in compliance with standard care for non-alcoholic steatohepatitis (NASH). This consisted of 150 minutes of exercise per week and 500 kcal reduction in intake per day.
Primary objective:	To determine whether Losartan is effective at slowing down, halting or reversing liver fibrosis in patients with NASH over a two year period.
Secondary objectives:	To determine whether Losartan can prevent clinical deterioration in NASH over a two year period.<br><br>To determine the association between serum, radiological and histological markers in patients with NASH over a two year period.

Study population/size: Target of 214 adult (aged ≥ 18 years) patients (107 per group) with steatohepatitis and fibrosis, resulting from non-alcoholic fatty liver disease.

Study duration: 42 months

Lay Summary of Study

The aim of FELINE was to determine whether Losartan is effective at slowing down, halting or reversing liver fibrosis in patients with non-alcoholic steatohepatitis (NASH). Liver fibrosis is the accumulation of tough, fibrous scar tissue in the liver which occurs in patients with NASH. This resembles alcoholic liver disease, but occurs in people who drink little or no alcohol. The major feature in NASH is fat in the liver, along with inflammation and damage. NASH may be severe and can lead to cirrhosis, in which the liver is permanently damaged and scarred, and no longer able to function properly. In the study, patients aged 18 or over were given either Losartan or placebo. Losartan is a pill used widely in patients with high blood pressure (hypertension), a condition common in NASH. It is also used for the treatment of renal disease in patients with hypertension, in patients with diabetes and in chronic heart failure. Throughout the study, patients underwent assessment of their condition by means of blood samples, liver biopsy and scanning of the liver over a two year period. Some of these procedures were carried out as part of routine clinical care of these patients.

Changes in the project since initial approval

Substantial Amendment 1 – updates required by the MHRA including information regarding concomitant medications and drug interactions to be included in the protocol, addition of further safety biochemistry assessments and addition of a further study visit to ensure participant safety.

Substantial Amendment 2 – amendments to the study schedule and clarification if the number of tablets in each treatment bottle

Substantial Amendment 3 – addition of Glaxo-Smith-Kline biomarker analysis, updates and clarification regarding some study visits, addition of sites and addition of patient invite letter

Substantial Amendment 4 – addition of sites

Substantial Amendment 5 – addition of poster to aid recruitment

Substantial Amendment 6 – addition of sites

Substantial Amendment 7 – change of PI at an existing site

Substantial Amendment 8 – clarification of when local serum samples and GSK Biomarker analysis should be taken, clarification re Fibroscans

Substantial Amendment 9 – addition of sites

Substantial Amendment 10 – change in funding arrangements from NIHR EME to BRC, withdrawal of sites and change in the recruitment end date

Substantial Amendment 11 – update regarding when liver biopsies are required for patients who withdrew early

1.2 Trial Objectives

Primary Clinical Objective

Determine whether Losartan is effective at slowing down halting or reversing liver fibrosis in patients with NASH, assessed by comparing the median rate of change of fibrosis over 24 months. Change in Kleiner score is defined as post biopsy score minus pre biopsy score. A negative change indicates an improvement i.e. the Kleiner stage decreases.

Secondary Clinical Objectives

Determine:

1. Whether Losartan can prevent clinical deterioration in NASH.
2. Whether Losartan can impact on the quality of life in patients with NASH.
3. The association between serum, radiological and histological markers of fibrosis in patients with NASH over a 2 year period.

1.3 Sample size

Study Design and randomisation

Parallel group, double-blind, randomised controlled trial of Losartan versus placebo, with analysis based on the intention to treat. Patients were allocated in a 1:1 ratio. Stratification was by diabetes and centre. Originally the target sample size (calculation in Protocol) was 214 with the expectation that the final outcome would be available from 170 patients.

Early closure of the study

Funding was withdrawn by the EME in January 2013 due to poor recruitment (at that stage 45 patients had been recruited).

Further funding was awarded by the BRC and it was agreed by the management team and regulatory authorities that these 45 patients would be followed up and that the study would have the following revised objectives.

The main aim of the analyses would be to produce interval estimates of parameters of interest.

- Proportion of patients approached that were eligible
- Proportion of eligible patients that agreed to be randomised
- Proportion of patients who received treatment to which they were randomised
- Baseline demographics
- Level of data completeness
- Measures of central tendency and spread for all study outcomes

The intention is to inform the design of future studies.

1.4 Trial diagram/flowchart

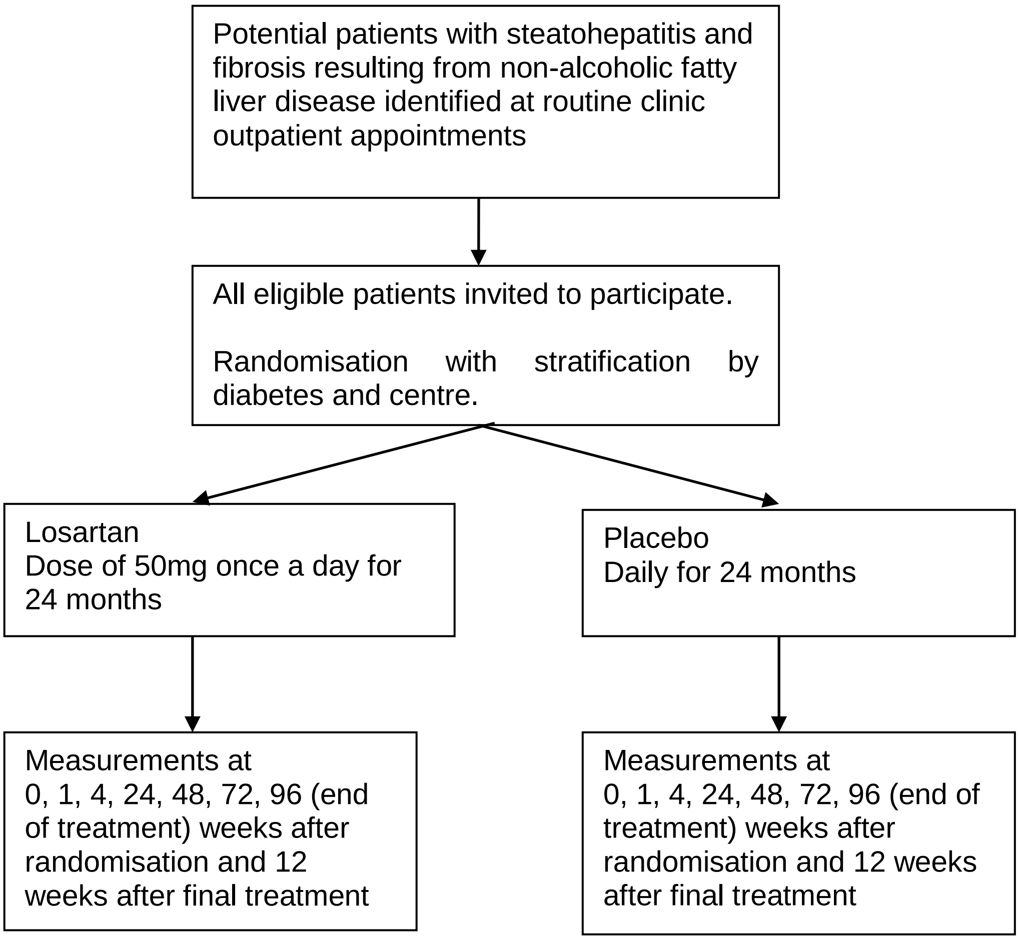

2. TIMING AND REPORTING INTERIM AND FINAL ANALYSES

The Statistical Analysis plan was signed off on 22/9/2015.

Preliminary results of the final outcome data were sent to the CI and co-investigators on 24/9/2015.

The first draft of the end of trial report was presented to the TMG on 2/12/2015.

3. RECRUITMENT AND RANDOMISATION

3.1 Recruitment

- Trial opened to recruitment 30/06/2011 and closed to recruitment 5/10/2012.
- Date of data-lock for treatment and follow-up data: MACRO locked on 12/06/2015.
- Date of data-lock for primary outcome data 23/09/2015.
- Full dataset downloaded from MACRO: 6/10/2015.

Figure 1: Recruitment flow chart based on CONSORT

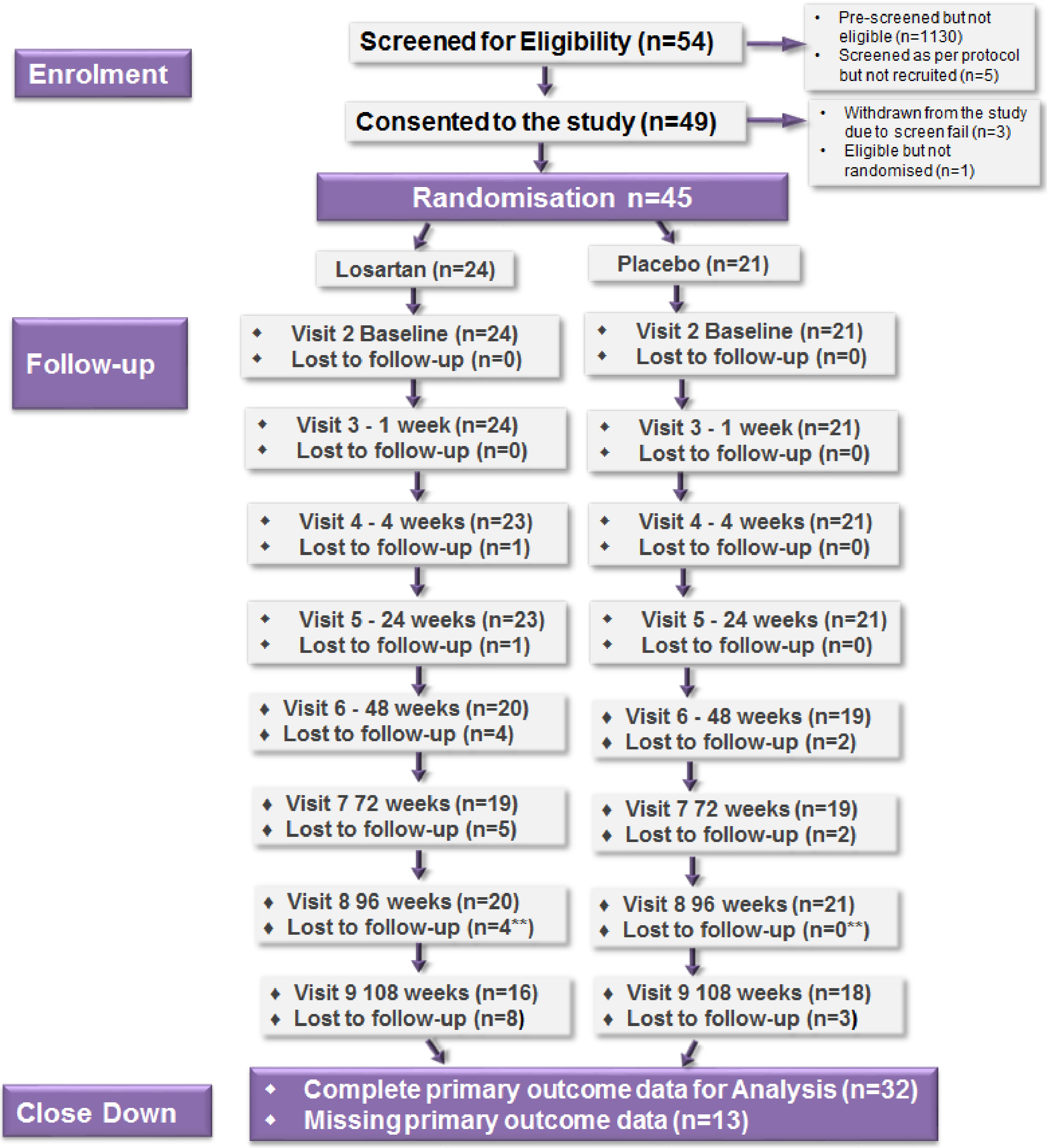

**3 patients attended this visit early after withdrawal at visit 5 – this visit was their end of study visit

Figure 2: Cumulative number of patients randomised by month

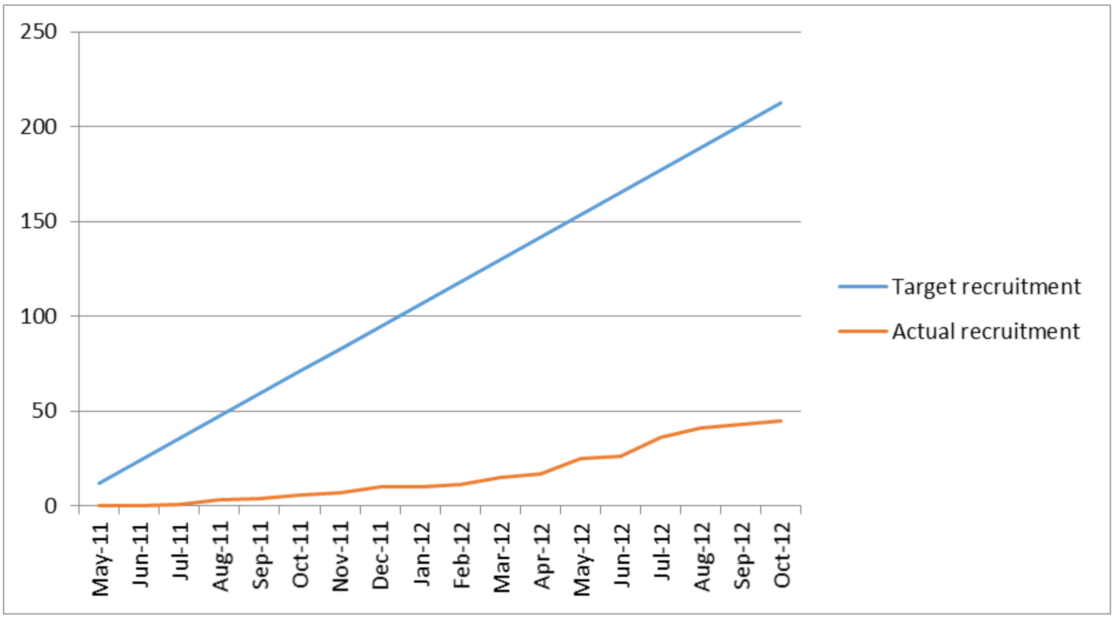

Site recruitment summary

Table 1: Site summary

Number of sites open to recruitment	11
Number of sites who randomised at least one patient	10

Table 2: Cumulative number of sites open by month

Month	Cumulative Number
December 2010	1
January 2011	1
February 2011	1
March 2011	2
April 2011	2
May 2011	2
June 2011 (recruitment commenced)	2
July 2011	2
August 2011	3
September 2011	4
October 2011	6
November 2011	7
December 2011	7
January 2012	7
February 2012	7
March 2012	8
April 2012	9
May 2012	10
June 2012	11
July 2012	11
August 2012	11
September 2012	11
October 2012 (recruitment closed)	11

<table>
<tr><th>Centre</th><th>Randomised</th><th>Screened as per protocol but not recruited</th><th>Patients who were considered but found not to be eligible based on a notes review</th><th>Pre-screened but not eligible</th></tr>
<tr><td>Newcastle</td><td>20</td><td>2 (recruitment had ended + another patient screened but failed to attend for baseline assessment and lost to follow-up)</td><td>32</td><td rowspan="11">ACE inhibitor
Disease too advanced
Biopsy out of date
Malignancy
PBC
ACE inhibitor
Other causes of liver disease</td></tr>
<tr><td>Nottingham</td><td>7</td><td>3 (failed screening)</td><td>N/K</td></tr>
<tr><td>Plymouth</td><td>5</td><td>0</td><td>15</td></tr>
<tr><td>Cambridge</td><td>3</td><td>0</td><td>302</td></tr>
<tr><td>Derby</td><td>3</td><td>0</td><td>3</td></tr>
<tr><td>Imperial</td><td>3</td><td>3 (contra-indicated meds/weight loss)</td><td>10</td></tr>
<tr><td>Birmingham</td><td>1</td><td>1 (recruitment had ended)</td><td>15</td></tr>
<tr><td>GSTT</td><td>1</td><td>0</td><td>600</td></tr>
<tr><td>Liverpool</td><td>1</td><td>0</td><td>85</td></tr>
<tr><td>St George's</td><td>1</td><td>0</td><td>68</td></tr>
<tr><td>Frimley</td><td>0</td><td>0</td><td>N/K</td></tr>
</table>

Park				
**Total**	**45**	**9**	**1130**	

Table 3: Distribution of participants by site and randomised treatment arm

Site	Randomised treatment arm		Total
	Losartan	Placebo	
Newcastle	9	11	20
Nottingham	4	3	7
Plymouth	2	3	5
Cambridge	2	1	3
Derby	1	2	3
Imperial	2	1	3
Birmingham	1	0	1
GSTT	1	0	1
Liverpool	1	0	1
St George's	1	0	1
Frimley Park	0	0	0
**Total**	**24**	**21**	**45**

3.2 Randomisation

- A blocked allocation system was used to allocate patients to the 2 groups (block size was not be disclosed to the investigators), with centre as a stratifying factor. Diabetes is a major risk factor for the progression of fibrosis in NASH. To reduce the risk of unequal proportions of diabetic and non-diabetic patients in active and placebo groups randomisation to Losartan or placebo was also stratified for the presence/ absence of diabetes.
- Number of patients randomised in each treatment arm.

Table 4: Confirm balanced numbers of patients in each treatment group by strata for ITT population

	Losartan	Placebo	All
Diabetes Y	15 (62.50%)	12 (57.14%)	27 (60%)
N	9 (37.50%)	9 (42.86%)	18 (40%)
Total	24 (53.33%)	21 (46.67%)	45

3.3 Ineligible Patients

Despite treatment withdrawal, patients continued to be followed in the study. All statistical analyses was carried out on an intention to treat basis, retaining patients in their randomised treatment groups and including protocol violator and ineligible patients. Ineligible patients were classed as those randomised patients who were found to subsequently not adhere to the eligibility criteria of the trial. The number of ineligible patients and reasons for ineligibility are reported below. Protocol violators were reported as part of treatment compliance (section 5).

Table 5: Reasons of ineligibility for screened patients

Reason	Frequency	Percent
Diagnosed with coronary artery disease and ACE inhibitor prescribed therefore subsequently ineligible	1	33.33%
Cirrhosis discovered on liver biopsy after consented	2	66.67%
Total	3	

Table 6: Reasons for exclusion by patient.

	Reasons
Patient A	5. Are there other causes of chronic liver disease or hepatic steatosis?<br>12. Recent (within 6 months of baseline liver biopsy and screening visit) or concomitant use of agent(s) known to cause hepatic steatosis (corticosteroids, amiodarone, methotrexate, tamoxifen, tetracycline, high dose oestrogens, valproic acid) etc<br>15. Use of ACE inhibitor or ARB in previous year?
Patient B	10. Has there been a recent significant weight loss (>5% total body weight within last 6 months)?<br>12. Recent (within 6 months of baseline liver biopsy and screening visit) or concomitant use of agent(s) known to cause hepatic steatosis (corticosteroids, amiodarone, methotrexate, tamoxifen, tetracycline, high dose oestrogens, valproic acid) etc<br>13. Recent change in anti-diabetic treatment or change in dose or regimen, or concomitant meds etc?<br>15. Use of ACE inhibitor or ARB in previous year?
Patient C	8. Is the proposed participant's alcohol ingestion > 21 units/week (males) or > 14 units/week (females)?<br>15. Use of ACE inhibitor or ARB in previous year?

* Note these are not patient screening numbers, numbered 1-3 to indicate the multiple reasons linked to a particular patient.

There were no ineligible patients that were randomised.

4. STUDY POPULATION

4.1 Defining populations for analysis

1. Intention to treat (ITT) group: all ineligible and protocol violator patients were included in the analysis on an intention to treat basis with patients kept in their randomised treatment groups.
2. Per-treatment group: all randomised patients who start treatment included in the analysis according to the treatment they receive.
3. Kleiner fibrosis stage (KFS) analysis set: all randomised patients who report KFS at within 6 months of baseline and at 96 weeks +/- 7 days.

4.2 Baseline Patient Characteristics

The population is intention to treat.

Demographic and baseline characteristics and trial stratification factors at randomisation were compared across randomisation groups descriptively. Descriptive statistics are tabulated by randomisation group and in total below. Due to the small number of patients median (range) is used rather than mean (sd).

No significance testing was carried out due to the randomised nature of the study.

Demographic and baseline characteristics are in Table 67

Trial factors for comparison included stratification factor diabetes (see section 3.2) and time in days from visit 1 to randomisation.

Table 7: Baseline demographic and clinical characteristics, by randomised treatment arm.

Variable	Losartan n=24	Placebo n=21	Total n=45
Gender n(%)			
Male	13 (54.17%)	12 (57.14%)	25 (55.56%)
Female	11 (45.83%)	9 (42.86%)	20 (44.44%)
Age (years) median (range)	58 (25-75)	45 (21-76)	55 (21-76)
Ethnic group			
White	21 (87.50%)	19 (90.48%)	40 (88.89%)
Other mixed background	1 (4.17%)	0 (0%)	1 (2.22%)
Asian - Indian	1 (4.17%)	0 (0%)	1 (2.22%)
Asian - Pakistani	1 (4.17%)	1 (4.76%)	2 (4.44%)
Chinese	0 (0%)	1 (4.76%)	1 (2.22%)
* From screening			
Weight median (range)	85.1 (74.2-121)	96.7 (61.6-132.5)	93.1 (61.6-132.5)

Height median (range)<br>* From screening	167 (152-183)<br>n=23	171.3 (152-194)<br>n=21	169.1 (152-194)<br>n=44
BMI median (range	32.80 (26.11-43.39)<br>n=23	34.11 (26.46-45.18)<br>n=21	32.93 (26.11-45.18)<br>n=44
Waist circumference median (range)	105.85 (96-126)	111.4 (88-136)	106.5 (88-136)
Systolic BP median (range)	133.5 (109-165)	127 (115-180)	129 (109-180)
Diastolic BP mediar (range)	78.5 (67-95)	81 (70-100)	81 (67-100)
Sitting Heart rate median (range)	75 (59-100)	77 (59-88)	76 (59-100)
Units of alcohol consumed n (%)			
None	13 (54.17%)	9 (42.86%)	22 (48.89%)
1-5 units	7 (29.17%)	7 (33.33%)	14 (31.11%)
6-9 units	0 (0%)	1 (4.76%)	1 (2.22%)
10-15 units	3 (12.50%)	2 (9.52%)	5 (11.11%)
16-19 units	1 (4.17%)	0 (0%)	1 (2.22%)
20-27 units	0 (0%)	1 (4.76%)	1 (2.22%)
≥28 units	0 (0%)	1 (4.76%)	1 (2.22%)
ECG abnormal			
Yes	5 (20.83%)	4 (19.05%)	9 (20%)
No	19 (79.17%)	17 (80.95%)	36 (80%)
* From Screening			
Ultrasound abnormal * Note only 39 had a US so percentages out of 39 for total, 22 for losartan and 17 for placebo.			
Yes	13 (59.09%)	6 (35.29%)	19 (48.72%)
No	5 (22.73%)	4 (23.53%)	9 (23.08%)
Missing	4 (18.18%)	7 (41.18%)	11 (28.21%)

Presence of diabetes (Stratum)<br>Yes<br>No	15 (62.50%)<br>9 (37.50%)	12 (57.14%)<br>9 (42.86%)	27 (60%)<br>18 (40%)
Fibroscan median range<br>Liver stiffness<br>Stiffness median<br>* From screening<br>* n=20 fibroscan done	8.15 (5.2-17.3) n=10<br>8.9 (1.6-26.6) n=6	6.05 (3-11.9) n=6<br>7.95 (7.1-8.8) n=2	7.5 (3-17.3) n=16<br>7.95 (1.6-26.6) n=8
ELF test result median (range)<br>* screening	8.84 (6.54-11.83) n=23	7.96 (6.43-10.28) n=19	8.33 (6.43-11.83) n=42

Physical examination (baseline)

Variable	Losartan n=24	Placebo n=21	Total n=45
CNS			
Normal	22 (91.67%)	19 (90.48%)	41 (91.11%)
Abnormal	1 (4.17%)	0 (0%)	1 (2.22%)
Not examined	0 (0%)	1 (4.76%)	1 (2.22%)
Missing	1 (4.17%)	1 (4.76%)	2 (4.44%)
Neck			
Normal	22 (91.67%)	20 (95.24%)	42 (93.33%)
Abnormal	0 (0%)	0 (0%)	0 (0%)
Not examined	1 (4.17%)	0 (0%)	1 (2.22%)
Missing	1 (4.17%)	1 (4.76%)	2 (4.44%)
HEENT			
Normal	19 (79.17%)	19 (90.48%)	38 (84.44%)
Abnormal	0 (0%)	0 (0%)	0 (0%)
Not examined	4 (16.67%)	1 (4.76%)	5 (11.11%)
Missing	1 (4.17%)	1 (4.76%)	2 (4.44%)
Respiratory			
Normal	23 (95.83%)	20 (95.24%)	43 (95.56%)
Abnormal	0 (0%)	0 (0%)	0 (0%)
Not examined	0 (0%)	0 (0%)	0 (0%)
Missing	1 (4.17%)	1 (4.76%)	2 (4.44%)
Cardiovascular			
Normal	23 (95.83%)	20 (95.24%)	43 (95.56%)

Abnormal	0	(0%)	0	(0%)	0	(0%)
Not examined	0	(0%)	0	(0%)	0	(0%)
Missing	1	(4.17%)	1	(4.76%)	2	(4.44%)
Gastrointestinal						
Normal	23	(95.83%)	18	(85.71%)	41	(91.11%)
Abnormal	0	(0%)	2	(9.52%)	2	(4.44%)
Not examined	0	(0%)	0	(0%)	0	(0%)
Missing	1	(4.17%)	1	(4.76%)	2	(4.44%)
Abdomen						
Normal	21	(87.5%)	18	(85.71%)	39	(86.67%)
Abnormal	2	(8.33%)	2	(9.52%)	4	(8.89%)
Not examined	0	(0%)	0	(0%)	0	(0%)
Missing	1	(4.17%)	1	(4.76%)	2	(4.44%)
Musculoskeletal						
Normal	21	(87.5%)	19	(90.48%)	40	(88.89%)
Abnormal	0	(0%)	0	(0%)	0	(0%)
Not examined	2	(8.33%)	1	(4.76%)	3	(6.67%)
Missing	1	(4.17%)	1	(4.76%)	2	(4.44%)
Endocrine & Metabolic						
Normal	22	(91.67%)	19	(90.48%)	41	(91.11%)
Abnormal	0	(0%)	0	(0%)	0	(0%)
Not examined	1	(4.17%)	1	(4.76%)	2	(4.44%)
Missing	1	(4.17%)	1	(4.76%)	2	(4.44%)
Hematopoietic/Lymphatic						
Normal	20	(83.33%)	19	(90.48%)	39	(86.67%)
Abnormal	0	(0%)	0	(0%)	0	(0%)
Not examined	3	(12.5%)	1	(4.76%)	4	(8.89%)
Missing	1	(4.17%)	1	(4.76%)	2	(4.44%)
Neurological						
Normal	21	(87.5%)	20	(95.24%)	41	(91.11%)
Abnormal	1	(4.17%)	0	(0%)	1	(2.22%)
Not examined	1	(4.17%)	0	(0%)	1	(2.22%)
Missing	1	(4.17%)	1	(4.76%)	2	(4.44%)
Dermatological						
Normal	19	(79.17%)	19	(90.48%)	38	(84.44%)
Abnormal	2	(8.33%)	1	(4.76%)	3	(6.67%)
Not examined	2	(8.33%)	0	(0%)	2	(4.44%)
Missing	1	(4.17%)	1	(4.76%)	2	(4.44%)
Psychiatric/Psychological						
Normal	19	(79.17%)	18	(85.71%)	37	(82.22%)
Abnormal	0	(0%)	0	(0%)	0	(0%)
Not examined	4	(16.67%)	2	(9.52%)	6	(13.33%)
Missing	1	(4.17%)	1	(4.76%)	2	(4.44%)

Physical examination results (screening pre-baseline)

Variable	Losartan n=24		Placebo n=21		Total n=45	
CNS						
Normal	22	(91.67%)	20	(95.24%)	42	(93.33%)
Abnormal	2	(8.33%)	0	(0%)	0	(0%)
Not examined	0	(0%)	1	(4.76%)	3	(6.67%)
Neck						
Normal	21	(87.50%)	20	(95.24%)	41	(91.11%)
Abnormal	0	(0%)	0	(0%)	0	(0%)
Not examined	3	(12.50%)	1	(4.76%)	4	(8.89%)
HEENT						
Normal	21	(87.50%)	20	(95.24%)	41	(91.11%)
Abnormal	0	(0%)	0	(0%)	0	(0%)
Not examined	3	(12.50%)	1	(4.76%)	4	(8.89%)
Respiratory						
Normal	22	(91.67%)	20	(95.24%)	42	(93.33%)
Abnormal	1	(4.17%)	0	(0%)	1	(2.22%)
Not examined	1	(4.17%)	1	(4.76%)	2	(4.44%)
Cardiovascular						
Normal	23	(95.83%)	20	(95.24%)	43	(95.56%)
Abnormal	0	(0%)	0	(0%)	0	(0%)
Not examined	1	(4.17%)	1	(4.76%)	2	(4.44%)
Gastrointestinal						
Normal	23	(95.83%)	19	(90.48%)	42	(93.33%)
Abnormal	0	(0%)	1	(4.76%)	1	(2.22%)
Not examined	1	(4.17%)	1	(4.76%)	2	(4.44%)
Abdomen						
Normal	22	(91.67%)	20	(95.24%)	42	(93.33%)
Abnormal	1	(4.17%)	0	(0%)	1	(2.22%)
Not examined	1	(4.17%)	1	(4.76%)	2	(4.44%)
Musculoskeletal						
Normal	19	(79.17%)	20	(95.24%)	39	(86.67%)
Abnormal	1	(4.17%)	0	(0%)	1	(2.22%)
Not examined	4	(16.67%)	1	(4.76%)	5	(11.11%)
Endocrine & Metabolic						
Normal	21	(87.50%)	19	(90.48%)	40	(88.89%)
Abnormal	0	(0%)	1	(4.76%)	1	(2.22%)
Not examined	3	(12.50%)	1	(4.76%)	4	(8.89%)
Hematopoietic/Lymphatic						
Normal	19	(79.17%)	20	(95.24%)	39	(86.67%)
Abnormal	0	(0%)	0	(0%)	0	(0%)
Not examined	5	(20.83%)	1	(4.76%)	6	(13.33%)
Neurological						
Normal	20	(83.33%)	20	(95.24%)	40	(88.89%)

Abnormal	1 (4.17%)	0 (0%)	1 (2.22%)
Not examined	3 (12.50%)	1 (4.76%)	4 (8.89%)
Dermatological			
Normal	19 (79.17%)	16 (76.19%)	35 (77.78%)
Abnormal	3 (12.50%)	3 (14.29%)	6 (13.33%)
Not examined	2 (8.33%)	2 (9.52%)	4 (8.89%)
Psychiatric/Psychological			
Normal	20 (83.33%)	20 (95.24%)	40 (88.89%)
Abnormal	0 (0%)	0 (0%)	0 (0%)
Not examined	4 (16.67%)	1 (4.76%)	5 (11.11%)

Biochemistry results (baseline)

**Variable**	**Losartan** n=24	**Placebo** n=21	**Total** n=45
Sodium median (range)	139.5 (135-143)	141 (136-145)	140 (135-145)
Potassium median (range)	4.35 (3.8-4.8)	4.3 (3.7-4.8)	4.3 (3.7-4.8)
Urea median (range)	4.9 (3.2-7.9) n=23	5 (3.3-6.9)	4.95 (3.2-7.9) n=44
Glucose median (range)	5.95 (4.4-17.1)	6.2 (3.6-15.9) n=20	6 (3.6-17.1) n=44
AST median (range)	35 (14-102) n=21	46 (30-70) n=18	43 (14-102) n=39
ALT median (range)	52.5 (21-136)	65 (33-135)	59 (21-136)
ALP median (range)	89.5 (44-173)	72 (49-116)	84 (44-173)
Creatinine median (range)	75.5 (48-105)	72 (5-97)	74 (5-105)
Bilirubin median (range)	10 (5-25) n=24	10 (4-45) n=20	10 (4-45) n=44
Albumin median (range)	44.5 (34-50)	46 (35-75)	45 (34-75)
Triglyceride median (range)	1.7 (0.9-7.9) n=23	2 (0.4-4.4) n=19	1.85 (0.4-7.9) n=42
HDL Cholesterol median (range)	1.1 (0.7-2.8) n=22	1.1 (0.8-3.5) n=20	1.1 (0.7-3.5) n=42
Total Cholesterol median (range)	4.3 (2.1-7.5) n=23	4.6 (1-6.5) n=20	4.3 (1-7.5) n=43
LDL Cholesterol	2.5 (0.8-3.8) n=19	3.2 (1.2-4.4) n=18	3 (0.8-4.4) n=37
Gamma GT median (range)	70 (18-355)	62 (23-256)	64 (18-355)

Biochemistry results (screening pre-baseline)

**Variable**	**Losartan** n=24	**Placebo** n=21	**Total** n=45

Sodium median (range)	140 (136-144) n=24	140 (133-146) n=21	140 (133-146) n=45
Potassium median (range)	4.3 (3.4-4.6) n=24	4.2 (3.6-5) n=21	4.3 (3.4-5) n=45
Urea median (range)	4.8 (0-8.1) n=23	4.9 (3.5-6.6) n=21	4.8 (0-8.1) n=44
Glucose median (range)	6.1 (4.6-12.2) n=23	5.9 (0.5-14.8) n=21	5.95 (0.5-14.8) n=44
AST median (range)	42.5 (19-147) n=22	43 (29-64) n=19	43 (19-147) n=41
ALT median (range)	55.5 (22-154) n=24	57 (31-99) n=21	57 (22-154) n=45
ALP median (range)	87 (40-193) n=24	83 (48-116) n=21	86 (40-193) n=45
Creatinine median (range)	75 (51-103) n=24	69 (58-135) n=21	72 (51-135) n=45
Bilirubin median (range)	9.5 (6-33) n=24	10 (5-46) n=21	10 (5-46) n=45
Albumin median (range)	44 (36-53) n=24	45 (38-50) n=21	45 (36-53) n=45
Triglyceride median (range)	1.7 (0.7-6.4) n=23	1.7 (0.4-5.1) n=21	1.7 (0.4-6.4) n=44
HDL Cholesterol median (range)	1.1 (0.7-2.7) n=22	1.1 (0.8-3.4) n=20	1.1 (0.7-3.4) n=42
Total Cholesterol median (range)	4.65 (1.9-7) n=24	4.3 (2.3-44) n=21	4.5 (1.9-44) n=45
LDL Cholesterol	2.7 (0.8-4.1) n=20	3 (1-23) n=19	2.8 (0.8-23) n=39
Gamma GT median (range)	73.5 (22-359) n=24	68 (24-265) N=21	71 (22-359) n=45

Haematology Results (baseline)

**Variable**	**Losartan** **n=24**	**Placebo** **n=21**	**Total** **n=45**
Haemoglobin median (range)	14.6 (12.3-142) n=23	14.9 (13.2-18)	14.85 (12.3-142) n=44
Leukocytes WBC median (range)	7.2 (4.3-13.1) n=23	7.3 (4.4-12.3)	7.25 (4.3-13.1) n=44
Platelets median (range)	224 (137-360) n=23	224 (158-404)	224 (137-404) n=44
MCV median (range)	89.55 (76.9-99) n=22	87.3 (80.2-94.3)	89 (76.9-99) n=43
HBA1C mmol median (range)	53.5 (34-81) n=22	42 (31-81)	46 (31-81) n=43
Prothrombus Time median (range)	11 (1-15) n=23	11 (10-22) n=20	11 (1-22) n=43

Haematology Results (screening pre-baseline)

Variable	Losartan<br>n=24	Placebo<br>n=21	Total<br>n=45
Haemoglobin median (range)	14.8 (12.5-140)<br>n=24	15.1 (13.1-17.4)<br>n=21	15.1 (12.5-140)<br>n=45
Leukocytes WBC median (range)	7.2 (4.4-14.7)<br>n=24	7.3 (4.4-12.2)<br>n=21	7.3 (4.4-14.7)<br>n=45
Platelets median (range)	225.5 (129-341)<br>n=24	221 (147-416)<br>n=21	221 (129-416)<br>n=45
MCV median (range)	89.85 (79.3-100)<br>n=24	87.5 (79.5-93.7)<br>n=21	89.3 (79.3-100)<br>n=45
HBA1C mmol median (range)	53 (53-53)<br>n=1	48 (41-50)<br>n=3	49 (41-53)<br>n=4
Prothrombus Time median (range)	10.5 (1-15)<br>n=22	11 (9-13)<br>n=21	11 (1-15)<br>n=43

Laboratory results – Other blood samples (Baseline)

Variable	Losartan<br>n=24	Placebo<br>n=21	Total<br>n=45
Apolopoproteins median (range)	1.35 (1-2)<br>n=16	1.35 (1-1.7)<br>n=16	1.35 (1-2)<br>n=32
Alpha-2-macroglobulin	1.4 (0-2.5)<br>n=10	1.9 (1.2-4.2)<br>n=11	1.4 (0-4.2)<br>n=21
Haptoglobins	1.9 (0.6-797)<br>n=18	2 (0.7-1651)<br>n=17	2 (0.6-1651)<br>n=35
IgG	10.4 (6.7-15.1)<br>n=21	10.8 (6.6-15)<br>n=21	10.45 (6.6-15.1)<br>n=42
IgA	2.9 (0.87-5.08)<br>n=21	2.64 (1-6.3)<br>n=21	2.715 (0.87-6.3)<br>n=42
IgM	1.09 (0.26-2.75)<br>n=21	1.02 (0.25-7)<br>n=21	1.04 (0.25-7)<br>n=42
Insulin	22.2 (9.7-81.4)<br>n=15	23.8 (4.4-57.6)<br>n=15	22.35 (4.4-81.4)<br>n=30
C-peptide	2.31 (0.68-4138)<br>n=15	2.22 (0.16-3054)<br>n=17	2.26 (0.16-4138)<br>n=32

5. TREATMENT RECEIVED

Please note that some of these data were not in an analysable format – mainly due to repeated information and inconsistent location of data entry, for example site staff entering

tablets returned to V7 in V6 or V7 or both. In order to use the data, multiple data manipulations and assumptions have been made, as documented. The main assumptions were:

- repeated information could be deleted
- days from randomisation to date of return could be used to label the return date with a particular visit
- date of return could be used to match to date of dispense – i.e. if the drug was returned at a date after V5 dispense but before V6 dispense it corresponded to the V5 dispensed tablets
- the year was changed in many fields on the assumption that the month and day matched the visit number
- If a patient returned 0 tablets between visits, but with visit information then this was ignored.
- If two different numbers of tablets were entered for the same day, then a mean was calculated.
- Withdrawals after baseline took treatment until withdrawal date.

Hard coding was required so this analysis should be treated with caution.
Population for analysis is the per treatment group.

24 randomised to Losartan, 21 randomised to Placebo.
24 start treatment with Losartan, 21 start treatment with Placebo.

The active arm receive a 50mg dose of Losartan once a day for 24 months. The control arm receive a matched placebo. The treatment is blinded.

If a patient continued in the study for the scheduled 24 months they should have taken 96 weeks of medication and therefore 96*7=672 capsules of Losartan or placebo. The study medication was prescribed according to the following schedule, to allow for slippage on treatment visit dates:

Dispensing 1, Visit 2: (baseline) – 37 capsules to last 4 weeks – dose 1
Dispensing 2, Visit 4: (4 weeks) – 148 capsules to last 20 weeks – dose 2
Dispensing 3, Visit 5: (24 weeks) – 185 capsules to last 24 weeks – dose 3
Dispensing 4, Visit 6: (48 weeks) – 185 capsules to last 24 weeks – dose 4
Dispensing 5, Visit 7: (72 weeks) – 185 capsules to last 24 weeks – dose 5

Treatment ends at Visit 8 (96 weeks).

Note that more pills are prescribed than required to allow for patients who do not return at exactly the right time, for example at 5 weeks rather than 4 weeks.

We define actual dose taken as the number of pills dispensed minus the number of pills returned. If the number of tablets returned is zero we assume a true zero and that the data are not missing.

We define expected dose as the date of clinic minus date of drug being dispensed at the previous visit. Therefore the number of days corresponds to the number of pills expected to be taken.

The number of patients receiving the full treatment schedule is reported based on attendance to a visit and no withdrawal from study drug.

The number of patients who did not receive the full treatment schedule is also reported.
The time from randomisation to first treatment is reported for each treatment group.

Table 8: Dosing schedule of those starting treatment – time on treatment is based on final visit date/return date minus BL date.

	**Dispensings received**	**Losartan n (% of randomised)**	**Placebo n (% of randomised)**
	1-2-3-4-5	19 (79.17%)	18 (85.71%)
	1-2-3-4-*	20 (83.33%)	18 (85.71%)
	1-2-3-*-*	23 (95.83%)	20 (95.24%)
	1-2-*-*-*	23 (95.83%)	20 (95.24%)
	1-*-*-*-*	24 (100%)	21 (100%)
Time on treatment (days): N<br>Median<br>IQR<br>Range		24<br>667.5<br>(362-693)<br>(7-764)	21<br>672<br>(511-700)<br>(28-792)
Time from randomised to treatment (days): N<br>Median<br>IQR<br>Range		24<br>0<br>(0,0)<br>(-5,3)	21<br>0<br>(0,0)<br>(0,9)

Table 9: Dosage summary

	Dispensing 1	Dispensing 2	Dispensing 3	Dispensing 4	Dispensing 5
Dose taken (number of pills)	29 (7-37)<br>n=13	143.5 (0-185)<br>n=34	164 (18-185)<br>n=33	163 (107-185)<br>n=27	164.5 (23-185)<br>n=24
Percentage protocol dose	100 (89.29-127.59)<br>n=11	100 (0-135.04)<br>n=33	96.74 (17.77-176.19)<br>n=32	95.64 (68.57-103.43)<br>n=26	93.10 (11.86-106.32)<br>n=24
Timing from randomisation to tablets dispensed	0 (0-9)<br>n=44	29 (20-59)<br>n=40	168 (140-198)<br>n=38	336 (308-371)<br>n=33	510 (476-610)<br>n=30

The number of protocol violators and reasons for violation were reported.

Table 10: Listing of protocol deviations by date of deviation.

Site	Date of Deviation/Breach	Description
Birmingham	13/03/2014	Patient 11101<br>PT in seconds and LDL cholesterol not done at Visit 8
Birmingham	13/03/2014	Patient 11101<br>Drug administered in error at Visit 8 – 2 tablets were taken by the participant before the error was discovered the day after the visit 8 appointment
Birmingham	10/05/2012	Patient 11101<br>Pax Gene samples not taken in error at Visit 2
	25/10/2012	Patient 11101<br>Pax Gene samples not taken in error at Visit 5
	11/04/2013	Patient 11101<br>Pax Gene samples not taken in error at Visit 6
Birmingham	10/05/2012	Patient 11101<br>Plasma samples not collected at visit 2 – missed in error
	14/06/2012	Patient 11101<br>Plasma samples not collected at visit 4 – missed in error
	25/10/2012	Patient 11101<br>Plasma samples not collected at visit 5 – missed in error
	11/04/2013	Patient 11101<br>Plasma samples not collected at visit 6 – missed in error
	26/09/2013	Patient 11101<br>Plasma samples not collected at visit 7 – missed in error
	13/03/2014	Patient 11101<br>Plasma samples not collected at visit 8 – missed in error
Newcastle	11/10/2013	Patient 15109<br>Discovered patient taking expired visit 6 medication - discovered by site pharmacy staff. Visit 6 medication was dispensed before the new supply of medication was received – the expiry date was 30/09/2013 – patient will therefore have been medication a few days past its expiry date.
	11/10/2013	Patient 15205<br>Discovered patient taking expired visit 6 medication - discovered by site pharmacy staff. Visit 6 medication was dispensed before the new supply of medication was received – the expiry date was 30/09/2013 – patient will therefore have been medication a few days past its expiry date.

	30/10/2013	Patient 15204<br>Discovered this patient was also taking expired medication during a subsequent monitoring visit.
	30/10/2013	Patient 15104<br>Discovered this patient was also taking expired medication during a subsequent monitoring visit.
Nottingham	23/10/2013	Patient 16201<br>Discovered that patient had taken expired medication between 01/10/2013 and the next study visit on 23/10/2013
Nottingham	27/05/2012	Temperature deviation within pharmacy – temperature exceed 25 degrees
	26/04/2013	Temperature deviation within pharmacy – temperature exceed 25 degrees
Plymouth	03/12/2013	Patient 17101<br>Discovered that patient had taken expired medication between 01/10/2013 and 20/10/2013
St George's	30/09/2013	Temperature deviation within pharmacy – temperature exceed 25 degrees

No significance testing was carried out.

5.1 Withdrawals

A patient could
- withdraw from study drug but carry on with study visits
- withdraw from study drug and study visits but consent to the collection of routine protocol data
- completely withdraw and have no further data collected

Summary of withdrawals

Reason	n	Percent
Lost to follow-up	4	50%
Suffered headaches and wanted to discontinue study treatment	1	12.5%
Family planning reasons	2	25%
Consented but changed mind after re-reading study info	1	12.5%
Total	8	

Patient study ID	Randomisation date	Withdrawal date	Reason	Level of withdrawal
Cambridge (12101)	30/05/12	06/06/12	Patient did not return after visit 3.	3
Cambridge (12201)	24/09/12	06/03/13	Diagnosed with coronary artery disease. Prescribed ACE inhibitor.	3
Newcastle (15102)	26/10/11	23/11/11	Patient suffered from headaches and wished to discontinue with medication. Follow-ups completed as per study schedule.	1
Newcastle (15111)	18/07/12	November 2013	Family planning reasons.	1 (originally patient was going to continue with follow-ups but did not attend last visit in December 2013. Attempts to contact patient have failed).
Nottingham (16204)	16/07/12	17/06/13	Withdrawn completely. After reading updated information sheet, patient decided to withdraw.	3
Plymouth (17204)	19/07/12	01/07/13	Family planning reasons.	3
Plymouth (17201)	27/03/12	06/11/12	Later discovered that there was cirrhosis on liver biopsy. Not eligible.	3
Plymouth (17202)	26/04/12	05/11/12	Later discovered that there was cirrhosis on liver biopsy. Not eligible.	3

Table 11: Withdrawals by randomised treatment arm and date of withdrawal

Losartan			Placebo		
**Date of withdrawal**	**Time of withdrawal from date of randomisation**	**Reason**	**Date of withdrawal**	**Time of withdrawal from date of randomisation**	**Reason**
Date	n days	Text – line listing	Date	n days	Text – line listing
06/06/12	7	Patient did not return after visit 3.	23/11/11	28	Patient suffered from headaches and wished to discontinue with medication. Follow-ups completed as per study schedule.
06/03/13	163	Diagnosed with coronary artery disease. Prescribed ACE inhibitor.	17/06/13	336	Withdrawn completely. After reading updated information sheet, patient decided to withdraw.
November 2013	471	Family planning reasons.	05/11/12	193	Later discovered that there was cirrhosis on liver biopsy. Not eligible.
01/07/13	347	Family planning reasons			

06/11/12	224	Later discovered that there was cirrhosis on liver biopsy. Not eligible.			

Table 12: Time of withdrawals by randomised treatment arm

	**Losartan n=5**	**Placebo n=3**
Time of withdrawal from randomisation	Median 224<br>IQR 163-347<br>Range 7-471	Median 193<br>IQR 110.5-264.5<br>Range 28-336

6. SAFETY ANALYSIS

Most adverse events (AEs) and adverse reactions (ARs) were expected to be treatment related toxicities due to the drugs used in the study, for example dizziness, vertigo, hypotension, asthenia/fatigue, hypoglycaemia and hyperkalaemia. For a full list please see appendix 1 of the protocol.

AEs were graded according to a three-point scale (mild, moderate, severe). AEs were classified as related (definitely, probable, possible) or unrelated (unlikely, unrelated).

Serious adverse events (SAEs) or serious adverse reaction (SARs) are any untoward medical occurrence or effect that at any dose

- Results in death
- Is life-threatening (refers to an event in which the subject was at risk of death at the time of the event; it does not refer to an event which hypothetically might have caused death if it were more severe)
- Requires hospitalisation, or prolongation of existing hospitalisation
- Results in persistent or significant disability or incapacity
- Is a congenital anomaly or birth defect

An AE may be severe but not serious.

We provided a summary which includes:

- Number of subjects evaluable for AEs
- Number of AEs reported during study
- Number of severe, moderate and mild AEs reported during study as a percentage of number of AEs reported during study
- Number of subjects with AEs as a percentage of number of subjects evaluable for AEs
- Number of subjects with SAEs as a percentage of number of subjects evaluable for AEs
- Number of subjects with severe, moderate and mild AEs as a percentage of number of subjects evaluable for AEs

The above are provided overall and by treatment arm with the ITT population. SAEs are provided for the ITT population.

No significance testing was carried out.

Table 13 (a): Reported AEs (not including SAEs) on ITT population

**Related to treatment**	**Severity**	**Losartan**<br>**n(%)**	**Placebo**<br>**N(%)**
**Not related**	**Mild**	43 (51.19%)	56 (76.71%)
	**Moderate**	34 (40.48%)	16 (21.92%)
	**Severe**	7 (8.33%)	0 (0%)
	**Missing**	0 (0%)	1 (1.37%)
	**Total**	**84**	**73**
**Possibly related**	**Mild**	9 (60%)	24 (92.31%)
	**Moderate**	6 (40%)	1 (3.85%)

	**Severe**	0 (0%)	1 (3.85%)
	**Missing**	0 (0%)	0 (0%)
	**Total**	**15**	**26**
**Probably related**	**Mild**	1 (100%)	0 (0%)
	**Moderate**	0 (0%)	0 (0%)
	**Severe**	0 (0%)	0 (0%)
	**Missing**	0 (0%)	0 (0%)
	**Total**	**1**	**0**
**Missing**	**Mild**	0 (0%)	2 (100%)
	**Moderate**	0 (0%)	0 (0%)
	**Severe**	0 (0%)	0 (0%)
	**Missing**	0 (0%)	0 (0%)
	**Total**	**0**	**2**
**Arm Totals**		**100**	**101**

Table 13 (b): Tabulation of categorised AEs

**Category**	**Number of unique patients placebo**	**Number of AEs Placebo arm**		**Number of unique patients losartan**	**Number of AEs Losartan arm**
ankle swelling	1	2		0	0
anxiety	1	1		0	0
asthma	1	1		0	0
axilla abscess	0	0		1	1
back pain	1	1		0	0
cellulitis	0	0		1	1
cervical spondylosis	0	0		1	1
chest pain	0	0		1	1
colonoscopy	1	1		0	0
constipation	0	0		1	1
cough	1	1		3	4
depression	0	0		1	1
diarrhoea	2	2		2	2
diarrhoea and vomiting	0	0		1	1
dislocated left knee cap	1	1		0	0
dizzyness	6	6		4	5
dorsal capsulotomy of mcp (metacapophalangeal) joints in right hand	0	0		1	1
dry mouth	0	0		1	2

dyslipidaemia	0	0		2	2
dysmenorrhoea	1	1		0	0
dyspepsia	2	2		2	2
dysphagia	0	0		1	1
dysuria	1	1		0	0
ear infection	0	0		1	1
elective removal of screw from left ankle	0	0		1	1
elective surgery for cystoscopy and bladder biopsy	0	0		1	1
epistaxis	1	1		1	1
eructation	1	1		0	0
excision scalp lipoma	0	0		1	1
extreme coldness in extremities-especially hand and feet.	1	1		0	0
falls	0	0		1	1
fatigue	2	3		1	2
fibromyalgia	0	0		1	1
generalized stabbing pain and ache from left ankle up to the left hip.	0	0		1	1
gout	1	2		0	0
haematuria	0	0		1	2
headaches	6	14		2	2
herniated lumbar disc	1	1		0	0
hot and cold sweats	1	1		0	0
hyperglycaemia	4	6		1	1
hypertension	2	2		2	2
hysterectomy	0	0		1	1
influenza	2	2		0	0
insomnia	0	0		1	1
irritable bowel syndrone	1	1		1	1
kidney stone.	0	0		1	1
left ankle pain	0	0		1	1
left knee pain	0	0		1	1
left lower leg - hot tender and tight at night	1	1		0	0
left shoulder pain.	0	0		1	1
leg cramps	0	0		1	1
leg pain	1	1		1	1
light headed	1	4		0	0
loin pain	1	1		0	0
lower respiratory tract infection	4	5		3	4
malaise	1	1		0	0

memory impairment	0	0		2	3
migraine	1	2		0	0
muscle spasm	0	0		1	1
myalgia	2	2		2	2
nausea	3	6		2	3
oesteoarthritis both knees	0	0		1	1
pain in left ankle	0	0		1	1
pain in tongue when eating or drinking since taking 1 week of antibiotics.	0	0		1	1
painful eye after laser eye treatment	1	1		0	0
painful wisdom tooth.	1	1		0	0
patella ligament injury	1	1		0	0
palpitations	1	1		0	0
parasthesia	1	1		1	2
post liver biopsy pain	1	2		2	4
pruritis	0	0		3	6
rectal polyps removed	0	0		1	1
renal colic	0	0		1	1
right knee pain	1	1		0	0
scratch on left cornea	1	2		0	0
severe trigonitis	0	0		1	1
shoulder pain	0	0		1	1
skin erythema	0	0		1	1
skin infection	1	1		1	1
skin rash	1	1		0	0
sleep apnoea	0	0		1	1
soft tissue facial injuries	0	0		1	1
sore throat	1	1		1	1
tonsillitis	1	1		1	1
toothache and subsequent tooth extraction	0	0		1	1
viral illness	0	0		1	1
viral upper respiratory infection	3	6		4	5
vitamin D deficiency	1	1		0	0
vomiting	1	1		1	2
whiplash following rta	0	0		1	1
whiplash/back pain	1	1		0	0
**Total**		**101**			**100**

Table 14 (a): Reported SAEs on ITT population

**Related to treatment**	**Severity**	**Losartan n(%)**	**Placebo N(%)**
**Not related**	**Mild**	1 (33.33%)	0 (0%)
	**Moderate**	2 (66.67%)	0 (0%)
	**Severe**	0 (0%)	1 (100%)
	**Missing**	0 (0%)	0 (0%)
	**Total**	**3**	**1**
**Possibly related**	**Mild**	0 (0%)	0 (0%)
	**Moderate**	0 (0%)	0 (0%)
	**Severe**	0 (0%)	0 (0%)
	**Missing**	0 (0%)	0 (0%)
	**Total**	**0**	**0**
**Probably related**	**Mild**	0 (0%)	0 (0%)
	**Moderate**	0 (0%)	0 (0%)
	**Severe**	0 (0%)	0 (0%)
	**Missing**	0 (0%)	0 (0%)
	**Total**	**0**	**0**
**Missing**	**Mild**	0 (0%)	0 (0%)
	**Moderate**	0 (0%)	0 (0%)
	**Severe**	0 (0%)	0 (0%)
	**Missing**	0 (0%)	0 (0%)
	**Total**	**0**	**0**
**Arm Totals**		**3**	**1**

Table 14 (b): Line listing of SAEs on ITT population

**Cat**	**Placebo**	**Losartan**
fall and rib fracture	1	0
dislocation of left 1st metacarpal	0	1
epistaxis	0	1
fracture of left 1st metacarpal	0	1
**Total**	**1**	**3**

7. OUTCOME DATA

7.1 Definition and Calculation of Outcome Measures

Loss to follow-up is when contact is lost with some patients and therefore the planned data collection for that patient cannot be completed. The study population is the ITT population.

Primary Outcome Measure

Kleiner fibrosis stage from two independent blinded histopathologists (stored in MACRO as a consensus) from liver biopsies (Kleiner DE et al Hepatology 2005) from pre-treatment (within 6 months of visit 2 - baseline) to end of study (visit 8 – 96 weeks+/- 7days).

Table 15: Kleiner fibrosis stage & description. Note in analysis 1a) b) and c) are counted as 1 as the letter is related to location.

Score	Histological description
0	No fibrosis
1a	Zone 3 sinusoidal, seen on trichome
1b	Zone 3 sinusoidal, seen on H&E
1c	Portal/periportal only
2	Zone 3 and periportal fibrosis
3	Bridging fibrosis
4	Cirrhosis

Table 16: Concordance between observers. Note once observer has one missing score.

Observer 1 \ Observer 2	0	1	2	3	4	Total
**0**	4	1	0	0	0	5 (7.94%)
**1**	0	6	2	0	0	8 (12.70%)
**2**	0	0	25	2	0	27 (42.86%)
**3**	0	0	0	18	0	18 (28.57%)
**4**	0	0	0	0	5	5 (7.94%)
**Total**	4 (6.35%)	7 (11.11%)	27 (42.86%)	20 (31.75%)	5 (7.94%)	63

The consensus of the two Kleiner fibrosis stages was checked using Table 16. Any mismatches were dealt with and an agreement made for the final analysis set.

The median change in Kleiner fibrosis stages was calculated between the placebo and intervention groups.

Table 17: Number of patients in ITT and KFL analysis set.

Population	Visit	Time from randomisation	N (%)
**ITT**	**Baseline**	0, (0-0), (-5,9)	45
	**96 weeks**	679, (668-699), (193-792)	41 (91.11%)
**KFS analysis set**	**Baseline**	Median (IQR, Range)	
	**96 weeks**	Median (IQR, Range)	

Secondary Outcome Measures

1. Radiological (fibroscan) markers of fibrosis (screening visit 1, visit 6 at 48 weeks and end of study visit 8 at 96 weeks). Fibroscan measures the stiffness of the liver. Several measurements are taken so the median should be used.
2. Serological (ELF and Fibromax) markers of fibrosis (screening visit 1, visit 6 at 48 weeks and end of study visit 8 at 96 weeks). Fibromax markers include alpha-2 macroglobin, haptoglobin, apolipoprotein A1, total bilirubin, ALT, AST, GGT, fasting glucose, triglycerides, total cholesterol.
3. Change in NAFLD activity score (NAS) from baseline (determined from biopsies at trial entry (within 6 months of visit 2 - baseline) and end of study visit 8 at 96 weeks). The NAFLD activity score is based on features of active injury that are potentially reversible and is defined as the unweighted sum of scores for steatosis (0-3), lobular inflammation (0-3) and hepatocyte ballooning (0-2). Therefore it is a number from 0-8.

(Table describing NAFLD activity score (NAS) is given at this webpage http://tpis.upmc.com/changebody.cfm?url=/tpis/schema/NAFLD2006.jsp).

4. Response is defined as end-of-treatment (visit 8 at 96 weeks) Kleiner liver fibrosis stage less than baseline Kleiner liver fibrosis stage (within 6 months of visit 2 – baseline).

5. Change in disease activity score according to SAF. SAF (Steatosis, activity, fibrosis) score is made up of three scores: steatosis (0-3), disease activity (0-4) (an unweighted sum of hepatocyte ballooning (0-2) and lobular inflammation (0-2)) and stage of fibrosis (0-4). SAF is not an additive score and is reported $S_xA_yF_z$ where x,y and z are the relevant scores. This is taken from the biopsies at trial entry and 96 weeks.

6. Other histological data includes steatosis, ballooning, lobular and portal inflammation, Mallory-Denk bodies, the Newcastle 7-tier staging system for fibrosis, and presence of apoptotic cells. Type and location of steatosis, NASH global grade (according to Brunt 1999), megamitochondria, glycogenated nuclei, microgranulomas, lipogranulomas, and ductular reaction may be available.

7. M30 (fragmented CK18 serum data) may be available.

8. Quality of life is measured using CLDQ and SF-36 questionnaires at screening visit 1, visit 6 at 48 weeks and end of study visit 8 at 96 weeks.

   SF-36 is a health survey with 26 questions and 8 domains (vitality, physical functioning, bodily pain, general health perceptions, physical role functioning, emotional role functioning, social role functioning, mental health). Each domain is a weighted sum of its questions and is transformed on to a 0-100 scale. Lower scores represent more disability. We will use the proprietary scoring algorithms developed by Ware et al for the SF-36 .

   CLDQ is the Chronic Liver disease questionnaire which has 29 items in the following domains abdominal symptoms, fatigue, systemic systems, activity, emotional function and worry on a 1-7 point scale ranging from “all of the time” to “none of the time”. To get the domain score we divide the item scores by the number of item scores in that domain so that the total for the domain is on the 7 point scale as recommended in Younossi et al 1999 (Gut).

7.2 Patient follow-up

Table 18: Summary of patient follow up by randomised treatment arm. Note 3 patients completed visit 8 having missed previous two.

	Randomised treatment arm	Randomised n	Visit Number 3 (1w+/-2d)	4 (4w+/-7d)	5 (24w+/-7d)	6 (48w +/-7d)	7 (72w+/-7d)	8 (96w+/-7d) End of treatment	9 (108w +/-7d)
Number continuing study visits (% of randomised)	Lorsartan	24	24 (100%)	23 (95.83%)	23 (95.83%)	20 (83.33%)	19 (79.17%)	20 (83.33%)	16 (66.67%)
	Placebo	21	21 (100%)	21 (100%)	21 (100%)	19 (90.48%)	19 (90.48%)	21 (100%)	18 (85.71%)
Number continuing study visits on study drug (% of randomised)	Lorsartan	24	24 (100%)	23 (95.83%)	23 (95.83%)	20 (83.33%)	19 (79.17%)	20 (83.33%)	16 (66.67%)
	Placebo	21	21 (100%)	21 (100%)	20 (95.24%)	18 (85.71%)	18 (85.71%)	20 (95.24%)	17 (80.95%)

Table 19: The time in trial (from randomization to date of last follow up) by treatment arm.

	Lorsartan	Placebo
Time in trial (days)		
Median	764.5	764
IQR	(761-774)	(756-784)
Range	(750-914)	(728-888)
Number of patients	n=16	n=18

7.3 Analyses of outcome data

Analysis of primary outcome measure

This analysis was completed with the ITT study population. Although the main aim of the study is no longer to establish clinical effectiveness of Losartan a formal comparison of the two groups was still undertaken based on the primary outcome measure.

An interval estimate of clinical effectiveness was produced and examination of the upper and lower limits gave an indication of the reduced power to detect a clinical significant difference.

The primary analysis for efficacy was based on change in Kleiner fibrosis score from baseline to end of treatment (0-24 months) defined as 24 month score minus baseline score.

Given the small number of cases no data imputation is planned.

No significance testing is performed.

Table 20: Summary statistics for the primary outcome measure by randomised treatment arm and by visit.

Variable	Losartan		Placebo	
	**Baseline (Visit 2) n=15 pre biopsy**	**96 weeks (Visit 8) n=15 post biopsy**	**Baseline (Visit 2) n=17 Pre biopsy**	**96 weeks (Visit 8) n=17 post biopsy**
Kleiner fibrosis stage	0: 1 (6.67%)<br>1: 3 (20%)<br>2: 6 (40%)<br>3: 4 (26.67%)<br>4: 1 (6.67%)	0: 1 (6.67%)<br>1: 2 (13.33%)<br>2: 7 (46.67%)<br>3: 4 (26.67%)<br>4: 1 (6.67%)	0: 1 (5.88%)<br>1: 0 (0%)<br>2: 8 (47.06%)<br>3: 7 (41.18%)<br>4: 1 (5.88%)	0: 1 (5.88%)<br>1: 2 (11.76%)<br>2: 6 (35.29%)<br>3: 6 (35.29%)<br>4: 2 (11.76%)
Mean (sd)	2.07 (1.03)	2.13 (0.99)	2.41 (0.87)	2.35 (1.06)
Median (IQR,range)	2<br>(1-3,0-4)	2<br>(2-3,0-4)	2<br>(2-3,0-4)	2<br>(2-3,0-4)

Table 21: Comparison of the number of responders. * No significance testing was performed.

	**Lorsartan**	**Placebo**
**Responder**	1 (6.67%)	4 (23.53%)
**Non-responder**	14 (93.33%)	13 (76.47%)
**n**	15	17

	**Point estimate**	**95% Confidence Interval (exact)**
**Odds Ratio**	0.2321429	(0.00, 2.88)

Table 22: Estimates for the median at baseline and the median change from baseline to 96 weeks by randomised treatment arm. * No significance testing was performed.

Variable	Losartan n=15	P value*	Placebo n=17	P value*
Change in Kleiner fibrosis stage from baseline to 96 weeks (Visit 8)	-1: 1 (6.67%)<br>0: 12 (80%)<br>1: 2 (13.33%)		-1 4 (23.53%)<br>0 10 (58.82%)<br>1 3 (17.65%)	
Median (IQR,range)	0<br>(0-0),(-1,1)		0<br>(0-0),(-1,+1)	

n* = number of patients with both baseline and 96 week measure. Shaded p values as this is not powered.

Analysis of secondary outcome measures

This analysis was performed using the ITT study population. Appropriate descriptive statistics are reported below for the following outcome measures by treatment arm and by visit (dependent on the amount of data available).

- radiological (fibroscan) markers of fibrosis
- serological (ELF and Fibroscan) markers of fibrosis
- NAFLD activity score
- CLDQ
- SF-36
- Biomarkers - ELF, AST/ALT.
- Histological measures

SF-36 standard scoring

Variable	Visit 2 n=45		Visit 6 n=39		Visit 8 n=41	
	**Losartan n=24**	**Placebo n=21**	**Losartan n=20**	**Placebo n=19**	**Losartan n=20**	**Placebo n=21**
**Physical functioning (PF)** Median (range), (IQR)	87.5 (0-100) (72.5-95)	85 (0-100) (50-95)	78.40 (0-100) (72.5-95)	n=18 72.5 (0-100) (40-95)	n=18 82.50 (20-100) (65.01-90)	n=20 82.50 (15-100) (57.5-95)
**Physical role functioning (RP)** Median (range), (IQR)	n=23 100 (0-100) (56.25-100)	87.5 (0-100) (56.25-100)	87.5 (25-100) (50-100)	n=18 81.25 (6.25-100) (43.75-100)	n=18 87.5 (0-100) (56.25-100)	n=20 71.88 (31.25-100) (50-100)
**Body Pain (BP)** Median (range), (IQR)	84 (12-100) (62-92)	74 (12-100) (41-100)	n=19 62 (0-100) (41-84)	n=18 72 (22-100) (41-84)	n=18 62 (12-100) (41-100)	n=20 52 (22-100) (51-84)
**General Health perception (GH)** Median (range), (IQR)	59.5 (5-97) (47-78.5)	42 (15-97) (25-67)	53.5 (0-97) (37.5-74.5)	n=18 52 (30-77) (35-72)	n=18 56 (0-92) (40-72)	n=20 43.5 (20-92) (30-57)
**Vitality (VT)** Median (range), (IQR)	62.5 (0-93.75) (50-78.13)	n=20 43.75 (0-93.75) (15.63-68.75)	50 (0-87.5) (37.5-68.75)	n=18 56.25 (6.25-81.25) (31.25-62.5)	n=18 53.13 (0-87.5) (37.5-75)	n=20 50 (0-87.5) (28.13-75)
**Social role functioning (SF)** Median (range), (IQR)	87.5 (0-100) (25-100)	100 (25-100) (50-100)	81.25 (0-100) (50-100)	n=18 75 (25-100) (50-100)	n=18 100 (0-100) (62.5-100)	n=20 75 (25-100) (56.25-87.5)

**Emotional role functioning (RE)**<br>Median (range), (IQR)	n=23<br>100 (0-100)<br>(50-100)	83.33 (0-100)<br>(50-100)	n=19<br>91.67 (8.33-100)<br>(50-100)	n=18<br>75 (25-100)<br>(50-91.67)	n=18<br>91.67 (0-100)<br>(75-100)	n=20<br>75 (16.67-100)<br>(54.17-95.84)
**Mental Health (MH)**<br>Median (range), (IQR)	77.5 (25-100)<br>(57.5-90)	n=20<br>70 (30-100)<br>(45-85)	62.5 (0-90)<br>(52.5-75)	n=18<br>70 (35-100)<br>(55-80)	n=18<br>72.5 (5-95)<br>(55-90)	n=20<br>75 (5-100)<br>(46.88-90)
**Physical Component Summary (PCS)**<br>Median (range), (IQR)	n=23<br>53.55 (24.85-59.13)<br>(46.41-57.39)	n=20<br>49.06 (25.27-59.37)<br>(40.73-55.23)	n=19<br>51.38 (24.6-65.8)<br>(43.19-55.74)	n=18<br>44.29 (30.4-59.34)<br>(38.52-54.19)	n=18<br>49.36 (34.31-61.08)<br>(42.78-54.21)	n=20<br>45.73 (27.76-60.02)<br>(42.31-53.64)
**Mental Component Summary (MCS)**<br>Median (range), (IQR)	n=23<br>53.25 (15.52-60.61)<br>(40.41-58.64)	n=20<br>48.09 (24.27-66.84)<br>(34.46-56.64)	n=19<br>45.92 (6.06-59.2)<br>(35.86-54.85)	n=18<br>49.64 (23.64-58.34)<br>(39.86-52.36)	n=18<br>51.92 (13.74-61.56)<br>(41.41-59.92)	n=20<br>49.23 (16.94-65.29)<br>(38.15-54.41)

SF-36 T scoring based on US population (mean 50, sd 10). Summary measures calculated using oblique method.

Variable	Visit 2 n=45		Visit 6 n=39		Visit 8 n=41	
	**Losartan n=24**	**Placebo n=21**	**Losartan n=20**	**Placebo n=19**	**Losartan n=20**	**Placebo n=21**
**Physical functioning (PF_NBS)** Median (range), (IQR)	52.76 (19.26-57.54) (47.02-55.63)	51.8 (19.26-57.54) (38.4-55.63)	49.27 (19.26-57.54) (47.02-55.63)	n=18 47.02 (19.26-57.54) (34.57-55.63)	n=18 50.84 (26.92-57.54) (44.15-53.71)	n=20 50.84 (25.01-57.54) (41.28-55.63)
**Physical role functioning (RP_NBS)** Median (range), (IQR)	n=23 57.16 (21.23-57.16) (41.44-57.16)	52.66 (21.23-57.16) (41.44-57.16)	52.67 (30.21-57.16) (39.20-57.16)	n=18 50.42 (23.47-57.16) (36.95-57.16)	n=18 52.67 (21.23-57.16) (41.44-57.16)	n=20 47.05 (32.46-57.16) (39.19-54.91)
**Body Pain (BP_NBS)** Median (range), (IQR)	55.55 (26.52-62) (46.68-58.78)	n=21 51.51 (26.52-62) (38.21-62)	n=19 46.68 (21.68-62) (38.21-55.55)	n=18 50.71 (30.55-62) (38.21-55.55)	n=18 46.68 (26.52-62) (38.21-62)	n=20 42.64 (30.55-62) (42.24-55.55)
**General Health perception (GH_NBS)** Median (range), (IQR)	47.24 (21.33-65.07) (41.3-56.28)	38.92 (26.08-65.07) (30.84-50.81)	44.39 (18.95-65.07) (36.78-54.38)	n=18 43.68 (33.22-55.56) (35.59-53.19)	n=18 45.58 (18.95-62.7) (37.97-53.19)	n=20 39.64 (28.46-62.7) (33.22-46.05)
**Vitality (VT_NBS)** Median (range), (IQR)	52.6 (22.89-67.45) (46.66-60.03)	n=20 43.69 (22.89-67.45) (30.32-55.57)	46.66 (22.89-64.48) (40.72-55.57)	n=18 49.63 (25.86-61.51) (37.74-52.6)	n=18 48.15 (22.89-64.48) (40.72-58.54)	n=20 46.66 (22.89-64.48) (36.26-58.54)
**Social role functioning (SF_NBS)** Median (range),	52.33 (17.23-57.34)	57.34 (27.26-57.34)	49.82 (17.23-57.34)	n=18 47.31 (27.26-57.34)	n=18 57.34 (17.23-57.34)	n=20 47.31 (27.26-57.34)

(IQR)	(39.80-57.34)	(37.29-57.34)	(37.29-57.34)	(37.29-57.34)	(42.3-57.34)	(39.80-52.33)
**Emotional role functioning (RE_NBS)**<br>Median (range), (IQR)	n=23<br>56.17 (14.39-56.17)<br>(35.28-56.17)	49.2 (14.39-56.17)<br>(35.28-56.17)	n=19<br>52.69 (17.87-56.17)<br>(35.28-56.17)	n=18<br>45.72 (24.83-56.17)<br>(35.28-52.69)	n=18<br>52.69 (14.39-56.17)<br>(45.72-56.17)	n=20<br>45.72 (21.35-56.17)<br>(37.02-54.43)
**Mental Health (MH_NBS)**<br>Median (range), (IQR)	52.18 (24.71-63.95)<br>(41.71-58.72)	n=20<br>48.26 (27.32-63.95)<br>(35.18-56.1)	44.33 (11.63-58.72)<br>(39.10-50.87)	n=18<br>48.25 (29.94-63.95)<br>(40.4-53.48)	n=18<br>49.56 (14.24-61.33)<br>(40.4-58.72)	n=20<br>50.87 (14.24-63.95)<br>(36.16-58.72)
**Physical Component Summary (PCS_OBL)**<br>Median (range), (IQR)	n=23<br>53.7 (18.02-61.35)<br>(44.35-57.69)	n=20<br>48.55 (19.15-60.31)<br>(37.46-56)	n=19<br>51.51 (21.61-62.82)<br>(37.1-56.72)	n=18<br>42.09 (30.54-59.89)<br>(37.91-54.01)	n=18<br>47.53 (24.95-60.55)<br>(44.87-56.15)	n=20<br>46.89 (26.29-58.53)<br>(38.31-53.03)
**Mental Component Summary (MCS_OBL)**<br>Median (range), (IQR)	n=23<br>54.92 (15.87-62.33)<br>(41.55-58.76)	n=20<br>47.75 (18.54-62.27)<br>(33.9-57.62)	n=19<br>45.97 (6.89-60.4)<br>(33.85-54.85)	n=18<br>46.49 (24-59.62)<br>(37.61-52.95)	n=18<br>52.82 (10.84-62.21)<br>(41.78-58.91)	n=20<br>47.59 (15.64-63.09)<br>(35.01-53.03)

CLDQ results - domain scores are calculated ignoring any missing data

	Visit 2		Visit 6		Visit 8	
**Domain**	**Losartan n=24**	**Placebo n=21**	**Losartan n=20**	**Placebo n=19**	**Losartan n=20**	**Placebo n=21**
**AS**<br>Median (range)<br>(IQR)	6.50 (2.33,7.00)<br>(5.83,7.00)	6.33 (1.67,7.00)<br>(5.00,7.00)	6.33 (2.33,7.00)<br>(5.00,7.00)<br>n=20	6.17 (3.67,7.00)<br>(4.33,7.00)<br>n=18	6.50 (2.33,7.00)<br>(5.33,7.00)<br>n=18	6.17 (2.67,7.00)<br>(4.50,7.00)<br>n=20
**FA**<br>Median (range)<br>(IQR)	6.20 (1.00,7.00)<br>(4.50,6.50)	5.40 (1.00,7.00)<br>(2.80,6.20)	4.80 (1.80,7.00)<br>(3.55,6.20)<br>n=20	5.30, (2.80,6.80)<br>(3.80,6.00)<br>n=18	5.10 (1.40,7.00)<br>(4.40,5.80)<br>n=18	5.10 (1.20,6.80)<br>(3.20,5.70)<br>n=20
**SS**<br>Median (range)<br>(IQR)	6.10 (2.80,7.00)<br>(5.10,6.60)	5.80 (2.40,7.00)<br>(4.20,6.60)	5.10 (2.60,7.00)<br>(4.50,6.30)<br>n=20	5.10 (3.20,7.00)<br>(4.50,6.00)<br>n=18	5.30 (4.00,7.00)<br>(5.00,6.00)<br>n=18	5.30 (2.60,6.80)<br>(4.00,6.00)<br>n=20
**AC**<br>Median (range)<br>(IQR)	7.00 (2.00,7.00)<br>(5.67,7.00)	6.00 (2.33,7.00)<br>(5.00,6.67)	6.25 (2.67,7.00)<br>(5.00,7.00)<br>n=20	5.33 (3.67,7.00)<br>(5.00,6.33)<br>n=18	6.33 (3.00,7.00)<br>(5.50,7.00)<br>n=18	5.83 (2.33,7.00)<br>(4.33,6.50)<br>n=20
**EF**<br>Median (range)<br>(IQR)	5.88 (2.13,7.00)<br>(4.56,6.69)	5.50 (2.25,7.00)<br>(3.63,6.25)	5.50 (1.25,7.00)<br>(3.88,6.50)<br>n=20	4.94 (3.13,7.00)<br>(4.38,6.13)<br>n=18	5.38 (1.25,7.00)<br>(4.38,6.63)<br>n=18	5.13 (2.13,7.00)<br>(4.13,6.13)<br>n=20
**WO**<br>Median (range)<br>(IQR)	6.20 (2.80,7.00)<br>(5.00,7.00)	6.00 (2.20,7.00)<br>(4.00,6.60)	6.43 (2.20,7.00)<br>(4.60,7.00)<br>n=20	6.10 (3.60,7.00)<br>(4.80,7.00)<br>n=18	6.70 (1.80,7.00)<br>(3.80,7.00)<br>n=18	5.60 (2.60,7.00)<br>(4.30,6.70)<br>n=20
**Overall**<br>Median (range)	6.21 (2.38,7.00)	5.83 (2.38,6.89)	5.79 (3.07,7.00)	5.38 (3.83,6.90)	5.45 (2.31,6.79)	5.58 (3.00,6.90)

(IQR)	(4.83,6.52)	(4.17,6.31)	(4.40,6.34)<br>n=20	(4.31,6.21)<br>n=18	(4.69,6.41)<br>n=18	(4.14,6.16)<br>n=20
**Change AS**<br>Median (range)<br>(IQR)					0.00 (-3.67,2.00)<br>(-1.33,0.00)<br>n=18	0.00 (-2.33,3.67)<br>(-0.33,0.33)<br>n=20
**Change FA**<br>Median (range)<br>(IQR)					-0.70 (-2.00,0.40)<br>(-1.00,0.00)<br>n=18	-0.20 (-1.60,0.80)<br>(-0.80,0.40)<br>n=20
**Change SS**<br>Median (range)<br>(IQR)					-0.50 (-2.00,0.40)<br>(-1.00,-0.20)<br>n=18	-0.30 (-1.40,1.40)<br>(-0.60,0.00)<br>n=20
**Change AC**<br>Median (range)<br>(IQR)					-0.50 (-3.33,2.33)<br>(-1.33,0.00)<br>n=18	-0.33 (-3.00,2.00)<br>(-0.67,0.17)<br>n=20
**Change EF**<br>Median (range)<br>(IQR)					0.00 (-3.63,1.13)<br>(-0.75,0.25)<br>n=18	-0.13 (-1.75,1.00)<br>(-0.69,0.46)<br>n=20
**Change WO**<br>Median (range)<br>(IQR)					0.00 (-3.60,1.20)<br>(-0.20,0.60)<br>n=18	0.10 (-2.20,1.20)<br>(-0.50,0.80)<br>n=20
**Change Overall**<br>Median (range)<br>(IQR)					-0.19 (-2.76,0.38)<br>(-0.48,0.07)<br>n=18	-0.08 (-1.07,0.79)<br>(-0.48,0.12)<br>n=20

CLDQ results – To deal with missing data we have set the domain score to missing if >1 element in domain is missing

	Visit 2		Visit 6		Visit 8	
**Domain**	**Losartan n=24**	**Placebo n=21**	**Losartan n=20**	**Placebo n=19**	**Losartan n=20**	**Placebo n=21**
**AS** Median (range) (IQR)	6.50 (2.33,7.00) (5.83,7.00) n=24	6.33 (1.67,7.00) (5.00,7.00) n=20	6.33 (2.33,7.00) (4.67,7.00) n=19	6.17 (3.67,7.00) (4.33,7.00) n=18	6.00 (2.33,7.00) (5.33,7.00) n=17	6.17 (2.67,7.00) (4.50,7.00) n=20
**FA** Median (range) (IQR)	6.20 (1.00,7.00) (4.20,6.40) n=23	5.40 (1.00,7.00) (2.80,6.20) n=21	5.20 (1.80,7.00) (3.60,6.20) n=18	5.30 (2.80,6.80) (3.80,6.00) n=18	5.00 (1.40,7.00) (4.40,5.80) n=17	5.10 (1.20,6.80) (3.20,5.70) n=20
**SS** Median (range) (IQR)	6.00 (2.80,7.00) (5.00,6.60) n=23	5.80 (2.40,7.00) (4.20,6.60) n=21	5.10 (2.60, 7.00) (4.50, 6.30) n=20	5.20 (3.20, 7.00) (4.20, 6.00) n=15	5.20 (4.00,7.00) (5.00,6.00) n=17	5.30 (2.60,6.80) (4.00,6.00) n=20
**AC** Median (range) (IQR)	7.00 (2.00,7.00) (5.67,7.00) n=24	6.00 (2.33,7.00) (5.00,6.67) n=21	6.00 (2.67,7.00) (5.00,7.00) n=19	5.33 (3.67,7.00) (5.00,6.33) n=18	6.33 (3.00,7.00) (5.67,7.00) n=17	5.83 (2.33,7.00) (4.33,6.50) n=20
**EF** Median (range) (IQR)	5.88 (2.13,7.00) (4.56,6.69) n=24	5.13 (2.25,7.00) (3.56,6.25) n=20	5.63 (1.25,7.00) (3.63,6.50) n=19	4.94 (3.13, 7.00) (4.25, 6.06) n=16	5.25 (1.25,7.00) (4.38,6.50) n=17	5.25 (2.13,7.00) (3.75,6.13) n=19
**WO** Median (range) (IQR)	6.20 (2.80,7.00) (5.00,7.00) n=24	6.00 (2.20,7.00) (4.00,6.60) n=21	6.60 (2.20,7.00) (4.60,7.00) n=19	6.20 (3.60,7.00) (4.80,7.00) n=15	6.80 (1.80,7.00) (3.80,7.00) n=17	5.60 (2.60,7.00) (4.30,6.70) n=20
**Overall** Median (range) (IQR)	6.17 (2.38,6.86) (4.79,6.48) n=23	5.62 (2.38,6.59) (3.69,6.31) n=19	6.00 (3.07,7.00) (4.31,6.38) n=18	5.22 (3.83,6.90) (4.31,5.76) n=14	5.33 (2.31,6.76) (4.60,6.36) n=16	5.59 (3.00,6.90) (4.07,6.17) n=19
**Change AS** Median (range) (IQR)					0.00 (-3.67,2.00) (-1.33,0.00) n=17	0.00 (-2.33,3.67) (-0.33,0.33) n=19

**Change FA**<br>Median (range)<br>(IQR)					-0.60 (-1.80,0.40)<br>(-0.90,0.00)<br>n=16	-0.20 (-1.60,0.80)<br>(-0.80,0.40)<br>n=20
**Change SS**<br>Median (range)<br>(IQR)					-0.50 (-1.60,0.40)<br>(-0.90,-0.10)<br>n=16	-0.30 (-1.40,1.40)<br>(-0.60,0.00)<br>n=20
**Change AC**<br>Median (range)<br>(IQR)					-0.33 (-3.33,2.33)<br>(-1.33,0.00)<br>n=17	-0.33 (-3.00,2.00)<br>(-0.67,0.17)<br>n=20
**Change EF**<br>Median (range)<br>(IQR)					0.00 (-3.63,1.13)<br>(-0.75,0.25)<br>n=17	-0.13 (-1.75,1.00)<br>(-0.75,0.50)<br>n=18
**Change WO**<br>Median (range)<br>(IQR)					0.00 (-3.60,1.20)<br>(-0.20,0.60)<br>n=17	0.10 (-2.20,1.20)<br>(-0.50,0.80)<br>n=20
**Change Overall**<br>Median (range)<br>(IQR)					-0.17 (-2.28,0.38)<br>(-0.48,0.07)<br>n=15	-0.10 (-1.07,0.79)<br>(-0.59,0.14)<br>n=17

To deal with missing data we have set domain score to missing if >50% of items are missing

	Visit 2		Visit 6		Visit 8	
**Domain**	**Losartan n=24**	**Placebo n=21**	**Losartan n=20**	**Placebo n=19**	**Losartan n=20**	**Placebo n=21**
**AS** Median (range) (IQR)	6.50 (2.33,7.00) (5.83,7.00) n=24	6.33 (1.67,7.00) (5.00,7.00) n=21	6.33 (2.33,7.00) (5.00,7.00) n=20	6.17 (3.67,7.00) (4.33,7.00) n=18	6.50 (2.33,7.00) (5.33,7.00) n=18	6.17 (2.67,7.00) (4.50,7.00) n=20
**FA** Median (range) (IQR)	6.20 (1.00,7.00) (4.50,6.50) n=24	5.40 (1.00,7.00) (2.80,6.20) n=21	4.80 (1.80,7.00) (3.55,6.20) n=20	5.30 (2.80,6.80) (3.80,6.00) n=18	5.10 (1.40,7.00) (4.40,5.80) n=18	5.10 (1.20,6.80) (3.20,5.70) n=20
**SS** Median (range) (IQR)	6.10 (2.80,7.00) (5.10,6.60) n=24	5.80 (2.40,7.00) (4.20,6.60) n=21	5.10 (2.60,7.00) (4.50,6.30) n=20	5.10 (3.20,7.00) (4.48,6.00) n=16	5.30 (4.00,7.00) (5.00,6.00) n=18	5.30 (2.60,6.80) (4.00,6.00) n=20
**AC** Median (range) (IQR)	7.00 (2.00,7.00) (5.67,7.00) n=24	6.00 (2.33,7.00) (5.00,6.67) n=21	6.25 (2.67,7.00) (5.00,7.00) n=20	5.33 (3.67,7.00) (5.00,6.33) n=18	6.33 (3.00,7.00) (5.50,7.00) n=18	5.83 (2.33,7.00) (4.33,6.50) n=20
**EF** Median (range) (IQR)	5.88 (2.13,7.00) (4.56,6.69) n=24	5.50 (2.25,7.00) (3.63,6.25) n=21	5.63 (1.25,7.00) (3.63,6.50) n=19	4.94 (3.13,7.00) (4.38,6.13) n=18	5.25 (1.25,7.00) (4.38,6.50) n=17	5.13 (2.13,7.00) (4.13,6.13) n=20
**WO** Median (range) (IQR)	6.20 (2.80,7.00) (5.00,7.00) n=24	6.00 (2.20,7.00) (4.00,6.60) n=21	6.43 (2.20,7.00) (4.60,7.00) n=20	6.10 (3.60,7.00) (5.20,6.80) n=16	6.70 (1.80,7.00) (3.80,7.00) n=18	5.60 (2.60,7.00) (4.30,6.70) n=20
**Overall** Median (range) (IQR)	6.21 (2.38,7.00) (4.83,6.52) n=24	5.83 (2.38,6.89) (4.17,6.31) n=21	5.79 (3.07,7.00) (4.40,6.34) n=20	5.38 (3.83,6.90) (4.31,6.21) n=18	5.45 (2.31,6.79) (4.69,6.41) n=18	5.58 (3.00,6.90) (4.14,6.16) n=20
**Change AS** Median (range) (IQR)					0.00 (-3.67,2.00) (-1.33,0.00) n=18	0.00 (-2.33,3.67) (-0.33,0.33) n=20
**Change FA** Median (range)					-0.70 (-2.00,0.40) (-1.00,0.00)	-0.20 (-1.60,0.80) (-0.80,0.40)

(IQR)					n=18	n=20
**Change SS**<br>Median (range)<br>(IQR)					-0.50 (-2.00,0.40)<br>(-1.00,-0.20)<br>n=18	-0.30 (-1.40,1.40)<br>(-0.60,0.00)<br>n=20
**Change AC**<br>Median (range)<br>(IQR)					-0.50 (-3.33,2.33)<br>(-1.33,0.00)<br>n=18	-0.33 (-3.00,2.00)<br>(-0.67,0.17)<br>n=20
**Change EF**<br>Median (range)<br>(IQR)					0.00 (-3.63,1.13)<br>(-0.75,0.25)<br>n=17	-0.13 (-1.75,1.00)<br>(-0.69,0.46)<br>n=20
**Change WO**<br>Median (range)<br>(IQR)					0.00 (-3.60,1.20)<br>(-0.20,0.60)<br>n=18	0.10 (-2.20,1.20)<br>(-0.50,0.80)<br>n=20
**Change Overall**<br>Median (range)<br>(IQR)					-0.19 (-2.76,0.38)<br>(-0.48,0.07)<br>n=18	-0.08 (-1.07,0.79)<br>(-0.48,0.12)<br>n=20

- Change is change from Baseline i.e. visit 8 score minus BL score)
- 6 domains: AS=Abdominal Symptoms; FA= Fatigue; SS=Systemic symptoms; AC=Activity; EF=Emotional function; WO=Worry
- 1 (symptoms all the time) is worse than 7 (symptom free).

Table 23: Summary statistics for study outcome measures by randomised treatment arm and by visit.

Variable	Screening pre-baseline (Visit 1) Median (range)		48 weeks (Visit 6) Median (range)		96 weeks (Visit 8) Median (range)	
	**Losartan**	**Placebo**	**Losartan**	**Placebo**	**Losartan**	**Placebo**
**n**	**24**	**21**	**20**	**19**	**20**	**21**
**Fibroscan - Liver stiffness**	8.15 (5.2-17.3) n=10	6.05 (3-11.9) n=6	4.95 (0.3-16.6) n=8	11.9 (3.9-15) n=3	5.05 (3.4-15.4) n=8	5.95 (3.6-20.6) n=4
**Fibroscan - Stiffness median**	8.9 (1.6-26.6) n=6	7.95 (7.1-8.8) n=2	7.95 (5.8-10.1) n=2	No observations	9.5 (0-14) n=4	No observations
**ELF**	8.84 (6.54-11.83) n=23	7.96 (6.43-10.28) n=19	9.15 (7.36-11.88) n=19	8.47 (6.5-10.59) n=18	9.45 (7.91-11.03) n=18	8.97 (7.31-10.8) n=16

Biochemistry results

Variable	Baseline (Visit 2) Median (range)		1 week +/- 2 days (Visit 3) Median (range)		4 weeks (visit 4) Median (range)		24 weeks (visit 5) Median (range)		48 weeks (Visit 6) Median (range)		72 weeks (visit 7) Median (range)		96 weeks (Visit 8) Median (range)		108 weeks (visit 9) Median (range)	
	**Losartan**	**Placebo**	**Losartan**	**Placebo**	**Losartan**	**Placebo**	**Losartan**	**Placebo**	**Losartan**	**Placebo**	**Losartan**	**Placebo**	**Losartan**	**Placebo**	**Losartan**	**Placebo**
**N**	**24**	**21**	**24**	**21**	**23**	**21**	**23**	**21**	**20**	**19**	**19**	**19**	**20**	**21**	**16**	**18**
Time from randomisation (days)	0 (-5,3)	0 (0,9)	7 (4-30)	7 (6-21)	29 (27-59)	29 (20-46)	168 (153-213)	170 (140-198)	339.5 (328-423)	336 (308-371)	510 (503-539)	510 (476-610)	679 (224-756)	681 (193-792)	764.5 (750-914)	764 (728-888)
Sodium median (range)	139.5 (135-143)	141 (136-145)	139.5 (136-145)	141 (135-146)	140 (134-146)	141 (135-144)	140 (135-144)	140 (133-142)	140.5 (136-144)	139.5 (136-142) n=18	139 (134-144)	139 (132-143) n=18	139 (137-144)	140 (135-143) n=19	139.5 (137-144) n=14	139.5 (132-142)
Potassium median (range)	4.35 (3.8-4.8)	4.3 (3.7-4.8)	4.3 (3.8-5) n=23	4.3 (3.5-5.3)	4.3 (3.7-4.8)	4.2 (3.6-5)	4.2 (3.6-4.7)	4.2 (3.5-4.8)	4.15 (3.5-4.8)	4.2 (3.4-5) n=18	4.3 (3.5-4.8)	4.15 (3.5-5.2) n=18	4.3 (3.7-4.9) n=19	4.4 (3.5-4.6) n=19	4.1 (3.3-5) n=13	4.15 (3.2-5.2) n=16
Urea median (range)	4.9 (3.2-7.9) n=23	5 (3.3-6.9)	5.55 (2.9-7.3)	5 (3.6-6.7)	5.4 (3.2-7.6)	4.8 (2.8-7)	5.1 (2.9-7.6)	5 (3.2-6.3)	4.9 (3.6-7.8)	5.4 (3.4-6.7) n=18	5 (3.1-9.1)	4.75 (3.3-6.9) n=18	4.85 (3.1-7.1)	4.9 (2.3-7.3) n=19	5.05 (2.9-9.1) n=14	5 (3.2-8.9)

Glucose median (range)	5.95 (4.4-17.1)	6.2 (3.6-15.9) n=20			6.3 (4.2-15.8) n=21	6.5 (2.5-11.5)	6.7 (4.3-17.1)	6.75 (4.5-18.5) n=20	7.2 (5-13.1) n=19	7.1 (4.1-17.8) n=18	9.3 (5.3-19.8) n=17	6.35 (4.4-20.7) n=18	7.65 (4.8-19.7)	6.7 (4.3-15.8) n=20	7.35 (5.6-12) n=12	7.2 (4.9-17.5) n=14
AST median (range)	35 (14-102) n=21	46 (30-70) n=18			37 (17-160) n=20	42 (3-124) n=19	29 (15-66) n=14	41 (23-133) n=16	35 (16-104) n=17	35 (20-85) n=17	32 (14-55) n=15	33.5 (20-181) n=16	33 (2-70) n=19	36 (17-110)	33 (17-47) n=13	39 (19-81) n=17
ALT median (range)	52.5 (21-136)	65 (33-135)			53 (16-179)	62 (33-147)	46 (16-85)	62 (28-138)	43 (17-101) n=19	46.5 (24-93) n=18	46 (18-73)	48 (22-151) n=18	34.5 (7-80)	52 (18-133) n=20	39 (17-59) n=13	55 (17-112) n=17
ALP median (range)	89.5 (44-173)	72 (49-116)			81 (40-184)	72 (47-121)	82 (36-174)	87 (48-106)	80.5 (38-164)	72 (49-164) n=18	90 (38-183)	81 (46-121) n=17	89.5 (37-191)	77 (46-106) n=19	97.5 (43-177) n=14	72 (43-124) n=17
Creatinine median (range)	75.5 (48-105)	72 (5-97)			76 (50-105)	74 (48-98)	73 (41-109)	72 (58-101)	70.5 (56-93)	67 (53-105) n=18	71 (46-100)	66.5 (53-92) n=18	73.5 (43-106)	68 (52-94) n=19	71 (44-98) n=14	66 (7-95) n=16
Bilirubin median (range)	10 (5-25)	10 (4-45)			10 (5-24)	10 (4-29)	9 (4-35)	11 (5-31)	10 (6-76)	9 (5-18) n=18	11 (5-29)	11 (6-28) n=18	9 (4-34)	11 (6-23) n=19	8 (5-15) n=13	10 (4-16) n=16
Albumin median (range)	44.5 (34-50)	46 (35-75)			44 (36-50)	46 (37-50)	45 (35-50)	44 (37-53)	43.5 (34-51)	46 (36-51) n=18	44 (36-51) n=18	45 (37-50) n=18	44.5 (34-50)	45 (36-51) n=19	45 (38-50) n=14	45 (37-51) n=15
Triglyceride median (range)	1.7 (0.9-7.9) n=23	2 (0.4-4.4) n=19			1.8 (0.8-6.2) n=21	1.6 (0.7-5.6) n=19	1.9 (0.6-6.2)	1.7 (0.5-4.9)	2.1 (0.7-5.3) n=19	1.65 (0.6-5.1) n=18	1.9 (0.8-12.4)	1.85 (0.7-3.6) n=18	2 (0.8-6.5) n=19	1.55 (0.4-4.4) n=20	2.7 (0.8-6.3) n=13	1.8 (1-4) n=17
HDL Choleste	1.1 (0.7-2.8)	1.1 (0.8-3.5)			1.15 (0.8-2)	1.1 (0.9-	1.2 (0.8-1.8)	1.1 (0.7-	1.1 (0.8-2)	1.1 (0.8-	1.2 (0.9-1.7)	1.1 (0.9-	1 (0.7-1.9)	1.1 (0.8-	1 (0.8-1.8)	1.1 (0.8-

rol median (range)	n=22	n=20			n=20	3.5) n=18	n=21	1.8) n=20	n=19	4.7) n=17		1.7) n=18	n=18	1.8) n=18	n=12	1.5) n=16
Total Choleste rol median (range)	4.3 (2.1-7.5) n=23	4.6 (1-6.5) n=20			4.6 (1.9-7.3) n=21	4.9 (2.4-5.9) n=19	4.1 (1.9-9)	4.8 (2.2-6.1)	4.3 (1-6.4) n=19	4.4 (1-6) n=18	4.3 (2-68)	4.35 (2.5-5.9) n=18	4.3 (2.2-7.6) n=18	4.4 (2.2-6.5) n=20	5.1 (2-67) n=13	4.3 (2.6-6.1) n=17
LDL Choleste rol	2.5 (0.8-3.8) n=19	3.2 (1.2-4.4) n=18			3 (1.2-31) n=17	2.9 (1-4) n=16	2.9 (0.7-6.1) n=17	3 (0.5-23) n=19	2.9 (1.5-4.3) n=14	3 (1.3-4.3) n=17	2.6 (0.7-4.3) n=15	2.9 (1.2-4.1) n=17	1.95 (0.5-4.2) n=14	2.6 (1-3.8) n=17	2.3 (0-7.4) n=11	2.7 (1.7-3.7) n=11
Gamma GT median (range)	70 (18-355)	62 (23-256)	69 (7-408) n=23	66 (23-273)	69 (15-408)	67.5 (22-237) n=20	52 (23-379)	67 (18-350)	54.5 (21-454) n=18	57.5 (15-314) n=18	62 (24-438)	54.5 (15-444) n=18	69 (22-371)	48 (16-342) n=19	74 (24-411) n=13	60 (14-205) n=15

Variable	Baseline (Visit 2) Median (range)		1 week +/- 2 days (Visit 3) Median (range)		4 weeks (visit 4) Median (range)		24 weeks (visit 5) Median (range)		48 weeks (Visit 6) Median (range)		72 weeks (visit 7) Median (range)		96 weeks (Visit 8) Median (range)		108 weeks (visit 9) Median (range)	
	Losartan	Placebo	Losartan	Placebo	Losartan	Placebo	Losartan	Placebo	Losartan	Placebo	Losartan	Placebo	Losartan	Placebo	Losartan	Placebo
**N**	**24**	**21**	**24**	**21**	**23**	**21**	**23**	**21**	**20**	**19**	**19**	**19**	**20**	**21**	**16**	**18**
Weight median (range)	85.1 (74.2-121)	96.7 (61.6-132.5)			85.4 (73.6-127.2)	97.8 (55.4-131.6)	85.8 (73.3-126.8)	93.2 (61.4-132.6)	86 (72.9-121) n=19	94 (60.4-130.1)	85.3 (74.7-120)	94.15 (59.8-129.4) n=18	86.55 (73.5-114)	94.2 (60.3-129.7)	84.45 (72.1-115.5)	95 (60-134)
Waist circumference median (range)	105.85 (96-126)	111.4 (88-136)	106.95 (94.8-134)	113 (84.5-133.5)	107 (93.7-124)	110 (86-135)	110 (94.5-124)	112 (87.5-142)	108 (91-125) n=19	108 (84.2-134)	108 (91.4-134.6)	114.75 (92-135) n=18	108.5 (96-129)	112.5 (91-140)	108.5 (96-127.5)	111 (88-132)
Systolic BP median (range)	133.5 (109-165)	127 (115-180)	128 (104-148)	130 (111-166)	128 (103-168)	130 (105-155)	132 (109-156)	132 (107-151)	126 (102-167)	129 (106-150)	125 (94-152)	125 (108-154) n=18	129 (106-153)	125 (111-175)	137 (102-157) n=10	130 (115-151) n=7
Diastolic BP median (range)	78.5 (67-95)	81 (70-100)	73.5 (64-99)	83 (68-97)	76 (63-93)	85 (66-94)	78 (60-107)	80 (69-93)	75.5 (60-90)	85 (65-95)	76 (61-95)	76.5 (70-100) n=18	74 (63-96)	80 (65-96)	76 (65-90) n=10	83 (65-97) n=7

Sitting Heart rate median (range)	75 (59 100)	77 (59-88)	81 (55-106)	80 (62-104)	70 (51-99)	74 (63-94)	81 (53-110)	76 (62-104)	83 (59-102)	72 (50-95)	81 (55-101)	74.5 (60-90) n=18	79 (61-98)	76 (56-90)	86.5 (63-108) n=10	70 (61-85) n=7

**Variable**	**Baseline (Visit 2) Median (range)**		**4 weeks (visit 4) Median (range)**		**24 weeks (visit 5) Median (range)**		**48 weeks (Visit 6) Median (range)**	
	**Losartan**	**Placebo**	**Losartan**	**Placebo**	**Losartan**	**Placebo**	**Losartan**	**Placebo**
**N**	**24**	**21**	**23**	**21**	**23**	**21**	**20**	**19**
CNS								
Normal	22 (91.67%)	19 (90.48%)	22 (95.65%)	19 (90.48%)	23 (100%)	21 (100%)	18 (90%)	16 (84.21%)
Abnormal	1 (4.17%)	0 (0%)	1 (4.35%)	2 (9.52%)	0 (0%)	0 (0%)	1 (5%)	0 (0%)
Not examined	0 (0%)	1 (4.76%)	0 (0%)	0 (0%)	0 (0%)	0 (0%)	0 (0%)	2 (10.53%)
Missing	1 (4.17%)	1 (4.76%)	0 (0%)	0 (0%)	0 (0%)	0 (0%)	1 (5%)	1 (5.26%)
Neck								
Normal	22 (91.67%)	20 (95.24%)	22 (95.65%)	21 (100%)	23 (100%)	20 (95.24%)	17 (85%)	17 (89.47%)
Abnormal	0 (0%)	0 (0%)	0 (0%)	0 (0%)	0 (0%)	0 (0%)	1 (5%)	0 (0%)
Not examined	1 (4.17%)	0 (0%)	1 (4.35%)	0 (0%)	0 (0%)	1 (4.76%)	1 (5%)	1 (5.26%)
Missing	1 (4.17%)	1 (4.76%)	0 (0%)	0 (0%)	0 (0%)	0 (0%)	1 (5%)	1 (5.26%)
HEENT								
Normal	19 (79.17%)	19 (90.48%)	18 (78.26%)	18 (85.71%)	20 (86.96%)	19 (90.48%)	16 (80%)	18 (94.74%)
Abnormal	0 (0%)	0 (0%)				0 (0%)	1 (5%)	0 (0%)

Not examined<br>Missing	4 (16.67%)<br>1 (4.17%)	1 (4.76%)<br>1 (4.76%)	0 (0%)<br>5 (21.74%)<br>0 (0%)	0 (0%)<br>3 (14.29%)<br>0 (0%)	0 (0%)<br>3 (13.04%)<br>0 (0%)	2 (9.52%)<br>0 (0%)	1 (5%)<br>2 (10%)	0 (0%)<br>1 (5.26%)
**Respiratory**<br>Normal<br>Abnormal<br>Not examined<br>Missing	23 (95.83%)<br>0 (0%)<br>0 (0%)<br>1 (4.17%)	20 (95.24%)<br>0 (0%)<br>0 (0%)<br>1 (4.76%)	23 (100%)<br>0 (0%)<br>0 (0%)<br>0 (0%)<br>0 (0%)	21 (100%)<br>0 (0%)<br>0 (0%)<br>0 (0%)	23 (100%)<br>0 (0%)<br>0 (0%)<br>0 (0%)	20 (95.24%)<br>1 (4.76%)<br>0 (0%)<br>0 (0%)	18 (90%)<br>1 (5%)<br>0 (0%)<br>1 (5%)	18 (94.74%)<br>0 (0%)<br>0 (0%)<br>1 (5.26%)
**Cardiovascular**<br>Normal<br>Abnormal<br>Not examined<br>Missing	23 (95.83%)<br>0 (0%)<br>0 (0%)<br>1 (4.17%)	20 (95.24%)<br>0 (0%)<br>0 (0%)<br>1 (4.76%)	23 (100%)<br>0 (0%)<br>0 (0%)<br>0 (0%)	21 (100%)<br>0 (0%)<br>0 (0%)<br>0 (0%)	23 (100%)<br>0 (0%)<br>0 (0%)<br>0 (0%)	21 (100%)<br>0 (0%)<br>0 (0%)<br>0 (0%)	18 (90%)<br>1 (5%)<br>0 (0%)<br>1 (5%)	18 (94.74%)<br>0 (0%)<br>0 (0%)<br>1 (5.26%)
**Gastrointestinal**<br>Normal<br>Abnormal<br>Not examined<br>Missing	23 (95.83%)<br>0 (0%)<br>0 (0%)<br>1 (4.17%)	18 (85.71%)<br>2 (9.52%)<br>0 (0%)<br>1 (4.76%)	21 (91.3%)<br>2 (8.7%)<br>0 (0%)<br>0 (0%)	20 (95.24%)<br>1 (4.76%)<br>0 (0%)<br>0 (0%)	23 (100%)<br>0 (0%)<br>0 (0%)<br>0 (0%)	20 (95.24%)<br>1 (4.76%)<br>0 (0%)<br>0 (0%)	18 (90%)<br>1 (5%)<br>0 (0%)<br>1 (5%)	16 (84.21%)<br>2 (10.53%)<br>0 (0%)<br>1 (5.26%)
**Abdomen**<br>Normal<br>Abnormal	21 (87.5%)<br>2 (8.33%)	18 (85.71%)<br>2 (9.52%)	22 (95.65%)	17 (80.95%)<br>3 (14.29%)	18 (78.26%)	18 (85.71%)<br>3 (14.29%)	17 (85%)<br>2 (10%)	14 (73.68%)<br>4 (21.05%)

Not examined<br>Missing	0 (0%)<br>1 (4.17%)	0 (0%)<br>1 (4.76%)	1 (4.35%)<br>0 (0%)<br>0 (0%)	1 (4.76%)<br>0 (0%)	5 (21.74%)<br>0 (0%)<br>0 (0%)	0 (0%)<br>0 (0%)	0 (0%)<br>1 (5%)	0 (0%)<br>1 (5.26%)
Musculoskeletal<br>Normal<br>Abnormal<br>Not examined<br>Missing	21 (87.5%)<br>0 (0%)<br>2 (8.33%)<br>1 (4.17%)	19 (90.48%)<br>0 (0%)<br>1 (4.76%)<br>1 (4.76%)	18 (78.26%)<br>2 (8.7%)<br>3 (13.04%)<br>0 (0%)	21 (100%)<br>0 (0%)<br>0 (0%)<br>0 (0%)	20 (86.96%)<br>1 (4.35%)<br>2 (8.7%)<br>0 (0%)	21 (100%)<br>0 (0%)<br>0 (0%)<br>0 (0%)	15 (75%)<br>4 (20%)<br>0 (0%)<br>1 (5%)	17 (89.47%)<br>0 (0%)<br>1 (5.26%)<br>1 (5.26%)
Endocrine & Metabolic<br>Normal<br>Abnormal<br>Not examined<br>Missing	22 (91.67%)<br>0 (0%)<br>1 (4.17%)<br>1 (4.17%)	19 (90.48%)<br>0 (0%)<br>1 (4.76%)<br>1 (4.76%)	19 (82.61%)<br>0 (0%)<br>4 (17.39%)<br>0 (0%)	18 (85.71%)<br>0 (0%)<br>3 (14.29%)<br>0 (0%)	20 (86.96%)<br>0 (0%)<br>3 (13.04%)<br>0 (0%)	19 (90.48%)<br>0 (0%)<br>2 (9.52%)<br>0 (0%)	17 (85%)<br>1 (5%)<br>1 (5%)<br>1 (5%)	16 (84.21%)<br>0 (0%)<br>2 (10.53%)<br>1 (5.26%)
Hematopoietic/Lymphatic<br>Normal<br>Abnormal<br>Not examined<br>Missing	20 (83.33%)<br>0 (0%)<br>3 (12.5%)<br>1 (4.17%)	19 (90.48%)<br>0 (0%)<br>1 (4.76%)<br>1 (4.76%)	19 (82.61%)<br>0 (0%)<br>4 (17.39%)<br>0 (0%)	17 (80.95%)<br>0 (0%)<br>4 (19.05%)<br>0 (0%)	20 (86.96%)<br>0 (0%)<br>3 (13.04%)<br>0 (0%)	19 (90.48%)<br>0 (0%)<br>2 (9.52%)<br>0 (0%)	17 (85%)<br>1 (5%)<br>1 (5%)<br>1 (5%)	16 (84.21%)<br>0 (0%)<br>2 (10.53%)<br>1 (5.26%)

Neurological<br>Normal<br>Abnormal<br>Not examined<br>Missing	21 (87.5%)<br>1 (4.17%)<br>1 (4.17%)<br>1 (4.17%)	20 (95.24%)<br>0 (0%)<br>0 (0%)<br>1 (4.76%)	21 (91.3%)<br>1 (4.35%)<br>1 (4.35%)<br>0 (0%)	18 (85.71%)<br>2 (9.52%)<br>0 (0%)<br>1 (4.76%)	22 (95.65%)<br>1 (4.35%)<br>0 (0%)<br>0 (0%)	21 (100%)<br>0 (0%)<br>0 (0%)<br>0 (0%)	18 (90%)<br>1 (5%)<br>0 (0%)<br>1 (5%)	16 (84.21%)<br>0 (0%)<br>2 (10.53%)<br>1 (5.26%)
Dermatological<br>Normal<br>Abnormal<br>Not examined<br>Missing	19 (79.17%)<br>2 (8.33%)<br>2 (8.33%)<br>1 (4.17%)	19 (90.48%)<br>1 (4.76%)<br>0 (0%)<br>1 (4.76%)	20 (86.96%)<br>1 (4.35%)<br>2 (8.7%)<br>0 (0%)	19 (90.48%)<br>1 (4.76%)<br>1 (4.76%)<br>0 (0%)	18 (78.26%)<br>5 (21.74%)<br>0 (0%)<br>0 (0%)	20 (95.24%)<br>1 (4.76%)<br>0 (0%)<br>0 (0%)	15 (75%)<br>2 (10%)<br>2 (10%)<br>1 (5%)	18 (94.74%)<br>0 (0%)<br>0 (0%)<br>1 (5.26%)
Psychiatric/Psychological<br>Normal<br>Abnormal<br>Not examined<br>Missing	19 (79.17%)<br>0 (0%)<br>4 (16.67%)<br>1 (4.17%)	18 (85.71%)<br>0 (0%)<br>2 (9.52%)<br>1 (4.76%)	22 (95.65%)<br>0 (0%)<br>1 (4.35%)<br>0 (0%)	20 (95.24%)<br>0 (0%)<br>1 (4.76%)<br>0 (0%)	19 (82.61%)<br>1 (4.35%)<br>3 (13.04%)<br>0 (0%)	20 (95.24%)<br>0 (0%)<br>1 (4.76%)<br>0 (0%)	16 (80%)<br>1 (5%)<br>2 (10%)<br>1 (5%)	16 (84.21%)<br>0 (0%)<br>2 (10.53%)<br>1 (5.26%)

Variable	72 weeks (Visit 7) Median (range)				96 weeks (visit 8) Median (range)				108 weeks (visit 9) Median (range)			
	Losartan		Placebo		Losartan		Placebo		Losartan		Placebo	
n	19		19		20		21		16		18	
CNS												
Normal	18	(94.74%)	18	(94.74%)	16	(80%)	20	(95.24%)	15	(93.75%)	17	(94.44%)
Abnormal	1	(5.26%)	0	(0%)	0	(0%)	0	(0%)	0	(0%)	0	(0%)
Not examined	0	(0%)	0	(0%)	1	(5%)	1	(4.76%)	0	(0%)	0	(0%)
Missing	0	(0%)	1	(5.26%)	3	(15%)	0	(0%)	1	(6.25%)	1	(5.56%)
Neck												
Normal	19	(100%)	17	(89.47%)	14	(70%)	16	(76.19%)	14	(87.50%)	14	(77.78%)
Abnormal	0	(0%)	0	(0%)	0	(0%)	1	(4.76%)	0	(0%)	0	(0%)
Not examined	0	(0%)	1	(5.26%)	3	(15%)	4	(19.05%)	1	(6.25%)	3	(16.67%)
Missing	0	(0%)	1	(5.26%)	3	(15%)	0	(0%)	1	(6.25%)	1	(5.56%)
HEENT												
Normal	16	(84.21%)	15	(78.95%)	15	(75%)	18	(85.71%)	14	(87.50%)	15	(83.33%)
Abnormal	0	(0%)	0	(0%)	0	(0%)	0	(0%)	0	(0%)	0	(0%)
Not examined	3	(15.79%)	3	(15.79%)	2	(10%)	3	(14.29%)	1	(6.25%)	2	(11.11%)
Missing	0	(0%)	1	(5.26%)	3	(15%)	0	(0%)	1	(6.25%)	1	(5.56%)

Respiratory<br>Normal<br>Abnormal<br>Not examined<br>Missing	19 (100%)<br>0 (0%)<br>0 (0%)<br>0 (0%)	18 (94.74%)<br>0 (0%)<br>0 (0%)<br>1 (5.26%)	17 (85%)<br>0 (0%)<br>0 (0%)<br>3 (15%)	21 (100%)<br>0 (0%)<br>0 (0%)<br>0 (0%)	15 (93.75%)<br>0 (0%)<br>0 (0%)<br>1 (6.25%)	17 (94.44%)<br>0 (0%)<br>0 (0%)<br>1 (5.56%)
Cardiovascular<br>Normal<br>Abnormal<br>Not examined<br>Missing	19 (100%)<br>0 (0%)<br>0 (0%)<br>0 (0%)	18 (94.74%)<br>0 (0%)<br>0 (0%)<br>1 (5.26%)	17 (85%)<br>0 (0%)<br>0 (0%)<br>3 (15%)	21 (100%)<br>0 (0%)<br>0 (0%)<br>0 (0%)	15 (93.75%)<br>0 (0%)<br>0 (0%)<br>1 (6.25%)	16 (88.89%)<br>1 (5.56%)<br>0 (0%)<br>1 (5.56%)
Gastrointestinal<br>Normal<br>Abnormal<br>Not examined<br>Missing	19 (100%)<br>0 (0%)<br>0 (0%)<br>0 (0%)	16 (84.21%)<br>2 (10.53%)<br>0 (0%)<br>1 (5.26%)	15 (75%)<br>2 (10%)<br>0 (0%)<br>3 (15%)	20 (95.24%)<br>0 (0%)<br>1 (4.76%)<br>0 (0%)	15 (93.75%)<br>0 (0%)<br>0 (0%)<br>1 (6.25%)	16 (88.89%)<br>0 (0%)<br>1 (5.56%)<br>1 (5.56%)
Abdomen<br>Normal<br>Abnormal<br>Not examined<br>Missing	17 (89.47%)<br>2 (10.53%)<br>0 (0%)<br>0 (0%)	18 (94.74%)<br>0 (0%)<br>0 (0%)<br>1 (5.26%)	15 (75%)<br>2 (10%)<br>0 (0%)<br>3 (15%)	19 (90.48%)<br>2 (9.52%)<br>0 (0%)<br>0 (0%)	14 (87.50%)<br>1 (6.25%)<br>0 (0%)<br>1 (6.25%)	16 (88.89%)<br>1 (5.56%)<br>0 (0%)<br>1 (5.56%)

Musculoskeletal												
Normal	16	(84.21%)	17	(89.47%)	13	(65%)	19	(90.48%)	15	(93.75%)	16	(88.89%)
Abnormal	2	(10.53%)	0	(0%)	2	(10%)	1	(4.76%)	0	(0%)	0	(0%)
Not examined	1	(5.26%)	1	(5.26%)	2	(10%)	1	(4.76%)	0	(0%)	1	(5.56%)
Missing	0	(0%)	1	(5.26%)	3	(15%)	0	(0%)	1	(6.25%)	1	(5.56%)
Endocrine & Metabolic												
Normal	16	(84.21%)	14	(73.68%)	14	(70%)	17	(80.95%)	13	(81.25%)	12	(66.67%)
Abnormal	0	(0%)	0	(0%)	0	(0%)	1	(4.76%)	0	(0%)	0	(0%)
Not examined	3	(15.79%)	4	(21.05%)	3	(15%)	3	(14.29%)	2	(12.50%)	5	(27.78%)
Missing	0	(0%)	1	(5.26%)	3	(15%)	0	(0%)	1	(6.25%)	1	(5.56%)
Hematopoietic/Lymphatic												
Normal	15	(78.95%)	16	(84.21%)	15	(75%)	17	(80.95%)	13	(81.25%)	11	(61.11%)
Abnormal	0	(0%)	0	(0%)	0	(0%)	0	(0%)	0	(0%)	0	(0%)
Not examined	4	(21.05%)	2	(10.53%)	2	(10%)	4	(19.05%)	2	(12.50%)	6	(33.33%)
Missing	0	(0%)	1	(5.26%)	3	(15%)	0	(0%)	1	(6.25%)	1	(5.56%)
Neurological												
Normal	18	(94.74%)	18	(94.74%)	16	(80%)	19	(90.48%)	15	(93.75%)	14	(77.78%)
Abnormal	1	(5.26%)	0	(0%)			0	(0%)	0	(0%)	0	(0%)
Not												

examined	0 (0%)	0 (0%)	0 (0%)	2 (9.52%)	0 (0%)	3 (16.67%)
Missing	0 (0%)	1 (5.26%)	1 (5%)	0 (0%)	1 (6.25%)	1 (5.56%)
			3 (15%)			
Dermatological						
Normal	17 (89.47%)	17 (89.47%)	14 (70%)	17 (80.95%)	12 (75.00%)	13 (72.22%)
Abnormal	2 (10.53%)	1 (5.26%)	1 (5%)	0 (0%)	2 (12.50%)	0 (0%)
Not examined	0 (0%)	0 (0%)	2 (10%)	4 (19.05%)	1 (6.25%)	4 (22.22%)
Missing	0 (0%)	1 (5.26%)	3 (15%)	0 (0%)	1 (6.25%)	1 (5.56%)
Psychiatric/Psychological						
Normal	15 (78.95%)	14 (73.68%)	13 (65%)	16 (76.19%)	12 (75%)	11 (61.11%)
Abnormal	1 (5.26%)	0 (0%)	1 (5%)	0 (0%)	1 (6.25%)	0 (0%)
Not examined	3 (15.79%)	4 (21.05%)	3 (15%)	5 (23.81%)	2 (12.50%)	6 (33.33%)
Missing	0 (0%)	1 (5.26%)	3 (15%)	0 (0%)	1 (6.25%)	1 (5.56%)

Haematology

Variable	Baseline (Visit 2) Median (range)		4 weeks (visit 4) Median (range)		24 weeks (visit 5) Median (range)		48 weeks (Visit 6) Median (range)		72 weeks (visit 7) Median (range)		96 weeks (Visit 8) Median (range)		108 weeks (visit 9) Median (range)	
	**Losartan**	**Placebo**	**Losartan**	**Placebo**	**Losartan**	**Placebo**	**Losartan**	**Placebo**	**Losartan**	**Placebo**	**Losartan**	**Placebo**	**Losartan**	**Placebo**
**n**	**24**	**21**	**23**	**21**	**23**	**21**	**20**	**19**	**19**	**19**	**20**	**21**	**16**	**18**
Haemoglobin median (range)	14.6 (12.3-142) n=23	14.9 (13.2-18)	14.3 (11.7-137)	14.6 (13-161)	13.75 (11.8-16.4) n=22	15 (11.8-159)	14.05 (12.2-155)	15.1 (5.7-138) n=18	14.1 (11.8-163) n=18	15.4 (12-165)	14.35 (11.9-131)	14.7 (12.8-136)	14.7 (11-125) n=14	14.75 (12.8-138)
Leukocytes WBC median (range)	7.2 (4.3-13.1) n=23	7.3 (4.4-12.3)	7 (5-13.6)	7 (3.7-12.7)	7.4 (4.7-15.2) n=22	7.8 (4.3-11.1)	7.3 (2-11.3)	6.75 (1.4-12.3) n=18	7.25 (4.5-12.9) n=18	7.1 (4.5-12.9) n=17	7.2 (2.5-13.2)	7 (2.7-13.8)	6.85 (5.2-13.9) n=14	7 (4.1-14.9)
Platelets median (range)	224 (137-360) n=23	224 (158-404)	230 (123-349)	224 (148-414)	233 (142-357) n=22	220 (153-376)	228.5 (122-341)	219 (153-332) n=18	221 (125-334)	213 (157-332)	224 (135-362)	209 (132-349)	225 (128-398) n=14	223 (144-335) n=17
MCV median (range)	89.55 (76.9-99) n=22	87.3 (80.2-94.3)	90 (78.3-100)	88.8 (79.4-94)	90.7 (78.2-99)	87 (78.6-92.8)	89.9 (9-100)	85.95 (10.4-93.1) n=18	90.6 (82.7-99)	86.3 (79.5-94.6)	90.2 (76.2-98)	85.8 (80-95.4) n=20	89.25 (75.4-97) n=14	86.1 (81.2-96.3)
HBA1C mmol median (range)	53.5 (34-81) n=22	42 (31-81)	61.5 (42-79) n=10	47 (37-61) n=7	64 (37-119) n=13	50 (31-97) n=13	57 (34-87) n=19	43 (32-86) n=17	49 (36-72) n=10	48 (37-115) n=10	51 (38-91) n=16	41 (33-85) n=15	51 (37-65) n=3	49 (41-83) n=6

Prothrombus Time median (range)	11 (1-15) n=23	11 (10-22) n=20	10 (1-15) n=20	11 (9-13)	11 (1-32) n=20	11 (10-14) n=20	11 (1-11) n=17	11 (9-13) n=17	11 (1-13) n=17	11 (10-13) n=17	10 (1-14) n=17	11 (10-13) n=20	10 (1-12) n=13	11 (9-14) n=17

Table 24: Estimates for the median change from baseline to 96 weeks by randomised treatment arm.

Variable	n*	Losartan (n=15)<br>Change from baseline to 96 weeks (Visit 8)	n*	Placebo (n=17)<br>Change from baseline to 96 weeks (Visit 8)
Diagnostic Category<br>Median<br>(IQR),(Range)	15	 0<br>(0,0),(-1,1)	17	 0<br>(0,0),(-1,0)
Grade of Steatosis<br>Median<br>(IQR),(Range)	15	 0<br>(-1,0),(-2,1)	17	 0<br>(-1,0),(-2,0)
% Steatotic hepatocytes<br>Mean (sd)<br>Median<br>(IQR),(Range)	15	 -6.67 (25.82)<br>-10<br>(-60,50), (-20,10)	17	 -20 (26.22)<br>-20<br>(-40,0),(-70,20)
Hepatocyte Ballooning Median<br>(IQR),(Range)	15	 0<br>(-1,0),(-1,1)	17	 0<br>(-1,0),(-1,1)
Lobular inflammation<br>Median<br>(IQR),(Range)	15	 0<br>(0-0),(-1,1)	17	 0<br>(-1,0),(-2,1)
SAF Lobular inflammation Median<br>(IQR),(Range)	15	 0<br>(-1,0),(-1,1)	17	 0<br>(-1,0) ,(-1,1)
NAFLD Score (NAS) Kleiner Median<br>(IQR),(Range)	15	 0<br>(-2,0),(-3,3)	17	 -1<br>(-3,0),(-4,1)
FLIP Activity score<br>Median<br>(IQR),(Range)	15	 0<br>(-1,0), (-2,1)	17	 -1<br>(-1,0),(-2,2)
Global Grade-Brunt 1999 Median<br>(IQR),(Range)	15	 -1<br>(-1,0),(-1,1)	17	 -1<br>(-1,0),(-2,0)
Sinusoidal/pericellular fibrosis Median<br>(IQR),(Range)	15	 0<br>(0,0),(-1,2)	17	 0<br>(-1,0),(-2,1)
7-tier staging system<br>Median<br>(IQR),(Range)	15	 0<br>(0,0),(-2,2)	17	 0<br>(-1,0),(-2,1)
Portal Inflammation<br>Median<br>(IQR),(Range)	15	 0<br>(0,0),(-1,2)	17	 0<br>(0,1),(-1,1)
Apoptotic bodies<br>Median<br>(IQR),(Range)	15	 0<br>(0,0),(-1,1)	17	 0<br>(0,0),(-1,1)
Mallory-Denk bodies<br>Median<br>(IQR),(Range)	14	 0<br>(-1,0),(-1,0)	17	 0<br>(-1,0),(-2,1)

n* = number of patients with both baseline and 96 week measure.

Correlation between measures of fibrosis and biomarkers obtained non-invasively

This is an important research question but numbers were too small to be meaningful.

Quality of life

The median (range) of baseline QoL scores is calculated and presented by randomised group for each domain. The median changes in QoL (based on overall score) from baseline to 24-months are calculated and presented graphically using boxplots by randomisation group. This is carried out separately for CLDQ and SF-36.

Table for SF-36 change from baseline summary measures

	**Change from baseline (Visit 8-visit 2) Median (Range) (IQR)**	
**Variable**	**Losartan n=18**	**Placebo n=19**
PCS	-1.47 (-14.19,4.28) (-6.44,2.08)	-2.90 (-15.36,28.61) (-7.35,2.49)
MCS	1.10 (-30.05-16.38) (-2.40,3.86)	-0.12 (-20.95,21.56) (-6.91,3.20)
PCS_obl	-1.75 (-20.6,5.83) (-5.92,2.14)	-2.83 (-11.87,21.75) (-6.18,1.41)
MCS_obl	-0.22 (-32.4,14.7) (-3.95,4.21)	-0.55 (-12.61,17.78) (-8.56,2.22)

Corresponding Boxplot for Sf-36 change from BL for summary measures

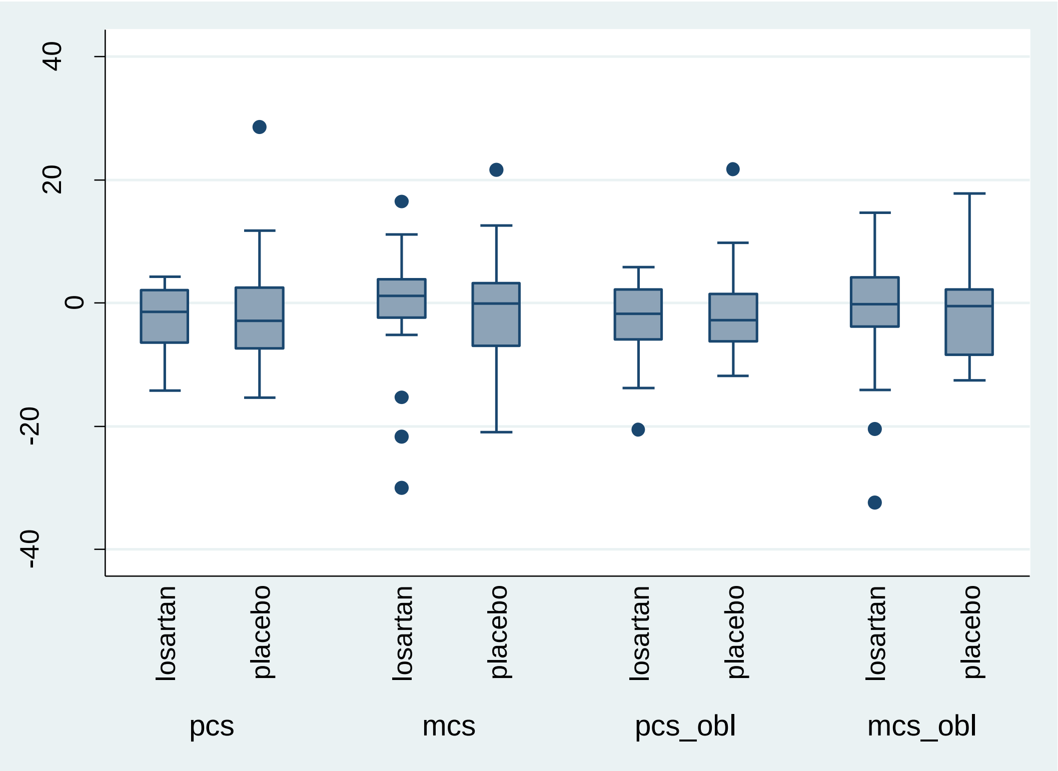

CLDQ data (based on 1 missing element then domain missing)

	Change from BL to visit 8 median (range) (IQR)	
	**Losartan**	**Placebo**
AS	0.00 (-3.67,2.00) (-1.33,0.00) n=17	0.00 (-2.33,3.67) (-0.33,0.33) n=19
FA	-0.60 (-1.80,0.40) (-0.90,0.00) n=16	-0.20 (-1.60,0.80) (-0.80,0.40) n=20
SS	-0.50 (-1.60,0.40) (-0.90,-0.10) n=16	-0.30 (-1.40,1.40) (-0.60,0.00) n=20
AC	-0.33 (-3.33,2.33) (-1.33,0.00) n=17	-0.33 (-3.00,2.00) (-0.67,0.17) n=20
EF	0.00 (-3.63,1.13) (-0.75,0.25) n=17	-0.13 (-1.75,1.00) (-0.75,0.50) n=18
WO	0.00 (-3.60,1.20) (-0.20,0.60) n=17	0.10 (-2.20,1.20) (-0.50,0.80) n=20
Overall	-0.17 (-2.28,0.38) (-0.48,0.07) n=15	-0.10 (-1.07,0.79) (-0.59,0.14) n=17

CLDQ boxplot (based on 1 missing element then domain missing)

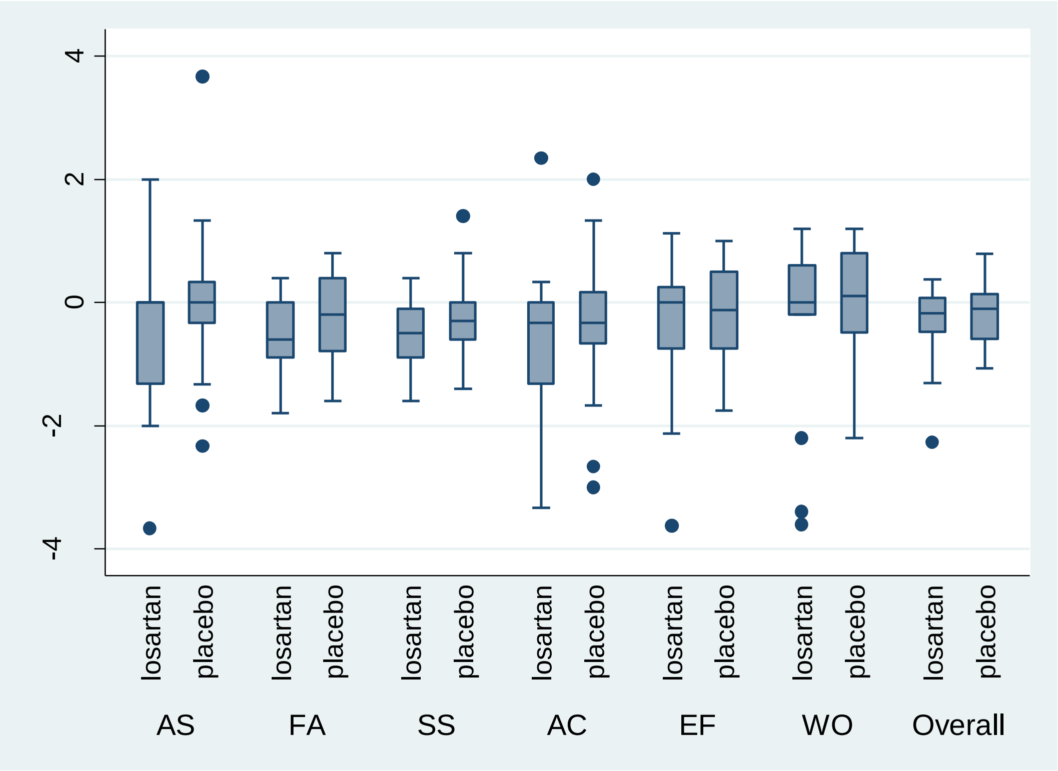

7.4 Informing future studies

- Based on Feasibility (and screening)

7.5 Statistical Software

Trial data were input by individual site staff into a MACRO database held and maintained by the Newcastle Clinical Trials Unit.

Data were downloaded directly from MACRO into statistical software packages including SAS, Stata and R. Statistical analyses was carried out by the Trial Statisticians downloading snapshots of the data at time-points agreed by the TMG.

8. STUDY REPORT/DISSEMINATION OF RESULTS

The data will be the property of the Chief Investigator and Co-Investigator(s). Publication will be the responsibility of the Chief Investigator on behalf of TMF and co-investigators.

It is planned to publish this study in peer-reviewed journals. Results of the study will also be reported to the sponsor and funder(s). A copy of the study final report will be provided to both NIHR EME and BRC. A copy of the final report will also be forwarded to REC (Research Ethics Committee). The EudraCT Final Report will be prepared and uploaded to the EudraCT database outlining the results of the study.

Participants will be provided with a lay summary of the results on request.

9. STORAGE AND ARCHIVING

The trial database is stored at the Newcastle University. Snapshots of the database are kept on the NCTU server which is backed up daily. Once all trial related analysis and activities are completed the Database is taken off line stored on a password protected disc and archived according to the current NCTU SOPs.

Appendix: Table of units

Variable	Units
Height	Cm
Weight	Kg
Waist	Cm
Sitting blood pressure	mmHg
Sitting heart rate	bpm
Sodium<br>Potassium<br>Urea<br>Glucose<br>Triglyceride<br>HDL cholesterol<br>Total cholesterol<br>LDL cholesterol<br>HBA1C	mmol/L
AST<br>ALT<br>Alkaline Phosphatase<br>Gamma GT<br>insulin	U/L
Creatinine<br>Total bilirubin	umol/L
Albumin<br>immumoglobulins	g/L
Haemoglobin	g/dL
Laukocytes (WBC)<br>Platelets	X10^9/L
MCV	fL
Prothrombin time	seconds
Apolipoproteins A	µg/L
Alpha-2 macroglobulin	mg/100ml
Haptoglobins<br>C-peptide	
Liver stiffness	kpa

Data analysis for the FELINE paper

It was brought to our attention that 4 patients should be removed from the visit 8 descriptive statistics by a reviewer of the FELINE paper (February 2017). These patients decided to withdraw from the study and had agreed to one more study visit for safety and this data was put on a visit 8 (96 weeks) form in the database, however the data was taken at a time too early (between 6 months to a year after randomisation) for a visit 8 which is at 2 years. As the end of trial report has been submitted we have edited the CONSORT diagram to better reflect what happened in the trial and taken out these 4 patients in the visit 8 summaries for the paper. Note that these 4 patients did not have a biopsy so this has no effect on the primary outcome data. Note that the MACRO data was not amended due to site closedown.

CONSORT diagram for paper: Consort diagram with the 4 patients removed from visit 8. This corresponds to Figure 1.

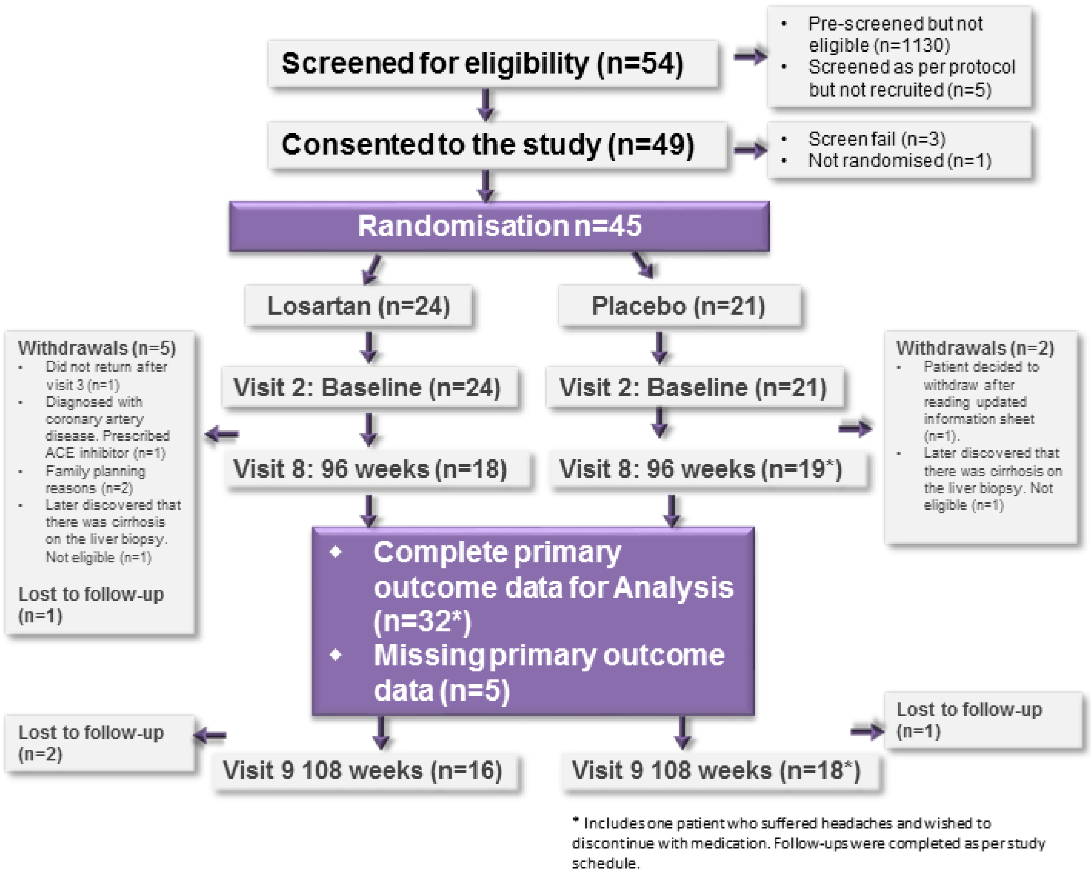

More detailed CONSORT diagram: corresponds to Figure 1 (less detailed CONSORT used for paper).

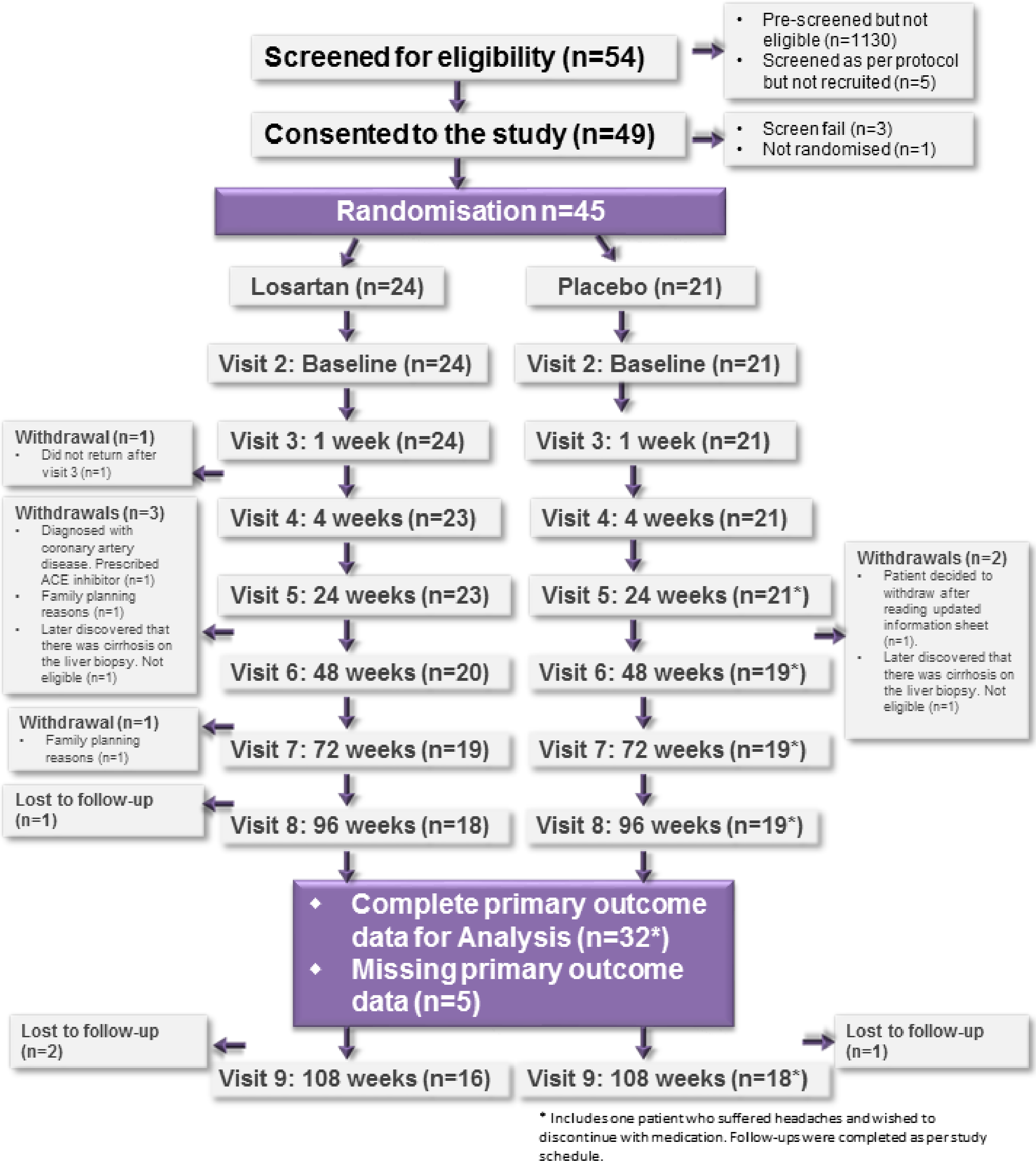

Boxplot for paper : corresponds to CLQQ boxplot on page 65.

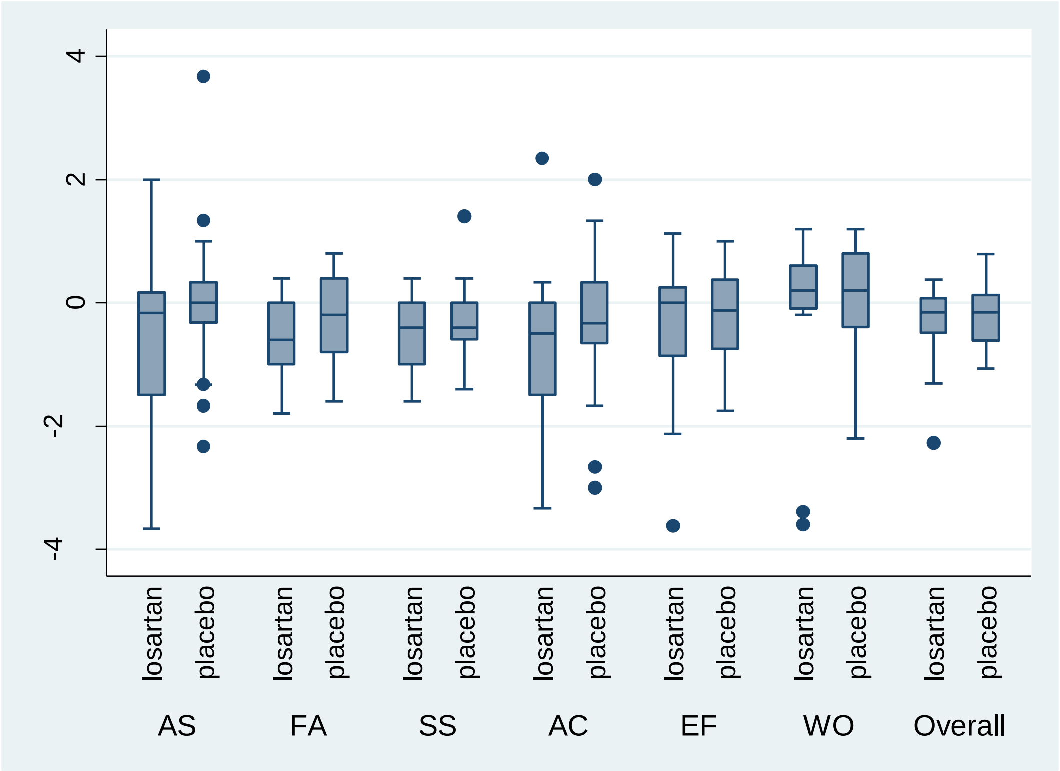

Table for paper: Summary statistics with 4 patients removed from visit 8. This table recalculates some of the information given in Table 23 from the main body of the report.

	Losartan				Placebo			
	Baseline (N=24)		96 weeks (N=18)		Baseline (N=21)		96 weeks (N=19)	
Variable	n	Median (range)	n	Median (range)	n	Median (range)	n	Median (range)
Weight (kg)	24	85.1 (74.2-121.0)	18	86.6 (73.5-113.9)	21	96.7 (61.6-132.5)	19	94.2 (60.3-129.7)
Waist circumference (cm)	24	105.9 (96.0-126.0)	18	108.0 (96.0-129.0)	21	111.4 (88.0-136.0)	19	112.5 (93.0-131.0)
AST (U/L)	21	35 (14-102)	17	31 (2-70)	18	46 (30-70)	16	36 (17-110)
ALT (U/L)	24	52.5 (21-136)	18	32.5 (7-80)	21	65 (33-135)	18	52 (18-133)
ALP (U/L)	24	89.5 (44-173)	18	89.5 (37-191)	21	72 (49-116)	17	79 (46-106)
Gamma GT (U/L)	24	70 (18-355)	18	55.5 (22-371)	21	62 (23-256)	17	48 (16-342)
ELF test	23	8.8 (6.5-11.8)	18	9.4 (7.9-11.0)	19	8.0 (6.4-10.3)	15	9.1 (7.3-10.8)
Creatinine (umol/L)	24	75.5 (48-105)	18	73.5 (43-106)	21	72 (5-97)	17	68 (52-94)
Triglyceride (mmol/L)	23	1.7 (0.9-7.9)	17	2 (0.8-6.5)	19	2.0 (0.4-4.4)	18	1.7 (0.9-4.4)
HDL Cholesterol (mmol/L)	22	1.1 (0.7-2.8)	16	1.0 (0.8-1.9)	20	1.1 (0.8-3.5)	16	1.1 (0.8-1.8)
Total Cholesterol (mmol/L)	23	4.3 (2.1-7.5)	16	4.0 (2.2-7.6)	20	4.6 (1.0-6.5)	18	4.4 (2.7-6.5)
LDL Cholesterol (mmol/L)	19	2.5 (0.8-3.8)	13	1.9 (0.5-4.2)	18	3.2 (1.2-4.4)	15	2.6 (1.0-3.8)
Glucose (mmol/L)	24	6.0 (4.4-17.1)	18	8.1 (4.8-19.7)	20	6.2 (3.6-15.9)	18	6.7 (4.3-15.8)
Systolic BP	24	133.5 (109-165)	18	129 (106-152)	21	127 (115-180)	19	125 (111-175)
Diastolic BP	24	78.5 (67-95)	18	74 (63-96)	21	81 (70-100)	19	80 (65-96)
Platelets (x10^9/L)	23	224 (137-360)	18	224 (135-362)	21	224 (158-404)	19	209 (155-349)
